# The effect of SGLT2 inhibition on prostate cancer: Mendelian randomization and observational analysis using electronic healthcare and cohort data

**DOI:** 10.1016/j.xcrm.2024.101688

**Published:** 2024-08-20

**Authors:** Jie Zheng, Jieli Lu, Jiying Qi, Qian Yang, Huiling Zhao, Haoyu Liu, Zhihe Chen, Lanhui Huang, Youqiong Ye, Min Xu, Yu Xu, Tiange Wang, Mian Li, Zhiyun Zhao, Ruizhi Zheng, Shuangyuan Wang, Hong Lin, Chunyan Hu, Celine Sze Ling Chui, Shiu Lun Au Yeung, Shan Luo, Olympia Dimopoulou, Padraig Dixon, Sean Harrison, Yi Liu, Jamie Robinson, James Yarmolinsky, Philip Haycock, Jinqiu Yuan, Sarah Lewis, Zhongshang Yuan, Tom R. Gaunt, George Davey Smith, Guang Ning, Richard M. Martin, Bin Cui, Weiqing Wang, Yufang Bi

**Affiliations:** 1 Department of Endocrine and Metabolic Diseases, Shanghai Institute of Endocrine and Metabolic Diseases, Ruijin Hospital, Shanghai Jiao Tong University School of Medicine, Shanghai, China; 2 Shanghai National Clinical Research Center for metabolic Diseases, Key Laboratory for Endocrine and Metabolic Diseases of the National Health Commission of the PR China, Shanghai Key Laboratory for Endocrine Tumor, Shanghai Digital Medicine Innovation Center, Ruijin Hospital, Shanghai Jiao Tong University School of Medicine, Shanghai, China; 3 MRC Integrative Epidemiology Unit (IEU), Bristol Medical School, University of Bristol, Oakfield House, Oakfield Grove, Bristol BS8 2BN, UK; 4 Center for Immune-Related Diseases at Shanghai Institute of Immunology, Department of Gastroenterology, Ruijin Hospital, Shanghai Jiao Tong University School of Medicine, Shanghai, China; 5 Shanghai Institute of Immunology, State Key Laboratory of Oncogenes and Related Genes, Department of Immunology and Microbiology, Shanghai Jiao Tong University School of Medicine, Shanghai 200025, China; 6 School of Nursing, Li Ka Shing Faculty of Medicine, The University of Hong Kong, Hong Kong Special Administration Region, China; 7 School of Public Health, Li Ka Shing Faculty of Medicine, The University of Hong Kong, Hong Kong Special Administration Region, China; 8 Laboratory of Data Discovery for Health (D24H), Hong Kong Science Park, Hong Kong Science and Technology Park, Hong Kong Special Administration Region, China; 9 Population Health Sciences, Bristol Medical School, University of Bristol, Bristol BS8 2PS, UK; 10 Nuffield Department of Primary Care Health Sciences, University of Oxford, Oxford OX2 6GG, UK; 11 Department of Population Health Sciences, Bristol Medical School, University of Bristol, Bristol BS8 2BN, UK; 12 Clinical Research Center, The Seventh Affiliated Hospital, Sun Yat-sen University, Shenzhen, Guangdong 518107, China; 13 Center for Digestive Disease, The Seventh Affiliated Hospital, Sun Yat-sen University, Shenzhen, Guangdong 518107, China; 14 Guangzhou Women and Children Medical Center, Guangzhou, Guangdong 510623, China; 15 Division of Epidemiology, the JC School of Public Health & Primary Care, the Chinese University of Hong Kong, Hong Kong; 16 Department of Biostatistics, School of Public Health, Cheeloo College of Medicine, Shandong University, Jinan, China; 17 NIHR Biomedical Research Centre at the University Hospitals Bristol and Weston NHS Foundation Trust and the University of Bristol, Bristol, UK

**Keywords:** SGLT2 inhibition, prostate cancer, Mendelian randomization, observational analysis, electronic healthcare records, cohort study

## Abstract

We evaluated the effect of sodium-glucose cotransporter 2 (SGLT2) inhibition on prostate cancer by evidence triangulation. Using Mendelian randomization, we found that genetically proxied SGLT2 inhibition reduced the risk of overall (odds ratio = 0.56, 95% confidence interval [CI] = 0.38 to 0.82; 79,148 prostate cancer cases and 61,106 controls), advanced, and early-onset prostate cancer. Using electronic healthcare data (*n*_SGLT2i_ = 24,155; *n*_DPP4i_ = 24,155), we found that the use of SGLT2 inhibitors was associated with a 23% reduced risk of prostate cancer (hazard ratio = 0.77, 95% CI = 0.61 to 0.99) in men with diabetes. Using data from two prospective cohorts (*n*_4C_ = 57,779; *n*_UK_Biobank_ = 165,430), we found little evidence to support the association of HbA_1c_ with prostate cancer, implying a non-glycemic effect of SGLT2 inhibition on prostate cancer. In summary, this study provides multiple layers of evidence to support the beneficial effect of SGLT2 inhibition on reducing prostate cancer risk. Future trials are warranted to investigate whether SGLT2 inhibitors can be recommended for prostate cancer prevention.

## Highlights

• A causal protective effect of SGLT2 inhibition on the risk of prostate cancer was observed
• This is likely to be a non-glycemic effect of SGLT2 inhibition on prostate cancer
• The prescription of SGLT2 inhibitors was prioritized for those with prostate cancer risk

## Graphical abstract

**Figure:**
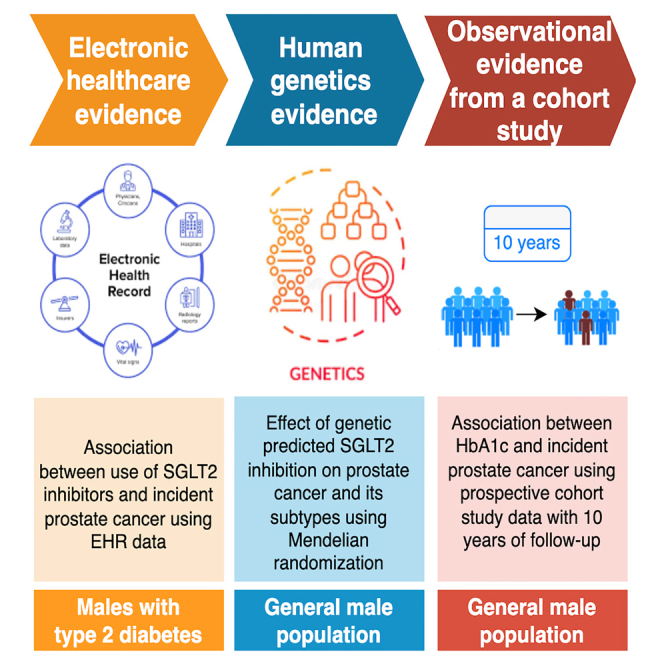

## Teaser

Zheng et al. combined genetic, real-world, and cohort evidence to show a causal protective effect of SGLT2 inhibition on the risk of prostate cancer and showed that this effect is likely through a non-glycemic pathway. This study prioritized the prescription of SGLT2 inhibitors for those with prostate cancer risk.

## Introduction

Diabetes is one of the most common chronic conditions, affecting 537 million individuals in 2021.^1^ Among various types of anti-diabetic drugs, recent clinical trials have demonstrated the beneficial effect of sodium-glucose cotransporter 2 (SGLT2) inhibitors in reducing the risk of atherosclerotic cardiovascular disease (ASCVD) in addition to improvements in HbA_1c_.^2^^,^^3^^,^^4^ Based on the robust trial evidence, the American Diabetes Association and European Association for the Study of Diabetes guidelines have, since 2020, recommended SGLT2 inhibitors as first-line therapy for patients with or at high risk for ASCVD, heart failure, or chronic kidney disease.^5^ It has now been widely used by clinicians from endocrinology and cardiology departments.

Cancer is recognized as a common comorbidity for type 2 diabetes mellitus (T2DM).^6^ Among various cancer types, prostate cancer is the second most commonly diagnosed malignancy in men, with nearly 1.41 million new cases reported worldwide in 2020, and is a major cause of cancer death in men.^7^ However, no clinical guideline recommends the use of anti-diabetic drugs for individuals with cancers or those at high risk of developing cancers, especially for males with both diabetes and prostate cancer. A recent review has summarized the anti-cancer mechanisms of SGLT2 inhibitors.^8^ Observational studies have also reported a decreased risk of prostate cancer among men with diabetes who are taking SGLT2 inhibitors.^9^ However, the largest meta-analysis of randomized controlled trials (RCTs) in individuals with T2DM suggested little difference in prostate cancer incidence between users of SGLT2 inhibitors and users of placebo or active comparators.^10^ Notably, this study’s statistical power might be limited due to the small number of incident prostate cancer cases (*n* = 41) included in the analysis. Collectively, existing epidemiology studies provide some clues, but the evidence supporting the protective effect of SGLT2 inhibition on prostate cancer risk remains insufficient. Whether SGLT2 inhibition can be recommended for diabetic individuals at high risk of cancers or potentially repurposed as an anti-cancer therapeutic target needs further investigation.

Evidence triangulation is the practice of obtaining more reliable answers to research questions through integrating results from several different methods.^11^ These methods have different assumptions and unrelated sources of biases. If results of these methods point to a similar conclusion, this will strengthen confidence in the finding. For the causal question aimed at identifying the effect of a drug target on a disease, human genetics, electronic healthcare, and cohort data are commonly employed data sources.^12^^,^^13^ Triangulating evidence from these methods in a single study may provide an attractive strategy to improve evidence level for drug repurposing. Mendelian randomization (MR) is a method that utilizes germline genetic variants as proxy measures of exposure to estimate the causal effect of an exposure on an outcome.^14^ An individual’s germline genetic makeup influences their biology from conception, meaning that causal estimates from MR studies reflect lifelong exposures (e.g., lifelong SGLT2 inhibition) and are generally not susceptible to reverse causation or confounding.^15^ Observational associations regarding the use of a drug on disease incidence are normally estimated using Cox proportional hazard models, where a “new user active comparators” design may reduce the influence of confounders.^16^ Prospective cohort studies provide observational associations between an exposure and an outcome, which may be influenced by confounding factors. Due to the availability of enriched data sources supporting the application of all three methods,^17^^,^^18^ studying the effect of SGLT2 inhibition on prostate cancer serves as a preferred example for evidence triangulation.

The objective of this study was to estimate the causal effects of SGLT2 inhibition on prostate cancer and its subtypes by triangulating evidence from human genetics, electronic healthcare, and biological data. The effect of HbA_1c_ on prostate cancer was further estimated using human genetics and observational epidemiology approaches.

## Results

### Summary of study design and data sources

**Figure 1:**
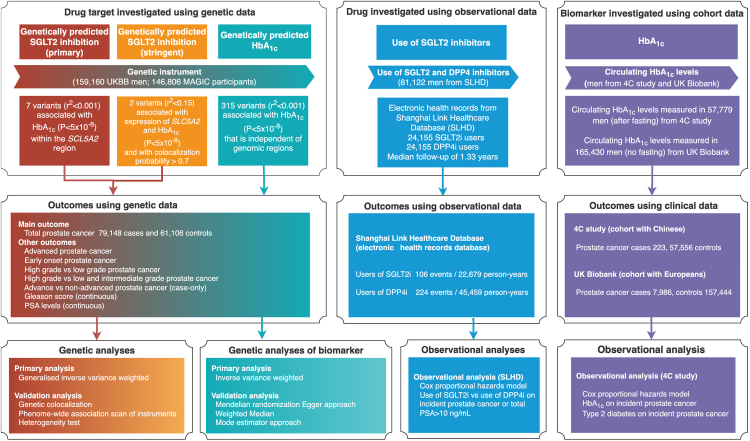
Genetic instrument selection, data sources, and analysis strategy in a triangulation study of the effect of SGLT2 inhibition on prostate cancer For human genetic analyses, the effect of sodium-glucose cotransporter 2 (SGLT2) inhibition on the risk of prostate cancer and its subtypes were estimated using Mendelian randomization. For observational analyses, the effect of use of SGLT2 inhibitors on incident prostate cancer risk was estimated in males with diabetes. DPP4 inhibitors were used as active comparators. For observational analysis of biomarker, the association of HbA1c on incident prostate cancer was estimated in UK Biobank and 4C study. More details of instrument selection and analysis strategies were listed in the STAR Methods, instrument selection and Mendelian randomization analyses.

Figure 1 presents an overview of three sets of analyses conducted in this study. Each analysis aims to answer the same causal question in different subpopulations. All studies contributing data to this analysis had the relevant institutional review board approval from each country, and all participants provided informed consent.

First, the association of the use of SGLT2 inhibitors with incident prostate cancer was estimated in diabetic individuals using data derived from electronic health record data in the Shanghai Link Healthcare Database (SLHD; *n* = 81,122 men with diabetes; Table S1), a representative clinical database covering electronic healthcare records for over 99% of Shanghai residents since 2013^19^ (more details in the STAR Methods, expermential model and subject details).

Second, the human genetics analysis was applied in the general male population. We estimated the putative causal effects of SGLT2 inhibition and genetically predicted HbA_1c_ on the risks of prostate cancer and its subtypes using MR (Tables S2, S3, and S4; Figure S1). The summary genetic association data from a case-control genome-wide association study (GWAS) of prostate cancer in the PRACTICAL and GAME-ON/ELLIPSE Consortium^17^^,^^18^ were used (*n* = 140,254 men from the general population; Table S5; more details in the STAR Methods, the PRACTICAL and GAME-ON/ELLIPSE Consortium). MR has three key assumptions (Figure S2): (1) the germline genetic instruments used to proxy SGLT2 inhibition are robustly associated with the exposure (“relevance”); (2) there is no confounding of the relationship between the instruments and the outcome (“independence”); and (3) the instruments are only associated with the outcome through the exposure under study (“exclusion restriction”). The validity of these assumptions was tested using a set of sensitivity analyses.

Third, the association of baseline HbA_1c_ levels with incident prostate cancer during 10 years of follow-up was estimated using data from the China Cardiometabolic and Cancer Cohort (4C) study^6^ (*n* = 57,779 men from the general population; more details in the STAR Methods, the China Cardiometabolic and Cancer Cohort (4C) study) and UK Biobank (*n* = 165,430). Both human genetics and observational analyses were related to prostate cancer risk, which are related to disease prevention.

### Effects of SGLT2 inhibition on prostate cancer risk

The characteristics of the primary and stringent genetic instruments used to proxy SGLT2 inhibition are listed in Tables 1, S2, S3, and S4, respectively. Across these exposures, the F-statistics used to test the relevance MR assumption suggested that weak instrument bias was unlikely to be an issue in this study (Figure S2).

**Table 1:** Characteristics of genetic variants associated with HbA_1c_ (per 0.62% lowering) or expression levels of the *SLC5A2* gene and used as proxies for SGLT2 inhibition in the general population

Genetic variant	Gene	Effect allele/non-effect allele	Effect allele frequency	Effect (95% CI)	*p* value
**SGLT2 (primary)**					

rs1232538	*SLC5A2*	G/T	0.73	−0.014 (−0.009 to −0.019)	4.0 × 10^−8^
rs28675289	*SLC5A2*	T/C	0.04	−0.038 (−0.027 to −0.049)	1.5 × 10^−11^
rs28692853	*SLC5A2*	A/C	0.50	−0.015 (−0.010 to −0.019)	2.8 × 10^−10^
rs45625038	*SLC5A2*	C/T	0.97	−0.041 (−0.028 to −0.055)	1.2 × 10^−9^
rs55766044	*SLC5A2*	C/T	0.72	−0.018 (−0.013 to −0.023)	3.9 × 10^−12^
rs557720784	*SLC5A2*	C/T	0.95	−0.026 (−0.016 to −0.037)	6.1 × 10^−7^
rs8050500	*SLC5A2*	C/T	0.45	−0.027 (−0.022 to −0.031)	1.2 × 10^−30^

**SGLT2 (stringent)**					

rs9930811	*SLC5A2*	G/A	0.37	−0.016 (−0.021 to −0.012)	8.7 × 10^−12^
rs35445454	*SLC5A2*	T/C	0.34	−0.013 (−0.018 to −0.008)	1.2 × 10^−8^

Notation: two sets of instruments proxying SGLT2 inhibition using different instrument selection processes are listed here. For the main analysis, primary instruments selected genetic variants that were robustly associated with HbA_1c_ (*p* < 1 × 10^−^^^6^^) in the *SLC5A2* region. Stringent instruments selected genetic variants that were associated with both expression of *SLC5A2* gene and HbA_1c_ levels and showed colocalization evidence between the two (colocalization probability > 0.7) in the *SLC5A2* region, which were used in the main analysis. Two pairs of primary and stringent instruments were in moderate LD (r^2^ between rs9930811 and rs8050500 = 0.56, r^2^ between rs35445454 and rs1232538 = 0.23), which suggested that the two different selection processes picked two shared genetic signals as instruments in this region.

**Figure 2:**
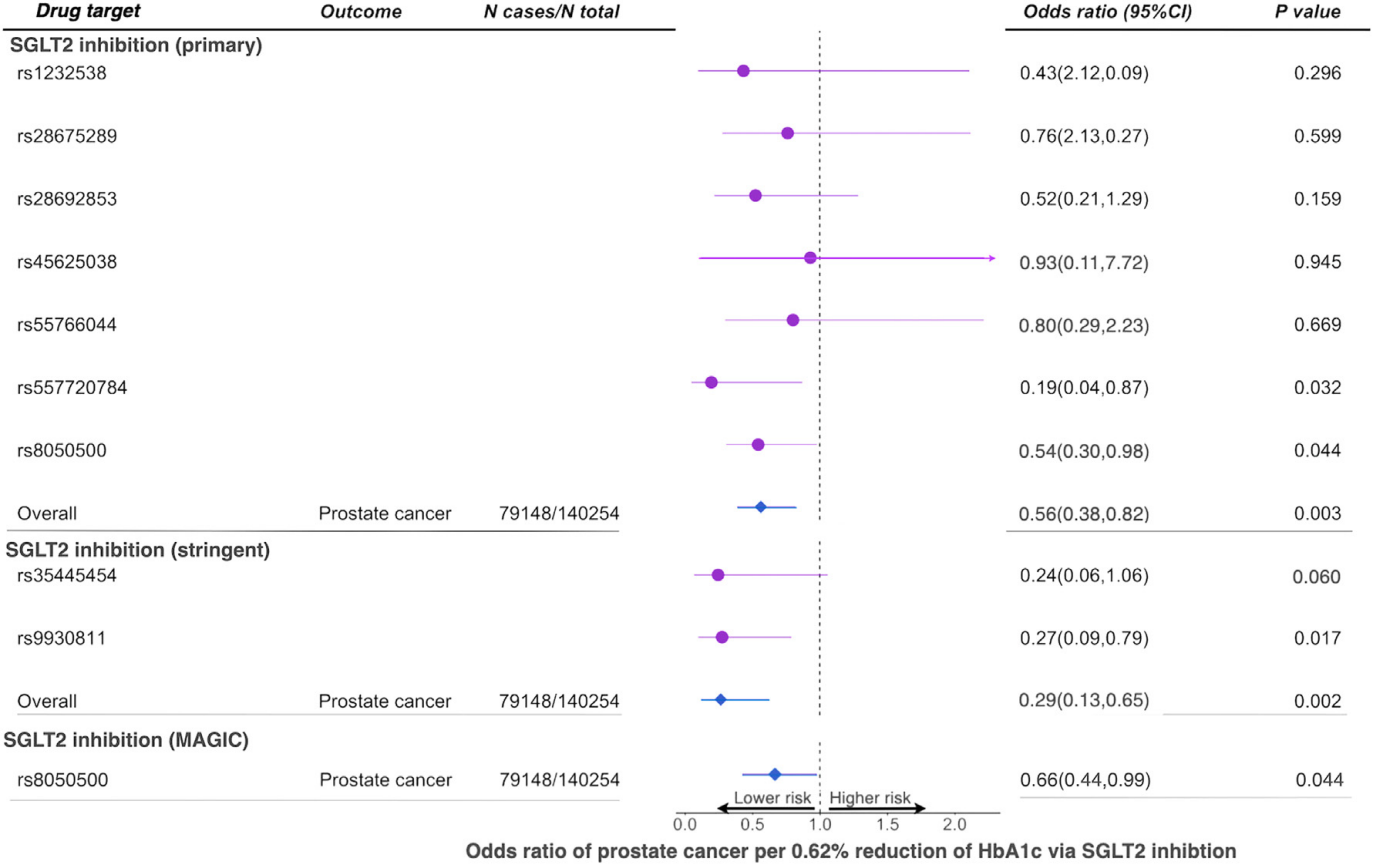
Mendelian randomization estimates of the effects of SGLT2 inhibition on prostate cancer risk in the general European population Two sets of genetic instruments were used in this analysis. Primary instruments included seven genetic variants that were associated with HbA_1c_ (*p* < 1 × 10^−^^6^) in the *SLC5A2* region. Stringent instruments were two genetic variants associated with both expression levels of *SLC5A2* and HbA_1c_ levels (with colocalization probability >0.7 between the two) in the *SLC5A2* region. Odds ratio means the reduced odds of prostate cancer risk per standard deviation unit (0.62%) reduction of HbA1c through SGLT2 inhibition.

Genetically proxied SGLT2 inhibition (estimated by primary instruments), equivalent to a one SD (0.62%) reduction in HbA_1c_, reduced the risk of total prostate cancer by 44% (odds ratio [OR] = 0.56, 95% CI = 0.38 to 0.82, *p* = 0.003; Tables 2 and S6). This effect was consistent across the seven instruments (heterogeneity *p* = 0.80; Figure 2). The other four sensitivity MR models showed similar effect estimates (Figure S3).

**Table 2:** Effect estimates of genetically proxied SGLT2 inhibition on total, aggressive, and early-onset prostate cancer among men in general population using data from the PRACTICAL and GAME-ON/ELLIPSE Consortium

Exposure	Outcome	No. of cases	Model	Odds ratio (95% CI)	*p* value
Genetically proxied SGLT2 inhibition	total prostate cancer	79,148	inverse variance weighted MR	0.56 (0.38–0.82)	0.003
	advanced prostate cancer	15,167	inverse variance weighted MR	0.52 (0.27–0.99)	0.049
	early-onset prostate cancer	6,988	inverse variance weighted MR	0.27 (0.11–0.71)	0.008
	advanced vs. non-advanced	14,160	inverse variance weighted MR	0.86 (0.35–2.13)	0.75
	high vs. low aggressive	15,561	inverse variance weighted MR	1.14 (0.38–3.39)	0.81
	high vs. low + intermediate aggressive	20,658	inverse variance weighted MR	0.69 (0.37–1.28)	0.24

Notation: advanced prostate cancer was defined as metastatic disease or Gleason score (GS) ≥ 8 or PSA > 100 or prostate cancer death; early-onset refers to prostate cancer onset before age 55; low aggressive refers to T stage from the TNM staging ≤ T1, and GS ≤ 6, and PSA < 10; intermediate aggressive refers to T stage: T2, and GS = 7, and PSA 10∼20; and high aggressive refers to T stage: T3/T4 or N1 or M1 or GS ≥ 8 or PSA > 20. Odds ratio means the reduced odds of prostate cancer risk per standard deviation unit (0.62%) reduction of HbA1c through SGLT2 inhibition.

Genetically proxied SGLT2 inhibition lowered the risk of advanced (OR = 0.52, 95% CI = 0.27 to 0.99; *p* = 0.049) and early-onset (OR = 0.27, 95% CI = 0.11 to 0.71; *p* = 0.008) prostate cancer. Little evidence was observed to support an effect of SGLT2 inhibition on other prostate-cancer-related outcomes (Table 2). In addition, there was little evidence to support an effect of SGLT2 inhibition on prostate-specific antigen (PSA) levels (β = −0.14, 95% CI = −0.30 to 0.03, *p* = 0.11; Table S6), which suggested that SGLT2 inhibition is likely to show an effect on reducing risk rather than influencing the diagnostic workup for prostate cancer. As a positive control, we confirmed the well-established effect of SGLT2 inhibition on reducing the risk of T2DM (OR = 0.66, 95% CI = 0.49 to 0.88, *p* = 0.005; Table S6).

The validation MR analysis using the two instruments selected by the stringent approach and using SGLT2 instruments derived from the MAGIC consortium validated the effect of SGLT2 inhibition on total, advanced, and advanced vs. localized prostate cancer (Figure 2; Table S7).

#### Tests of MR assumptions

The exchangeability MR assumption was tested using genetic colocalization between SGLT2 inhibition and prostate cancer (Figure S2), where we observed evidence of colocalization of the two traits in the *SLC5A2* region (colocalization probability = 72%; Table S8).

The exclusion restriction MR assumption was examined in several analyses (Figure S2). The phenome-wide association study (PheWAS) of the primary SGLT2 instruments showed that these genetic variants were associated with blood cell traits (e.g., red blood cell counts), body weight traits (e.g., waist circumference), diastolic blood pressure, and low-density lipoprotein cholesterol (Table S10). Multivariable MR adjusting for these traits, respectively (Table S10A), suggested that the effect of SGLT2 inhibition on prostate cancer was independent of these traits (Table S10B). We further tested the effect of SGLT2 inhibition on prostate cancer risk adjusted for T2DM using a multivariable MR model, and we found that the effect of SGLT2 inhibition on prostate cancer was independent of its effect on T2DM (Table S10B). In addition, the SGLT2 instruments showed associations with the expression of 17 genes excluding *SLC5A2*, with two genes being targets for existing drugs for coagulation and hemoglobinuria treatment. The 17 genes were not associated with glycemic traits or had an interaction with any anti-diabetic or anti-cancer drugs^20^ (Table S11). The differential gene expression analysis further suggested that most of the 17 genes were not associated with prostate cancer, which further reduced their probability of being pleotripy.

The MR sensitivity analyses did not provide strong evidence of heterogeneity or pleiotropy for the effect of SGLT2 inhibition on prostate cancer, but the statistical power to clearly demonstrate this was low (Tables S6 and S7).

### Association of usage of SGLT2 inhibitors with prostate cancer risk using electronic healthcare data

**Figure 3:**
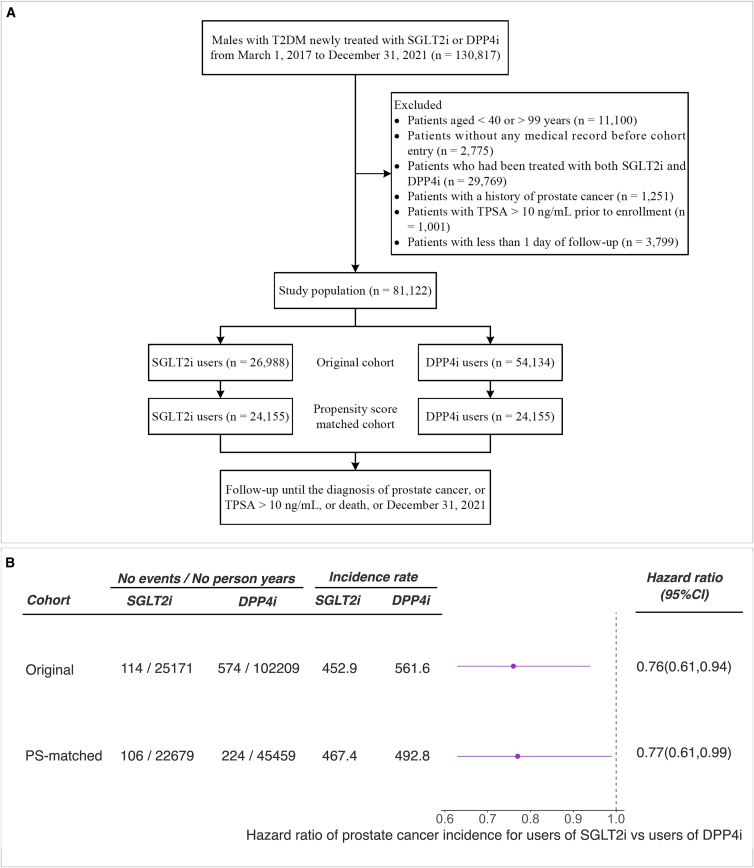
Flowchart of patient inclusion and association between the use of SGLT2 inhibitors and the risk of incident prostate cancer or being at high risk of prostate cancer (A) Flowchart of patient inclusion in the study population. SGLT2i, sodium glucose cotransporter 2 inhibitors; DPP4i, dipeptidylpeptidase 4 inhibitors; TPSA, total prostate-specific antigen. A patient could be excluded for more than one reason. (B) The association between use of SGLT2 inhibitors compared with DPP4 inhibitors and risk of prostate cancer or with total PSA > 10 ng/mL (which indicated high risk of prostate cancer). The covariates used in this analysis include demographic data (age), comorbidities (benign prostatic hyperplasia, hypertension, dyslipidemia, diabetic complications, ischemic heart disease, peripheral vascular disease, heart failure, cerebrovascular disease, chronic lung disease, moderate or severe kidney disease, moderate or severe liver disease, and other cancers), anti-diabetic drugs (metformin, insulin, glucagon-like peptide-1 receptor agonist, sulfonylurea, glinide, α-glucosidase inhibitor, and thiazolidinedione), and other medications (angiotensin-converting enzyme inhibitor, angiotensin receptor blocker, calcium channel blocker, α/β-blockers, diuretic, statin, fibrate, aspirin, other antiplatelet drugs, non-steroidal anti-inflammatory drug, and 5α-reductase inhibitor). The unit of the incidence rate was 100,000 person-years. Harzard ratio is the probability of occurrence of prostate cancer in SGLT2 inhibitor users versus that in DPP4 ihibitor users during the follow-up period.

We identified 26,988 new users of SGLT2 inhibitors and 54,134 new users of DPP4 inhibitors who fulfilled the eligibility criteria out of 130,817 males from SLHD (Figure 3A). After a 1:1 propensity score matching, we identified a cohort of 48,310 patients (24,155 in each group) with well-balanced baseline characteristics (standardized mean differences less than 1.5%) between the two treatment groups (Table S1). Cox proportional hazards model showed that SGLT2 inhibitors use (compared with DPP4 inhibitors use) was associated with a 23% reduction in the risk of prostate cancer (SGLT2 inhibitors use = 467.4 versus DPP4 inhibitors use = 492.75 per 100,000 person-years; hazard ratio [HR] = 0.77, 95% CI = 0.61 to 0.99, *p* = 0.03) during a median follow-up of 1.33 years (Figure 3B). Sensitivity analyses lagging the outcome period between one and six months showed similar protective effects, albeit less precisely estimated (Table S12).

### Validating the influence of glucose: MR and observational association of HbA_1c_ with prostate cancer

We estimated the association of HbA_1c_ with prostate cancer risk using MR and observational analyses, which aimed to investigate whether the effect of SGLT2 inhibition on prostate cancer is partly via lowering HbA_1c_ levels. Little evidence was observed to support the effect of genetically proxied HbA_1c_ on total prostate cancer risk (OR = 0.98, 95% CI = 0.92 to 1.05, *p* = 0.63; Table 3). Sensitivity MR analyses in which we removed variants within the *SLC5A2* region showed similar effects to those seen in our analyses of HbA_1c_ on prostate cancer (Table S13A). Observational analysis in the 4C study also provided little evidence to support the effect of baseline HbA_1c_ levels on incident prostate cancer after 10 years of follow-up (HR = 0.93, 95% CI = 0.80 to 1.10, *p* = 0.40); the findings barely change after excluding individuals using anti-diabetic drugs (Table 3). One additional observational analysis in 157,444 male participants from UK Biobank further confirmed the null association between HbA_1c_ and incident prostate cancer (Table S13B; Figure S4).

**Table 3:** Effect estimates of genetically proxied HbA_1c_ levels on total, aggressive, and early-onset prostate cancer among men in the general population using data from the PRACTICAL Consortium and association of observed HbA_1c_ levels on incident prostate cancer among men in the general population using data from the 4C study

Exposure	Outcome	No. of cases	Model	Odds ratio (95% CI)	Hazard ratio (95% CI)	*p* value
Genetically proxied HbA_1c_ levels	total prostate cancer	79,148	inverse variance weighted MR	0.98 (0.92–1.05)	–	0.63
	aggressive prostate cancer	15,167	inverse variance weighted MR	0.99 (0.92–1.07)	–	0.81
	early-onset prostate cancer	6,988	inverse variance weighted MR	0.94 (0.82–1.08)	–	0.37
Observed HbA_1c_ levels (one SD unit = 1.11%)	incident prostate cancer (including all 57,779 males)	223	Cox proportional hazard model	–	0.93 (0.80–1.10)	0.40
Observed HbA_1c_ levels (one SD unit = 0.91%)	incident prostate cancer (excluding users of anti-diabetic drugs)	201	Cox proportional hazard model	–	0.95 (0.80–1.12)	0.53

Notation: aggressive prostate cancer, defined as Gleason score ≥ 8, PSA > 100 ng/mL, metastatic disease (M1), or death from prostate cancer, and early-onset prostate cancer, defined as participants diagnosed with prostate cancer before the age of 55 years. SD refers to standard deviation. Odds ratio is the reduced odds of prostate cancer per standard deviation unit reduction of HbA1c levels (0.62%). Hazard ratio is the probability of occurence of prostate cancer in SGLT2 inhibitor users versus that in DPP4 inhibitor users during the follow-up period.

The existing literature primarily from individuals of European ancestry had reported a protective association between diabetes and prostate cancer, but the studies from the Chinese population appear to show less consistent results.^19^^,^^21^^,^^22^^,^^23^^,^^24^^,^^25^^,^^26^ We therefore tested the observational association of T2DM on prostate cancer in the 4C study. This analysis using the 10-year follow-up data did not show any evidence to support a protective or risk-increasing effect between the two (Table S13C). The discrepancy in the findings may be attributable to factors such as the relatively small sample size and shorter follow-up duration in the 4C study.

To further identify the potential biological mechanisms of SGLT2 inhibitors on prostate cancer, we applied MELODI Presto^27^ to identify potential mediators that can link SGLT2 inhibitors with prostate cancer. This analysis suggested that intermediated traits such as obesity, the mammalian target of rapamycin, heme oxygenase-1 (an antioxidant with anti-inflammatory properties),^28^ and insulin are potential intermediate phenotypes that may inform the non-glycemic mediators of SGLT2 inhibitors on prostate cancer (Table S14).

## Discussion

In this study, we triangulated human genetics, electronic healthcare, and prospective cohort evidence to answer the same causal question: the effect of SGLT2 inhibition on prostate cancer. In the genetic analysis, we observed that genetically proxied lifelong SGLT2 inhibition reduced total, advanced, and early-onset prostate cancer in the general male population by 44%, 48%, and 73%, respectively. Validation using various selection processes and datasets confirmed the protective effect of SGLT2 inhibition on the risk of prostate cancer and its subtypes, rather than an effect on PSA biasing the diagnosis of prostate cancer. In the validation using electronic healthcare data, we showed that SGLT2 inhibitor use reduced the risk of prostate cancer by 23% in men with T2DM. In the analyses validating the influence of glucose, we found little genetic and observational evidence to support an association of HbA_1c_ with prostate cancer, which implies a possible non-glucose mechanism of SGLT2 inhibition on prostate cancer prevention. Correctively, we provided three strands of evidence to prioritize SGLT2 inhibition as a target for prostate cancer prevention.

According to the US Centers for Disease Control and Prevention, adults aged between 45 and 64 receive the greatest number of new diagnoses of diabetes, which was also the age group that men are likely to receive diagnoses of prostate cancer. However, there was little evidence to support the setting up of clinical guidelines concerning the modification of SGLT2 inhibitor treatment among diabetic patients with co-existing or high-risk prostate cancer until now. A small number of observational studies supported the protective role of SGLT2 inhibitors on prostate cancer risk.^9^ A recent systematic review of RCTs provided weak evidence of an effect of SGLT2 inhibitors on cancers.^29^ Only one phase 1 trial was registered in ClinicalTrials.gov (NCT04887935), which aims to investigate the safety of dapagliflozin, one type of SGLT2 inhibitor, for men considered at high risk of prostate cancer. In the present study, we observed robust human genetics and electronic healthcare evidence to support the effect of SGLT2 inhibition on reducing the risk of prostate cancer, both in the general male population and in males with diabetes. Our results further support that SGLT2 inhibition may have better efficacy on the prevention of early-onset prostate cancer than on total and advanced prostate cancer. Our evidence supports the prioritization of future clinical trials of SGLT2 inhibitors in diabetic men at high risk of prostate cancer, which may have the potential to influence clinical guidelines/standards for diabetes.

It has been hypothesized that the primary mechanism of a beneficial effect of SGLT2 inhibitors on cancer is through inhibiting glycolysis in tumor cells, thus reducing tumor cell proliferation and tumorigenesis.^30^ Another study showed that canagliflozin, one type of SGLT2 inhibitor, inhibits mitochondrial complex-I and cellular proliferation in prostate cancer cells.^31^ However, the lack of MR and observational evidence of a role for HbA_1c_^32^ suggests that HbA_1c_ may not be driving the observed association of SGLT2 inhibition with prostate cancer. Correctively, our genetic evidence implies that SGLT2 inhibition may have a direct effect on prostate cancer prevention, which could be independent to its glucose control effect. Some well-designed clinical trials have also provided evidence to support that SGLT2 inhibitors have good tolerance and safety profiles to be used in individuals without diabetes.^33^ Further functional and clinical studies are warranted to better understand the anti-cancer mechanism of SGLT2 inhibitors and test their anti-prostate cancer efficacy in individuals without diabetes.

Our study has several strengths. First, we estimated the effects of SGLT2 inhibition on prostate cancer prevention using genetic, electronic healthcare, and epidemiological approaches, which have different assumptions, key source of biases (e.g., pleiotropy for MR and confounders for observational analysis),^11^ and different subgroup of population (i.e., the general male population and males with diabetes). Triangulation of evidence suggests that SGLT2 inhibition is likely to have a protective effect on prostate cancer in all subpopulation groups, which strengthens confidence in this finding. Second, the instruments for SGLT2 were selected using two widely applied pipelines.^32^ The reliability of these instruments has been tested thoroughly in this study. Third, we paid special attention to the potential influence of our genetic variant-exposure estimates on our MR results and only used male-specific instruments in this study. Fourth, the results from colocalization analysis, PheWAS, multivariable MR, and other sensitivity MR analyses suggested that the effect of SGLT2 inhibition on prostate cancer is unlikely to violate the exchangeability and the exclusion restriction assumptions of MR. More interestingly, we extended the scope of differential gene expression analysis to distinguish pleiotropy from causality, and the strategy can be widely applied to other drug target genes and complex diseases.

### Limitations of the study

This study has several limitations. First, our MR estimates of the effect of SGLT2 inhibition were scaled to represent the on-target reductions in HbA_1c_ levels rather than the direct effect of SGLT2 inhibitors. This assumes that SGLT2 inhibition has a proportional impact on lowering of HbA_1c_. Second, caution is needed to interpret the causal effect estimate from this study. This is because the MR estimate reflects the long-term modulation of drug targets on disease risk, which may suggest different levels of risk reductions per unit change in drug target compared with those observed from clinical trials/observational studies over a relatively short duration, which would explain the attenuated effect estimate of our observational analysis. Furthermore, the estimated effect of SGLT2 inhibition on prostate cancer could at least in part be influenced by different ancestries, disease status, and survival bias, given the relatively late age-at-onset of prostate cancer. Third, the MR analyses presented assume no gene-environment interaction in the association of genetic proxies for drug targets and prostate cancer. Fourth, SGLT2 inhibitors have been marketed in China since March 2017; the median follow-up time for the observational analysis was therefore only 1.33 years. Therefore, we consider this result as a validation for evidence triangulation rather than a stand-alone finding. Fifth, due to lack of data in the SLHD database, we were not able to include socioeconomic status, family history of diseases, and lifestyle factors into the regression model, which may introduce confounding and bias the results. Finally, it is important to notice that the observational analyses using electronic healthcare records were mainly conducted in East Asian participants, while the genetic analysis was conducted only using GWAS of European ancestry. Given variation in the prevalence of prostate cancer across ancestries,^34^ such ancestry disparities may influence the interpretation of the results. Therefore, we refrain from interpreting our findings as indicating that SGLT2 inhibition exhibits a protective effect on prostate cancer in both ancestries.

### Conclusion

Genetic, electronic healthcare, and epidemiological evidence with different assumptions and using different subpopulations support the role of SGLT2 inhibition in reducing prostate cancer risk. Further clinical trials should be prioritized to establish whether there is a similar effect with the long-term prescription of SGLT2 inhibitors, at what age chemoprevention/treatment would need to commence, whether high-risk men should be targeted, and the potential harms.

## STAR★Methods

### Key resources table

**Table:** 

REAGENT or RESOURCE	SOURCE	IDENTIFIER
**Deposited data**		

GWAS of HbA1c	UK Biobank	https://www.nealelab.is/uk-biobank
eQTL of *SLC5A2*	GTEX	N/A
GWAS of prostate cancer	PRACTICAL	http://practical.icr.ac.uk/
Cohort study with HbA1c and prostate cancer	The 4C study	https://www.rjh.com.cn/2018RJPortal/4c/index.shtml
Electronic healthcare data for usage of SGLT2i, DPP4i and prostate cancer events	The Shanghai Link Healthcare Database	https://pubmed.ncbi.nlm.nih.gov/37400692/

**Software and algorithms**		

MR models	Hemani et al.^35^	https://github.com/MRCIEU/TwoSampleMR
Colocalization analysis	Giambartolomei et al. ^36^	https://github.com/chr1swallace/coloc

### Resource availablility

#### Lead contact

Further information and requests for resources and reagents should be directed to and will be fulfilled by the lead contact, Jie Zheng (jie.zheng@bristol.ac.uk).

#### Materials availability

This study did not involve any other unique materials.

#### Data and code availability

The data, analytic methods, and study materials will be made available to other researchers for purposes of reproducing the results. In more details, the genetic association data of the selected risk factors are available in the supplemental tables. The summary level GWAS statistics for the primary and secondary outcomes are available from the MRC IEU OpenGWAS database: https://gwas.mrcieu.ac.uk/. UK Biobank received ethical approval from the Research Ethics Committee (REC reference for UK Biobank is 11/NW/0382). The analytical script of the MR analysis that had been used in this study is available via the GitHub repository of the TwoSampleMR R package (17). Any additional information required to reanalyze the data reported in this work paper is available from the lead contact upon request.

### Experimental model and subject details

#### The PRACTICAL and GAME-ON/ELLIPSE consortium

Genome-wide association study summary statistics were obtained from the PRACTICAL and GAME-ON/ELLIPSE consortia or Kachuri et al.^17^^,^^37^ (*n* = 140,254 men from the general population). In total, eight prostate cancer related phenotypes were selected as outcomes for this study: total-, aggressive-, early-onset-, high aggressive vs. low aggressive-, high aggressive vs. low and intermediate aggressive-, advanced stage vs. localized stage prostate cancer. Advanced prostate cancer was defined as metastatic disease or Gleason score (GS) ≥ 8 or PSA >100 or prostate cancer death; early-onset refers to prostate cancer onset before age 55; low aggressive refers to T stage from the TNM staging ≤ T1, and GS ≤ 6, and PSA<10; intermediate aggressive refers to T stage: T2, and GS = 7, and PSA 10–20; and high aggressive refers to T stage: T3/T4 or N1 or M1 or GS ≥ 8 or PSA >20. PSA levels were included as they drive prostate cancer diagnoses, and we wanted to exclude an effect of the exposures on PSA that could bias the prostate cancer associations. Detailed information of the prostate cancer related outcomes was listed in Table S5.

#### The Shanghai Link Healthcare Database

The Shanghai Link Healthcare Database (SLHD) is developed and operated by the Shanghai Hospital Development Center (SHDC),^19^ which is an administrative department of the Shanghai Municipal Government. The SHDC is responsible for the surveillance of 35 tertiary hospitals in Shanghai. In China, government-run hospitals are classified as primary (grade I), secondary (grade II), or tertiary (grade III) hospitals according to their abilities in medical care, medical education, and medical research, with tertiary hospitals being the best. According to administrative regulations, all 35 tertiary hospitals are required to upload general medical practice data (i.e., outpatient visits, emergency department visits, and hospital admissions) to the SLHD. Any personally identifiable information is scrambled to protect privacy. The SLHD has released data for academic research since 2013, which requires review and approval to access.

#### The China Cardiometabolic and Cancer Cohort (4C) study

The China Cardiometabolic and Cancer Cohort (4C) study was a multi-center, population-based, prospective cohort study aiming to demonstrate whether abnormal glucose metabolism (diabetes and prediabetes) was associated with increased risk for cancer in the Chinese population and to identify factors that modify the risk of cancer among individuals with abnormal glucose metabolism.^6^ Between 2011 and 2012, a total of 259,657 individuals aged 40 years and older were recruited from 25 communities of various regions of China. Eligible men and women aged ≥40 years were identified from local resident registration systems. Trained community health workers visited eligible individuals’ homes and invited them to participate in the study.

### Method details

#### Causal inference analyses using Mendelian randomization

##### Identification of drug target of SGLT2 and exposure data

This study investigated drug target for SGLT2 inhibitors. The drug targeted gene of for SGLT2, SLC5A2 was well defined in the literature.^38^

Three sets of genetic instruments were used to proxy effect of SGLT2 inhibition (Figure S1). For main drug target MR, summary data were obtained from a GWAS of HbA1c levels in the UK Biobank (*n* = 159,160 males), in which genetic variants associated with HbA1c in the SGLT2 region were selected as instruments. For the validation MR, a set of genetic variants associated with both HbA1c and expression levels of SGLT2 (data from the GTEX and eQTLGen consortia [*n* ≤ 31,684]^39^^,^^40^).

For independent validation MR analyses, the GWAS of HbA1c levels from the MAGIC consortium^41^ were used. The primary MAGIC GWAS was a *trans*-ancestry meta-analysis, for which we consider population structure may be a confounder to bias the MR estimates. We therefore used the European-only GWAS results from 146,806 European individuals. In addition, since the genetic effects of the MAGIC HbA1c GWAS was scaled to percentage unit in the original study. We conducted a beta transformation for the genetic effects of HbA1c. After transformation, the unit of HbA1c GWAS was changed to standard deviation (SD) decreasing unit. By applying this transformation, the MR effect estimates were comparable between UK Biobank and MAGIC. In addition, For the MAGIC GWAS, individuals with type 1 or type 2 diabetes, with usage of diabetes-relevant medications or has a fasting glucose 7 mmol L^−1^, 2-h glucose ≥11.1 mmol L^−1^ or HbA1c ≥ 6.5% were excluded from the analysis.

##### Instrument selection

As demonstrated in Figure S1, we applied three instrument selection approaches to select genetic instruments for SGLT2 inhibition from two independent datasets.

The first approach selected SGLT2 instruments from a classic drug target instrument selection process (primary instruments). The genetic variants associated with HbA1c with a region-wide association threshold of *p* < 1 × 10^−^^6^ in the SLC5A2 gene region (target gene for SGLT2 inhibition) were selected as candidate instruments. After selection, seven variants that proxying SGLT2 inhibition were selected as set 2 instruments for SGLT2 inhibition (Table S2).

The second approach selected instruments for the main drug target MR analyses (stringent instruments). Genetic variants associated with expression levels of drug target genes in a regional-wide significance threshold (*p* < 0.001) and HbA1c in a region-wide significance level (*p* < 1 × 10^−^^6^) in a genomic region near the drug target gene (±1Mb window) were selected as candidate instruments. We systematically scanned genetic variants associated with the expression levels of SLC5A2 using data from seven recent GWAS studies of genes level in 49 human tissues and proteins in plasma.^42^^,^^43^^,^^44^^,^^45^^,^^46^^,^^47^^,^^48^ This is because targets for SGLT2 inhibition may influence glycemic traits via biological mechanisms in different tissues. A set of genetic colocalization methods^36^^,^^49^ were then used to select genetic variants with shared causal variants of expression level of the drug target gene and HbA1c in the gene coding region. This step mapped 44 genetic variants for SGLT2 (Table S3). We further applied linkage disequilibrium (LD) clumping to select those with the lowest *p* value that had an LD (which refers to pairwise squared correlation [r^2^]) less than 0.15 as this indicates weak correlation among the selected genetic variants. European population specific LD among variants were estimated from the 1000 Genomes Project (phase 3) implemented in the two-sample MR package.^35^^,^^44^ After filtering, two variants were selected as instruments for SGLT2 inhibition (Table S2A).

The third approach selected instruments of SGLT2 inhibition from an independent dataset from MAGIC consortium. The genetic variants passed regional-wide association threshold of *p* < 1 × 10^−5^ in the SLC5A2 region were selected as candidate instruments. LD clumping with a threshold of 0.01 was further applied to select complete independent genetic variants as genetic instruments. After selection, one genetic variant that proxying SGLT2 inhibition were selected as instrument (Table S4).

#### Outcome selection for human genetics analysis

Eight prostate cancer related phenotypes were selected as outcomes for the MR analysis: total-, aggressive-, early-onset-, high aggressive vs. low aggressive-, high aggressive vs. low and intermediate aggressive-, advanced stage vs. localised stage prostate cancer. Detailed information of the prostate cancer related outcomes was listed in Table S5.

#### Mendelian randomization analyses

Germline genetic variants used to proxy SGLT2 inhibition were matched to prostate cancer datasets by orienting effects of the exposure and the outcome to the same effect allele. If an instrument was missing in the outcome dataset, a genetic variant with high LD (r^2^ > 0.8) to the instrument was selected as a proxy instrument where possible. An inverse-variance weighted approach was used to combine variant-level Wald ratio estimates into an overall effect estimate. All MR estimates (odds ratios [ORs]) were scaled to SD unit to reflect the equivalent of a one SD unit (0.62%) reduction in HbA_1c_.

In the main MR analyses, the effects of genetically proxied SGLT2 inhibition (using seven primary instruments) were estimated on total prostate cancer, its subtypes and PSA levels in the general male population (PRACTICAL and GAME-ON/ELLIPSE).^17^ The effect of SGLT2 inhibition on T2DM^50^ was estimated as a positive control analysis. For the validation MR analyses, the effects of SGLT2 inhibition on the prostate cancer related outcomes were estimated using the stringent instruments and instruments from the independent dataset (MAGIC).

We report findings according to the STROBE-MR (Strengthening the Reporting of Mendelian Randomization Studies) guidelines^51^^,^^52^ (the STROBE-MR check list as Data S1, related to STAR Methods). The three key MR assumptions were tested using the sensitivity methods, including generalized inverse variance weighted (gIVW),^53^ genetic colocalization,^36^^,^^49^ phenome-wide association studies (including classic risk factors associated with SGLT2 instruments) using data from the IEU OpenGWAS database,^18^ heterogeneity tests across instruments using Cochran’s Q, weighted median and mode-based estimate approaches and Multivariable MR.^54^

In more details, MR exploits both Mendel’s Law of Heredity.^55^ The Law of Independent Assortment refers to the fact that alleles of genes in different parts of the genome are inherited independently. Compliance with this Law was evaluated using a generalized inverse variance weighted (gIVW) model,^53^ which takes into account the weak LD (r^2^ = 0.089) between the SGLT2 instruments.

The MR assumption of relevance was tested by generating estimates of the proportion of variance in each drug target explained by the instrument (R2) and F statistics. An F statistic of at least 10 is indicative of evidence against weak instrument bias (a reduction in statistical power to reject the null hypothesis when an instrument explains only a small proportion of variance in an exposure).^56^

The MR assumption of exchangeability was tested by performing a genetic colocalization analysis between the drug target and prostate cancer.^36^^,^^49^ This can be used to assess whether false-positive drug target-disease associations were created due to confounding by LD between nearby genetic variants (genetic confounding). A posterior probability of colocalization over 70% between a drug target and prostate cancer was used as evidence of colocalization.

The MR assumption involving the exclusion restriction was tested using a whole set of sensitivity methods. First, the presence of an association between an instrument for SGLT2 inhibition and an off-target phenotype could provide evidence of horizontal pleiotropy (which means a genetic variant influences a phenotype through biological pathways that are independent of the exposure under investigation), which is a violation of the exclusion restriction criterion. A phenome-wide association study (PheWAS) of the genetic instruments for SGLT2 inhibition was performed among a comprehensive list of 22,479 human phenotypes included in the IEU OpenGWAS database.^18^ If there was evidence of effect of genetic instruments for SGLT2 inhibition with unintended phenotypes at a genetic association threshold of 5 × 10^−8^, multivariable analyses were performed to examine associations between the genetic instruments for SGLT2 inhibition and prostate cancer outcomes, adjusted for genetically proxied phenotype.^57^

Second, if there was evidence of genetic effect of the SGLT2 instruments on expression levels of other genes, where the expression levels of these genes were associated with prostate cancer, then this will violate the exclusion restriction assumption of MR. We therefore conducted a transcriptome wide variant lookup to identify all genes that are associated with the SGLT2 instruments with *p* < 1 × 10^−4^ (Table S11). Differential expression analysis was then applied for expression levels of these genes in prostate tumor tissue versus normal prostate tissue. If expression level did not different between the two tissues, we will be more confident that these genes are not likely to be pleiotropic exposures that linking SGLT2 instruments with prostate cancer risk.

Third, the violations of the exclusion restriction assumption were further tested by examining associations of the genetic instruments with four previously reported causal prostate cancer risk factors (accelerometer-based physical activity measurement, serum iron, body mass index and monounsaturated fatty acids).^58^ A marginal MR threshold (*p* < 0.05) was used as evidence of a potential pleitropy effect of the genetic instruments for SGLT2 inhibition on prostate cancer via a prostate cancer risk factor.

Fourth, for genetic instruments for SGLT2 inhibition with two or more SNPs, evidence of horizontal pleiotropy was examined via the following sensitivity analyses: heterogeneity test across instruments using Cochran’s Q and Rücker’s Q,^59^^,^^60^ weighted median^61^ and mode-based estimate approaches.^62^ Weighted median MR and mode estimator approaches^61^^,^^62^ are two additional sensitivity analyses, which provide consistent causal estimates of the exposure on the outcome even when up to 50% (or up to 100% for the mode estimator approach) of the information contributing to the analysis comes from genetic variants that exhibit pleiotropy (or even the majority of information in the case of the mode-based MR).

If all MR sensitivity methods provide similar causal estimates of genetic proxied SGLT2 inhibition on prostate cancer, we are more confident that the causal estimates were robust to various MR assumptions.

Moreover, the SGLT2 instruments were associated with other 17 genes. We estimated whether the 17 genes were associated with glycemic traits or to have an interaction with any anti-diabetic or anti-cancer drugs. For all MR analyses, Bonferroni corrections were applied to establish multiple testing-adjusted thresholds. All the MR analyses were conducted using the TwoSampleMR R package v0.5.6.^35^

#### Observational analysis using electronic healthcare data

The survival analysis was conducted using data from the Shanghai Link Healthcare Database (SLHD),^19^ a representative clinical database covering >99% of Shanghai residents.

Figure 3A illustrates the selection process of the study population. First, all males aged between 40 and 99 years newly treated with SGLT2 inhibitors or DPP4 inhibitors from March 1, 2017 to December 31, 2021 were identified. Cohort entry was defined as the date of the first prescription. Exclusion criteria were defined as follows: patients without any medical record before cohort entry; patients who had been treated with both SGLT2 inhibitors and DPP4 inhibitors; patients with a history of prostate cancer; patients with total prostate specific antigen (PSA) > 10 ng/mL prior to enrollment; patients with less than 1 day of follow-up. All patients were followed until diagnosis of prostate cancer or death, or December 31, 2021, whichever occurred first.

The following covariates that may affect prostate cancer risk and/or total PSA levels were adjusted in the cox model:
(1) demographic data (age),
(2) comorbidities of diabetes (benign prostatic hypertrophy, hypertension, dyslipidemia, diabetic complications, ischemic heart disease, peripheral vascular disease, heart failure, cerebrovascular diseases, chronic lung disease, moderate or severe kidney disease, moderate or severe liver disease, cancers),
(3) usage of other antidiabetic drugs (including metformin, insulin, glucagon-like peptide-1 receptor agonist, sulfonylurea, glinide, α-glucosidase inhibitor, and thiazolidinedione),
(4) and other medications (angiotensin converting enzyme inhibitor, angiotensin receptor blocker, calcium channel blocker, α/β-blockers, diuretic, statin, fibrate, aspirin, other antiplatelet drugs, non-steroidal anti-inflammatory drug, and 5α-reductase inhibitor).

These factors are built up based on existing electronic healthcare records of outpatient patients. All comorbidities and medications records were assessed by relevant medical records prior to cohort entry.

In addition to the original cohort, we also established a 1:1 propensity score matched cohort of SGLT2 inhibitors users and DPP4 inhibitors users (caliper: 0.20 standard deviation of the logit of the estimated propensity score). Standardized mean differences (SMDs) were calculated for all covariates between SGLT2 inhibitors users and DPP4 inhibitors users, with values less than 10% likely to indicate relative balance.

For the survival analysis, baseline characteristics of SGLT2 inhibitors users and DPP4 inhibitors users are presented as medians with interquartile ranges (IQRs) for continuous variables and frequencies with percentages for categorical variables. The crude incidence rate of prostate cancer-by-proxy was calculated by dividing the number of cases by the number of person-years. Cox proportional hazards models were used to estimate hazard ratios (HRs) and 95% confidence intervals (CIs) of incident prostate cancer-by-proxy, comparing SGLT2 inhibitors use with DPP4 inhibitors use. Sensitivity analyses were performed by setting different lag periods: 1-month, 2-month, 3-month, and 6-month lag period. Statistical analyses were performed using R language software (version 4.1.2).

In addition, the analysis of prostate cancer subtypes was not conducted using the electronic healthcare data in SLHD since key information such as T stage and Gleason score were not available in the electronic healthcare records.

#### Validation using prospective cohort data with over 10 years of follow-up

We estimated the association between HbA1c and incident prostate cancer during a median of 10.1 years of follow-up in the China Cardiometabolic and Cancer Cohort (4C) study.^6^^,^^63^^,^^64^^,^^65^^,^^66^ After excluding participants with prostate cancer at baseline, we included 57,779 men aged 40 years or older in the final analysis. The study was approved by the Medical Ethics Committee of Ruijin Hospital, Shanghai Jiao-Tong University. All study participants provided written informed consent.

As described previously^6^^,^^63^^,^^64^^,^^65^^,^^66^ HbA1c was determined by using high-performance liquid chromatography (VARIANT II System; Bio-Rad Laboratories) in the central laboratory located at Ruijin Hospital, Shanghai, China, which is certificated by the U.S. National Glycohemoglobin Standardization Program and passed the Laboratory Accreditation Program of the College of American Pathologists. Information on prostate cancer were collected from local death and disease registries of the National Disease Surveillance Point System and National Health Insurance System with use of the ICD 10 code “C61” in the study. Cox proportional hazards model was applied to estimate the hazard ratio of HbA1c on incident prostate cancer in the overall population (*n* = 57,779). A sensitivity analysis was performed in participants without receiving glucose-lowering therapy at baseline (*n* = 53,037). Age, body mass index, tobacco consumption, alcohol consumption, physical activity, and diet score were included as covariates in the model.

#### The prospective association of HbA1c with incident prostate cancer in UK Biobank

During revision, we were required to estimate the association of HbA1c with incident prostate cancer during the follow-up in UKB men. All people in the UK National Health Service registry aged between 40 and 69 years and living within a 25 mile radius from one of 22 study centers were invited to participate between 2006-2010.^67^ In total 503,325 adults (5.5% of the ∼9.2 million invited) were recruited into UK Biobank.^67^ Ethical approval for UKB was obtained from the North West Multi-centre Research Ethics Committee, and our study was performed under UKB application number 15825.

Prostate cancer (defined using ICD 10 code C61) together with its diagnostic date were obtained from UKB linked hospital inpatient data (field ID 41270 and 41280). HbA1c at baseline (field ID 30750) was measured via HPLC analysis on a Bio-Rad VARIANT II Turbo by UKB, and outliers with levels outside four standard deviation unit from the mean were excluded. We followed the same analysis in 4C study to adjust for participants’ age (field ID 21021), body mass index (field ID 21001), smoking status (field ID 20116), drinking status (field ID 20117), regular physical activity, and healthy diet score, all of which were measured at UKB baseline. Specially, regular physical activity was derived based on the number of at least 10-min moderate (field ID 884) and vigorous (filed ID 904) PA per week, and duration of moderate (field ID 894) and vigorous (field ID 914) PA per day.^68^ Healthy diet score was derived based on UKB food frequency questionnaire, including fruits (field ID 1309, 1319), vegetables (field ID 1289, 1299), fish (field ID 1329, 1339), processed meats (field ID 1349), unprocessed red meats (field ID 1369, 1379, 1389), whole and refined grains (field ID 1438, 1448, 1458, 1468).^68^

Cox proportional hazards model was applied to estimate the hazard ratio of HbA1c on incident prostate cancer. We restricted our analysis in 161,422 male participants of European descent, who had no missingness in the exposure, outcome and all covariates. In sensitivity analysis, we further considered competing risk in the Cox model by adding an index of death (i.e., whether participants were dead due to other diseases) as a cluster.

### Qualification and statistical analysis

Data are presented as means ± standard error of the mean (SEM). All statistical analyses were conducted using R scripts. Multiple testing correction was conducted for each of the statistical analysis. The significance between two groups was assessed using unpaired Student’s t tests. A Bonferroni corrected *p* value <0.05 was considered as a threshold for putative causal evidence.

## Supplemental information

Document S1. Figures S1–S4 and Table S1 and S3–S14

Table S2. Genetic instruments selected to proxy SGLT2 inhibition and HbA1c levels (data from UK Biobank males), related to STAR Methods

Data S1. The STROBE-MR checklist of the current study, related to STAR Methods

Document S2. Article plus supplemental information

## References

[1] International Diabetes Federation. (2021). IDF Diabetes Atlas, 10th edition. https://diabetesatlas.org/atlas/tenth-edition/.

[2] GBD 2016 Causes of Death Collaborators (2017). Global, regional, and national age-sex specific mortality for 264 causes of death, 1980-2016: a systematic analysis for the Global Burden of Disease Study 2016. Lancet.

[3] Neal B., Perkovic V., Mahaffey K.W., de Zeeuw D., Fulcher G., Erondu N., Shaw W., Law G., Desai M., Matthews D.R., CANVAS Program Collaborative Group (2017). Canagliflozin and Cardiovascular and Renal Events in Type 2 Diabetes. N. Engl. J. Med..

[4] Wiviott S.D., Raz I., Bonaca M.P., Mosenzon O., Kato E.T., Cahn A., Silverman M.G., Zelniker T.A., Kuder J.F., Murphy S.A. (2019). Dapagliflozin and Cardiovascular Outcomes in Type 2 Diabetes. N. Engl. J. Med..

[5] Buse J.B., Wexler D.J., Tsapas A., Rossing P., Mingrone G., Mathieu C., D'Alessio D.A., Davies M.J. (2020). 2019 Update to: Management of Hyperglycemia in Type 2 Diabetes, 2018. A Consensus Report by the American Diabetes Association (ADA) and the European Association for the Study of Diabetes (EASD). Diabetes Care.

[6] Lu J., He J., Xu Y., Zheng R., Zheng J., Qin G., Qin Y., Chen Y., Tang X. (2024). Association of social determinants, lifestyle, and metabolic factors with mortality in Chinese adults: A nationwide 10-year prospective cohort study. Cell Rep. Med..

[7] Sung H., Ferlay J., Siegel R.L., Laversanne M., Soerjomataram I., Jemal A., Bray F. (2021). Global Cancer Statistics 2020: GLOBOCAN Estimates of Incidence and Mortality Worldwide for 36 Cancers in 185 Countries. CA A Cancer J. Clin..

[8] Dutka M., Bobiński R., Francuz T., Garczorz W., Zimmer K., Ilczak T., Ćwiertnia M., Hajduga M.B. (2022). SGLT-2 Inhibitors in Cancer Treatment-Mechanisms of Action and Emerging New Perspectives. Cancers.

[9] Murtola T.J., Tammela T.L.J., Lahtela J., Auvinen A. (2008). Antidiabetic medication and prostate cancer risk: a population-based case-control study. Am. J. Epidemiol..

[10] Tang H., Dai Q., Shi W., Zhai S., Song Y., Han J. (2017). SGLT2 inhibitors and risk of cancer in type 2 diabetes: a systematic review and meta-analysis of randomised controlled trials. Diabetologia.

[11] Lawlor D.A., Tilling K., Davey Smith G. (2016). Triangulation in aetiological epidemiology. Int. J. Epidemiol..

[12] Holmes M.V., Richardson T.G., Ference B.A., Davies N.M., Davey Smith G. (2021). Integrating genomics with biomarkers and therapeutic targets to invigorate cardiovascular drug development. Nat. Rev. Cardiol..

[13] Soni P.D., Hartman H.E., Dess R.T., Abugharib A., Allen S.G., Feng F.Y., Zietman A.L., Jagsi R., Schipper M.J., Spratt D.E. (2019). Comparison of Population-Based Observational Studies With Randomized Trials in Oncology. J. Clin. Oncol..

[14] Smith G.D., Ebrahim S. (2003). Mendelian randomization’: can genetic epidemiology contribute to understanding environmental determinants of disease?. Int. J. Epidemiol..

[15] Sanderson E., Glymour M.M., Holmes M.V., Kang H., Morrison J., Munafò M.R., Palmer T., Schooling C.M., Wallace C., Zhao Q., Davey Smith G. (2022). Mendelian randomization. Nat. Rev. Methods Primers.

[16] Lund J.L., Richardson D.B., Stürmer T. (2015). The active comparator, new user study design in pharmacoepidemiology: historical foundations and contemporary application. Curr. Epidemiol. Rep..

[17] Schumacher F.R., Al Olama A.A., Berndt S.I., Benlloch S., Ahmed M., Saunders E.J., Dadaev T., Leongamornlert D., Anokian E., Cieza-Borrella C. (2018). Association analyses of more than 140,000 men identify 63 new prostate cancer susceptibility loci. Nat. Genet..

[18] Elsworth B., Lyon M., Alexander T., Liu Y., Matthews P., Hallett J., Bates P., Palmer T., Haberland V., Smith G.D. (2020). The MRC IEU OpenGWAS data infrastructure. bioRxiv.

[19] Qi J., He P., Yao H., Song R., Ma C., Cao M., Cui B., Ning G. (2019). Cancer risk among patients with type 2 diabetes: A real-world study in Shanghai, China. J. Diabetes.

[20] Cotto K.C., Wagner A.H., Feng Y.-Y., Kiwala S., Coffman A.C., Spies G., Wollam A., Spies N.C., Griffith O.L., Griffith M. (2018). DGIdb 3.0: a redesign and expansion of the drug-gene interaction database. Nucleic Acids Res..

[21] Bansal D., Bhansali A., Kapil G., Undela K., Tiwari P. (2013). Type 2 diabetes and risk of prostate cancer: a meta-analysis of observational studies. Prostate Cancer Prostatic Dis..

[22] Jian Gang P., Mo L., Lu Y., Runqi L., Xing Z. (2015). Diabetes mellitus and the risk of prostate cancer: an update and cumulative meta-analysis. Endocr. Res..

[23] Amadou A., Freisling H., Jenab M., Tsilidis K.K., Trichopoulou A., Boffetta P., Van Guelpen B., Mokoroa O., Wilsgaard T., Kee F. (2021). Prevalent diabetes and risk of total, colorectal, prostate and breast cancers in an ageing population: meta-analysis of individual participant data from cohorts of the CHANCES consortium. Br. J. Cancer.

[24] Peila R., Rohan T.E. (2020). Diabetes, Glycated Hemoglobin, and Risk of Cancer in the UK Biobank Study. Cancer Epidemiol. Biomarkers Prev..

[25] Zaccardi F., Ling S., Brown K., Davies M., Khunti K. (2023). Duration of Type 2 Diabetes and Incidence of Cancer: An Observational Study in England. Diabetes Care.

[26] Laurberg T., Witte D.R., Gudbjörnsdottir S., Eliasson B., Bjerg L. (2024). Diabetes-related risk factors and survival among individuals with type 2 diabetes and breast, lung, colorectal, or prostate cancer. Sci. Rep..

[27] Elsworth B., Gaunt T.R. (2021). MELODI Presto: A fast and agile tool to explore semantic triples derived from biomedical literature. Bioinformatics.

[28] Campbell N.K., Fitzgerald H.K., Dunne A. (2021). Regulation of inflammation by the antioxidant haem oxygenase 1. Nat. Rev. Immunol..

[29] Marilly E., Cottin J., Cabrera N., Cornu C., Boussageon R., Moulin P., Lega J.C., Gueyffier F., Cucherat M., Grenet G. (2022). SGLT2 inhibitors in type 2 diabetes: a systematic review and meta-analysis of cardiovascular outcome trials balancing their risks and benefits. Diabetologia.

[30] Wright E.M. (2020). SGLT2 and cancer. Pflügers Archiv.

[31] Villani L.A., Smith B.K., Marcinko K., Ford R.J., Broadfield L.A., Green A.E., Houde V.P., Muti P., Tsakiridis T., Steinberg G.R. (2016). The diabetes medication Canagliflozin reduces cancer cell proliferation by inhibiting mitochondrial complex-I supported respiration. Mol. Metabol..

[32] Au Yeung S.L., Schooling C.M. (2019). Impact of glycemic traits, type 2 diabetes and metformin use on breast and prostate cancer risk: a Mendelian randomization study. BMJ Open Diabetes Res. Care.

[33] Packer M., Anker S.D., Butler J., Filippatos G., Pocock S.J., Carson P., Januzzi J., Verma S., Tsutsui H., Brueckmann M. (2020). Cardiovascular and Renal Outcomes with Empagliflozin in Heart Failure. N. Engl. J. Med..

[34] Rawla P. (2019). Epidemiology of Prostate Cancer. World J. Oncol..

[35] Hemani G., Zheng J., Elsworth B., Wade K.H., Haberland V., Baird D., Laurin C., Burgess S., Bowden J., Langdon R. (2018). The MR-Base platform supports systematic causal inference across the human phenome. Elife.

[36] Giambartolomei C., Vukcevic D., Schadt E.E., Franke L., Hingorani A.D., Wallace C., Plagnol V. (2014). Bayesian test for colocalisation between pairs of genetic association studies using summary statistics. PLoS Genet..

[37] Kachuri L., Hoffmann T.J., Jiang Y., Berndt S.I., Shelley J.P., Schaffer K., Machiela M.J., Freedman N.D., Huang W.Y., Li S.A., Easterlin R. (2022). Leveraging genetic determinants of prostate-specific antigen levels towards improving prostate cancer screening. medRxiv.

[38] Saisho Y. (2020). SGLT2 Inhibitors: the Star in the Treatment of Type 2 Diabetes?. Diseases.

[39] Consortium G.T.E. (2020). The GTEx Consortium atlas of genetic regulatory effects across human tissues. Science.

[40] Võsa U., Claringbould A., Westra H.-J., Bonder M.J., Deelen P., Zeng B., Kirsten H., Saha A., Kreuzhuber R., Yazar S. (2021). Large-scale cis- and trans-eQTL analyses identify thousands of genetic loci and polygenic scores that regulate blood gene expression. Nat. Genet..

[41] Chen J., Spracklen C.N., Marenne G., Varshney A., Corbin L.J., Luan J., Willems S.M., Wu Y., Zhang X., Horikoshi M. (2021). The trans-ancestral genomic architecture of glycemic traits. Nat. Genet..

[42] Sun B.B., Maranville J.C., Peters J.E., Stacey D., Staley J.R., Blackshaw J., Burgess S., Jiang T., Paige E., Surendran P. (2018). Genomic atlas of the human plasma proteome. Nature.

[43] Folkersen L., Fauman E., Sabater-Lleal M., Strawbridge R.J., Frånberg M., Sennblad B., Baldassarre D., Veglia F., Humphries S.E., Rauramaa R. (2017). Mapping of 79 loci for 83 plasma protein biomarkers in cardiovascular disease. PLoS Genet..

[44] Suhre K., Arnold M., Bhagwat A.M., Cotton R.J., Engelke R., Raffler J., Sarwath H., Thareja G., Wahl A., DeLisle R.K. (2017). Connecting genetic risk to disease end points through the human blood plasma proteome. Nat. Commun..

[45] Yao C., Chen G., Song C., Keefe J., Mendelson M., Huan T., Sun B.B., Laser A., Maranville J.C., Wu H. (2018). Genome-wide mapping of plasma protein QTLs identifies putatively causal genes and pathways for cardiovascular disease. Nat. Commun..

[46] Emilsson V., Ilkov M., Lamb J.R., Finkel N., Gudmundsson E.F., Pitts R., Hoover H., Gudmundsdottir V., Horman S.R., Aspelund T. (2018). Co-regulatory networks of human serum proteins link genetics to disease. Science.

[47] Võsa U., Claringbould A., Westra H.-J., Bonder M.J., Deelen P., Zeng B., Kirsten H., Saha A., Kreuzhuber R., Yazar S. (2021). Large-scale cis- and trans-eQTL analyses identify thousands of genetic loci and polygenic scores that regulate blood gene expression. Nat. Genet..

[48] Aguet F., Barbeira A.N., Bonazzola R., Brown A., Castel S.E., Jo B., Kasela S., Kim-Hellmuth S., Liang Y., Oliva M. (2019). The GTEx Consortium atlas of genetic regulatory effects across human tissues. bioRxiv.

[49] Zheng J., Haberland V., Baird D., Walker V., Haycock P.C., Hurle M.R., Gutteridge A., Erola P., Liu Y., Luo S. (2020). Phenome-wide Mendelian randomization mapping the influence of the plasma proteome on complex diseases. Nat. Genet..

[50] Mahajan A., Taliun D., Thurner M., Robertson N.R., Torres J.M., Rayner N.W., Payne A.J., Steinthorsdottir V., Scott R.A., Grarup N. (2018). Fine-mapping type 2 diabetes loci to single-variant resolution using high-density imputation and islet-specific epigenome maps. Nat. Genet..

[51] Skrivankova V.W., Richmond R.C., Woolf B.A.R., Yarmolinsky J., Davies N.M., Swanson S.A., VanderWeele T.J., Higgins J.P.T., Timpson N.J., Dimou N. (2021). Strengthening the Reporting of Observational Studies in Epidemiology Using Mendelian Randomization: The STROBE-MR Statement. JAMA.

[52] Skrivankova V.W., Richmond R.C., Woolf B.A.R., Davies N.M., Swanson S.A., VanderWeele T.J., Timpson N.J., Higgins J.P.T., Dimou N., Langenberg C. (2021). Strengthening the reporting of observational studies in epidemiology using mendelian randomisation (STROBE-MR): explanation and elaboration. BMJ.

[53] Burgess S., Zuber V., Valdes-Marquez E., Sun B.B., Hopewell J.C. (2017). Mendelian randomization with fine-mapped genetic data: Choosing from large numbers of correlated instrumental variables. Genet. Epidemiol..

[54] Sanderson E., Davey Smith G., Windmeijer F., Bowden J. (2019). An examination of multivariable Mendelian randomization in the single-sample and two-sample summary data settings. Int. J. Epidemiol..

[55] Davey Smith G., Holmes M.V., Davies N.M., Ebrahim S. (2020). Mendel’s laws, Mendelian randomization and causal inference in observational data: substantive and nomenclatural issues. Eur. J. Epidemiol..

[56] Burgess S., Thompson S.G., CRP CHD Genetics Collaboration (2011). CRP CHD Genetics Collaboration. Avoiding bias from weak instruments in Mendelian randomization studies. Int. J. Epidemiol..

[57] Burgess S., Thompson S.G. (2015). Multivariable Mendelian randomization: the use of pleiotropic genetic variants to estimate causal effects. Am. J. Epidemiol..

[58] Kazmi N., Haycock P., Tsilidis K., Lynch B.M., Truong T., Martin R.M., Lewis S.J., PRACTICAL Consortium, CRUK, BPC3, CAPS, PEGASUS (2020). Appraising causal relationships of dietary, nutritional and physical-activity exposures with overall and aggressive prostate cancer: two-sample Mendelian-randomization study based on 79 148 prostate-cancer cases and 61 106 controls. Int. J. Epidemiol..

[59] Bowden J., Del Greco M F., Minelli C., Davey Smith G., Sheehan N., Thompson J. (2017). A framework for the investigation of pleiotropy in two-sample summary data Mendelian randomization. Stat. Med..

[60] Bowden J., Del Greco M.F., Minelli C., Zhao Q., Lawlor D.A., Sheehan N.A., Thompson J., Davey Smith G. (2019). Improving the accuracy of two-sample summary data Mendelian randomization: moving beyond the NOME assumption. Int. J. Epidemiol..

[61] Bowden J., Davey Smith G., Haycock P.C., Burgess S. (2016). Consistent Estimation in Mendelian Randomization with Some Invalid Instruments Using a Weighted Median Estimator. Genet. Epidemiol..

[62] Hartwig F.P., Davey Smith G., Bowden J. (2017). Robust inference in summary data Mendelian randomization via the zero modal pleiotropy assumption. Int. J. Epidemiol..

[63] Lu J., He J., Li M., Tang X., Hu R., Shi L., Su Q., Peng K., Xu M., Xu Y. (2019). Predictive Value of Fasting Glucose, Postload Glucose, and Hemoglobin A1c on Risk of Diabetes and Complications in Chinese Adults. Diabetes Care.

[64] Lu J., Wang S., Li M., Gao Z., Xu Y., Zhao X., Hu C., Zhang Y., Liu R., Hu R. (2021). Association of Serum Bile Acids Profile and Pathway Dysregulation With the Risk of Developing Diabetes Among Normoglycemic Chinese Adults: Findings From the 4C Study. Diabetes Care.

[65] Lu J., Li M., Xu Y., Bi Y., Qin Y., Li Q., Wang T., Hu R., Shi L., Su Q. (2020). Early Life Famine Exposure, Ideal Cardiovascular Health Metrics, and Risk of Incident Diabetes: Findings From the 4C Study. Diabetes Care.

[66] Wang T.G., Lu J.L., Shi L.X., Chen G., Xu M., Xu Y., Su Q., Mu Y.M., Chen L.L., Hu R.Y. (2020). Association of insulin resistance and beta-cell dysfunction with incident diabetes among adults in China: a nationwide, population-based, prospective cohort study. Lancet Diabetes Endocrinol..

[67] Sudlow C., Gallacher J., Allen N., Beral V., Burton P., Danesh J., Downey P., Elliott P., Green J., Landray M. (2015). UK biobank: an open access resource for identifying the causes of a wide range of complex diseases of middle and old age. PLoS Med..

[68] Lourida I., Hannon E., Littlejohns T.J., Langa K.M., Hyppönen E., Kuzma E., Llewellyn D.J. (2019). Association of Lifestyle and Genetic Risk With Incidence of Dementia. JAMA.

