# Supplementary material for: The effect of SGLT2 inhibition on prostate cancer: Mendelian randomization and observational analysis using electronic healthcare and cohort data
Source: Cell Rep Med. 2024 Aug 20;5(8):101688. doi: 10.1016/j.xcrm.2024.101688 (PMC11384955; doi:10.1016/j.xcrm.2024.101688)
Supplement: Document S1. Figures S1–S4 and Table S1 and S3–S14 [file mmc1.pdf]

Cell Reports Medicine, Volume *5*

Supplemental information

The effect of SGLT2 inhibition on prostate cancer:

Mendelian randomization and observational analysis

using electronic healthcare and cohort data

Jie Zheng, Jieli Lu, Jiying Qi, Qian Yang, Huiling Zhao, Haoyu Liu, Zhihe Chen, Lanhui Huang, Youqiong Ye, Min Xu, Yu Xu, Tiange Wang, Mian Li, Zhiyun Zhao, Ruizhi Zheng, Shuangyuan Wang, Hong Lin, Chunyan Hu, Celine Sze Ling Chui, Shiu Lun Au Yeung, Shan Luo, Olympia Dimopoulou, Padraig Dixon, Sean Harrison, Yi Liu, Jamie Robinson, James Yarmolinsky, Philip Haycock, Jinqiu Yuan, Sarah Lewis, Zhongshang Yuan, Tom R. Gaunt, George Davey Smith, Guang Ning, Richard M. Martin, Bin Cui, Weiqing Wang, and Yufang Bi

Supplementary documents

Supplementary Figure 1. Four sets of instruments been used in the Mendelian randomization analysis. Related to STAR Methods

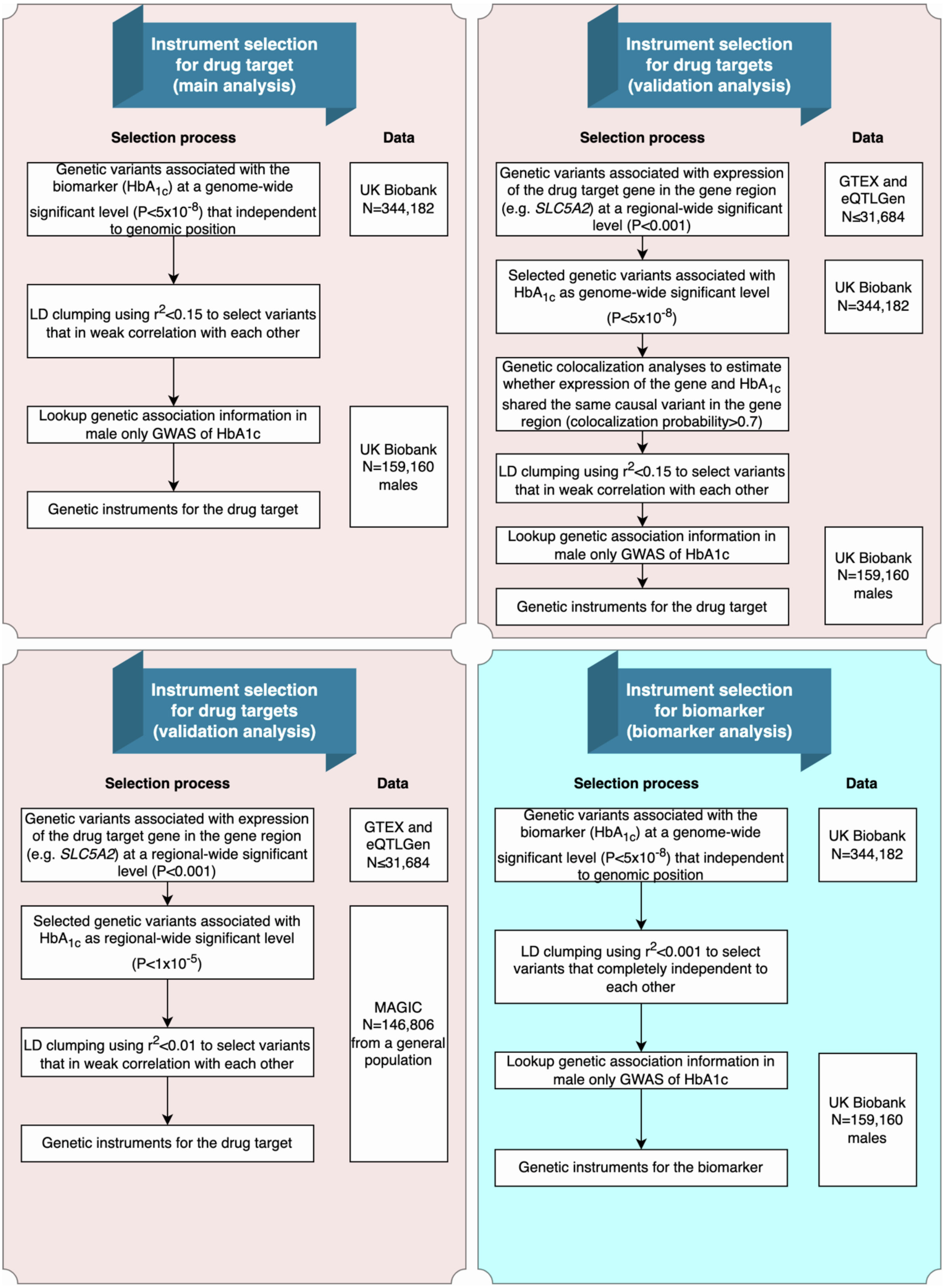

Supplementary Figure 2. Mendelian randomization assumptions. Related to STAR Methods

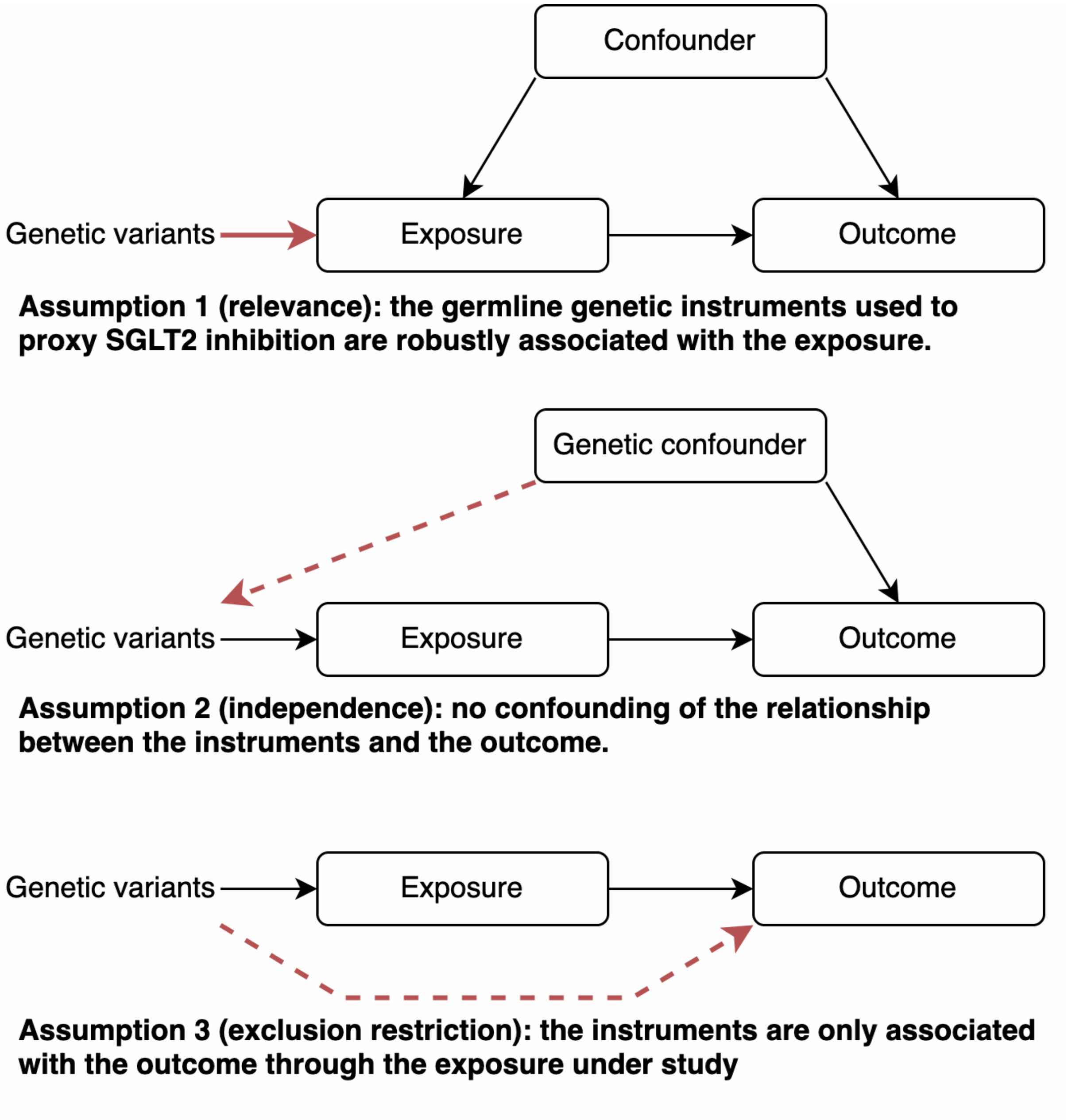

Supplementary Figure 3. Scatter plot and forest plot for the genetically proxied effect of SGLT2 inhibition on total prostate cancer. Related to STAR Methods

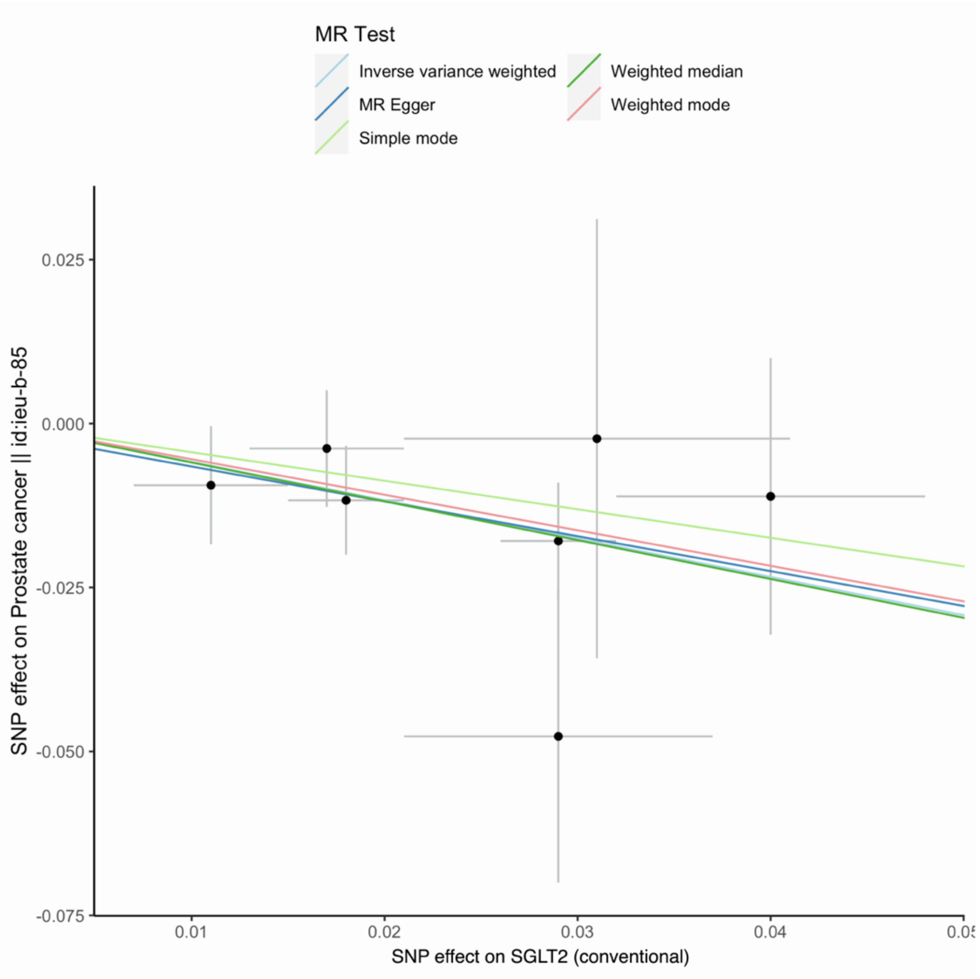

g

Supplementary Figure 4. Summary of key findings of the current study. Related to STAR Methods

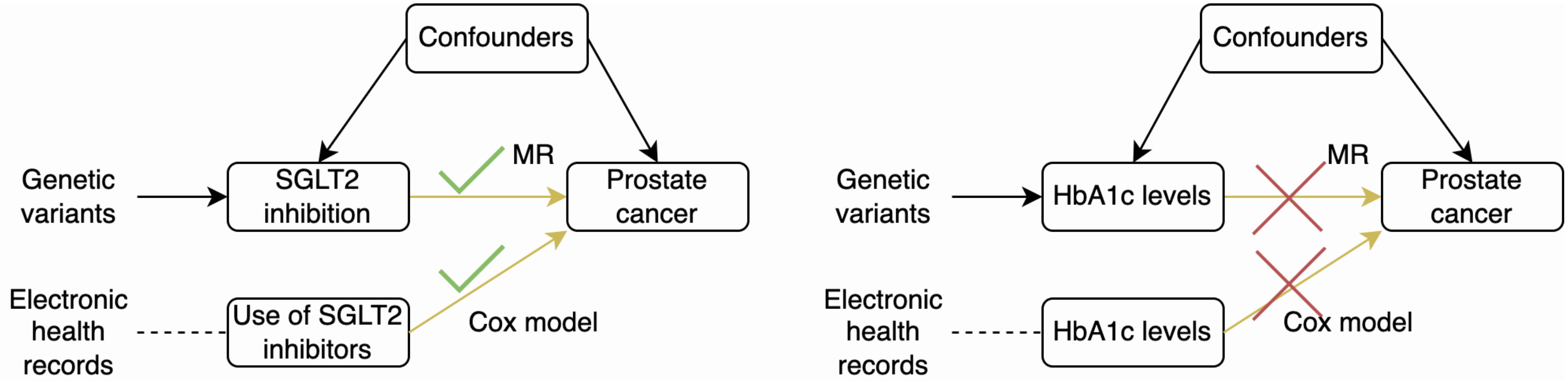

Supplementary Table 1. Baseline characteristics of users of sodium glucose cotransporter 2 (SGLT2) inhibitors and DPP4 inhibitors, before and after propensity score matching (for the survival analysis). Related to STAR Methods

	Original cohort			PS-matched cohort		
	SGLT2i (n = 26,988)	DPP4i (n = 54,134)	SMD	SGLT2i (n = 24,155)	DPP4i (n = 24,155)	SMD
Age, median (IQR)	62 (54-69)	63 (56-70)	14.2%	63 (55-69)	62 (55-69)	0.6%
Comorbidities, N (%)						
BPH	3,485 (12.91)	7,665 (14.16)	3.6%	3,138 (12.99)	3,256 (13.48)	1.4%
Hypertension	15,286 (56.64)	24,535 (45.32)	22.8%	13,456 (55.71)	13,581 (56.22)	1.0%
Dyslipidemia	6,824 (25.29)	10,386 (19.19)	14.7%	5,842 (24.19)	5,871 (24.31)	0.3%
Diabetic complications	5,920 (21.94)	9,703 (17.92)	10.1%	4,923 (20.38)	5,007 (20.73)	0.9%
Ischemic heart disease	9,364 (34.70)	12,104 (22.36)	27.6%	8,040 (33.29)	8,134 (33.67)	0.8%
Peripheral vascular disease	1,402 (5.19)	2,178 (4.02)	5.6%	1,242 (5.14)	1,251 (5.18)	0.2%
Heart failure	2,205 (8.17)	1,963 (3.63)	19.4%	1,661 (6.88)	1,662 (6.88)	<0.001
Cerebrovascular diseases	5,811 (21.53)	11,400 (21.06)	1.2%	5,247 (21.72)	5,316 (22.01)	0.7%
Chronic lung disease	3,750 (13.90)	7,335 (13.55)	1.0%	3,387 (14.02)	3,424 (14.18)	0.4%
Moderate or severe kidney disease	1,169 (4.33)	2,152 (3.98)	1.8%	1,061 (4.39)	1,100 (4.55)	0.8%
Moderate or severe liver disease	754 (2.79)	1,212 (2.24)	3.5%	650 (2.69)	658 (2.72)	0.2%
Other cancers	1,750 (6.48)	3,846 (7.10)	2.5%	1,607 (6.65)	1,625 (6.73)	0.3%
Antidiabetic drugs, N (%)						
Metformin	17,420 (64.55)	30,827 (56.95)	15.6%	15,199 (62.92)	15,320 (63.42)	1.0%
Insulin	12,191 (45.17)	22,753 (42.03)	6.3%	10,506 (43.49)	10,547 (43.66)	0.3%
GLP1RA	2,862 (10.60)	591 (1.09)	41.4%	625 (2.59)	591 (2.45)	0.9%
Sulfonylurea	8,573 (31.77)	17,515 (32.35)	1.3%	7,773 (32.18)	7,897 (32.69)	1.1%
Glinide	3,160 (11.71)	7,514 (13.88)	6.5%	2,859 (11.84)	2,908 (12.04)	0.6%
α-glucosidase inhibitor	9,958 (36.90)	21,510 (39.73)	5.8%	8,901 (36.85)	8,975 (37.16)	0.6%
Thiazolidinedione	5,594 (20.73)	8,222 (15.19)	14.5%	4,795 (19.85)	4,931 (20.41)	1.4%
Medications, N (%)						
ACEI	4,997 (18.52)	6,813 (12.59)	16.4%	4,283 (17.73)	4,294 (17.78)	0.1%
ARB	13,570 (50.28)	20,426 (37.73)	25.5%	11,736 (48.59)	11,790 (48.81)	0.4%
CCB	13,284 (49.22)	22,182 (40.98)	16.6%	11,665 (48.29)	11,797 (48.84)	1.1%
α/β-blockers	12,237 (45.34)	17,822 (32.92)	25.7%	10,594 (43.86)	10,693 (44.27)	0.8%
Diuretic	9,194 (34.07)	14,256 (26.33)	16.9%	7,971 (33.00)	7,984 (33.05)	0.1%
Statin	15,778 (58.46)	25,215 (46.58)	24.0%	13,705 (56.74)	13,852 (57.35)	1.2%
Fibrate	2,756 (10.21)	3,869 (7.15)	10.9%	2,284 (9.46)	2,283 (9.45)	<0.001
Aspirin	12,394 (45.92)	19,275 (35.61)	21.1%	10,777 (44.62)	10,917 (45.20)	1.2%
Other antiplatelet drugs	9,238 (34.23)	12,254 (22.64)	25.9%	7,985 (33.06)	8,015 (33.18)	0.3%
NSAID	10,399 (38.53)	19,863 (36.69)	3.8%	9,241 (38.26)	9,355 (38.73)	1.0%
5α-reductase inhibitor	1,362 (5.05)	3,458 (6.39)	5.8%	1,266 (5.24)	1,314 (5.44)	0.9%

Notes: PS-matched cohort, propensity score matched cohort; SGLT2i, sodium-glucose cotransporter-2 inhibitor; SMD, standardized mean difference.

Supplementary Table 3. Selection of genetic instruments that proxying SGLT2. Related to STAR Methods

Genetic association inforomation of the expression level of the genes												MR and colocalization of *SLC5A2* expression on HbA1c						
**Tissue**	**Gene.name**	**ENSG.ID**	**Phenotype**	**Variant_ID**	**Effect_al**	**Other_a**	**Effect_all**	**Beta**	**Se**	**P**	**N**	**beta.mr**	**se.mr**	**pval.mr**	**pass.mr**	**HbA1c-signal-in-region**	**LD-r2**	**pass.LD.check**
Lung	*SLC5A2*	ENSG00000140675.12	Lung_SLC5A2_rs9934336	rs9934336	A	G	0.252	0.522	0.045	2.85E-27	515	-0.021	0.005	1.89E-05	TRUE	rs11150626_C_T	99%	TRUE
Stomach	*SLC5A2*	ENSG00000140675.12	Stomach_SLC5A2_rs34497199	rs34497199	T	C	0.415	0.556	0.070	6.79E-14	324	-0.021	0.004	5.98E-07	TRUE	rs35856922_A_G	99%	TRUE
Artery_Tibial	*SLC5A2*	ENSG00000140675.12	Artery_Tibial_SLC5A2_rs11646054	rs11646054	C	G	0.414	-0.368	0.048	9.13E-14	584	-0.006	0.006	3.09E-01	FALSE	NA	NA	FALSE
Pancreas	*SLC5A2*	ENSG00000140675.12	Pancreas_SLC5A2_rs9934336	rs9934336	A	G	0.267	0.566	0.074	4.24E-13	305	-0.021	0.005	1.89E-05	TRUE	rs11150626_C_T	99%	TRUE
Colon_Sigmoid	*SLC5A2*	ENSG00000140675.12	Colon_Sigmoid_SLC5A2_rs11150624	rs11150624	T	C	0.442	-0.427	0.060	1.23E-11	318	-0.005	0.005	3.69E-01	FALSE	NA	NA	FALSE
Colon_Transverse	*SLC5A2*	ENSG00000140675.12	Colon_Transverse_SLC5A2_rs11865835	rs11865835	C	T	0.315	0.435	0.066	1.60E-10	368	-0.026	0.006	1.34E-05	TRUE	rs6565235_T_C	99%	TRUE
Whole_blood	*SLC5A2*	ENSG00000140675	Whole_blood_SLC5A2_rs6565236	rs6565236	T	A	0.249	0.084	0.014	9.86E-10	31191	-0.129	0.030	2.27E-05	TRUE	rs3934739_T_C	100%	TRUE
Brain_Cerebellum	*SLC5A2*	ENSG00000140675.12	Brain_Cerebellum_SLC5A2_rs8057029	rs8057029	C	A	0.699	-0.420	0.077	1.80E-07	209	0.019	0.006	1.29E-03	TRUE	rs28692853_A_C	48%	FALSE
Brain_Frontal_Cortex_BA9	*SLC5A2*	ENSG00000140675.12	Brain_Frontal_Cortex_BA9_SLC5A2_rs11150606	rs11150606	C	T	0.011	-2.259	0.491	9.51E-06	175	-0.001	0.004	8.77E-01	FALSE	NA	NA	FALSE
Artery_Aorta	*SLC5A2*	ENSG00000140675.12	Artery_Aorta_SLC5A2_rs140791727	rs140791727	T	C	0.008	1.661	0.371	1.04E-05	387	-0.012	0.008	1.05E-01	FALSE	NA	NA	FALSE
Esophagus_Gastroesophageal_Junction	*SLC5A2*	ENSG00000140675.12	Esophagus_Gastroesophageal_Junction_SLC5A2_rs11643752	rs11643752	A	G	0.414	-0.261	0.059	1.52E-05	330	0.003	0.009	7.12E-01	FALSE	NA	NA	FALSE
Brain_Cortex	*SLC5A2*	ENSG00000140675.12	Brain_Cortex_SLC5A2_rs12448775	rs12448775	T	C	0.044	0.996	0.228	2.26E-05	205	0.019	0.006	1.68E-03	TRUE	rs11644104_C_T	6%	FALSE
Brain_Cerebellar_Hemisphere	*SLC5A2*	ENSG00000140675.12	Brain_Cerebellar_Hemisphere_SLC5A2_rs8054784	rs8054784	C	T	0.583	-0.332	0.078	3.45E-05	175	0.005	0.007	4.92E-01	FALSE	NA	NA	FALSE
Prostate	*SLC5A2*	ENSG00000140675.12	Prostate_SLC5A2_rs4561482	rs4561482	G	A	0.643	0.350	0.083	4.03E-05	221	-0.011	0.007	9.38E-02	FALSE	NA	NA	FALSE
Brain_Hypothalamus	*SLC5A2*	ENSG00000140675.12	Brain_Hypothalamus_SLC5A2_rs138759654	rs138759654	A	C	0.012	1.477	0.352	5.08E-05	170	-0.009	0.007	1.96E-01	FALSE	NA	NA	FALSE
Skin_Not_Sun_Exposed_Suprapubic	*SLC5A2*	ENSG00000140675.12	Skin_Not_Sun_Exposed_Suprapubic_SLC5A2_rs74015509	rs74015509	C	T	0.017	0.955	0.240	7.86E-05	517	NA	NA	NA	FALSE	NA	NA	FALSE
Adipose_Subcutaneous	*SLC5A2*	ENSG00000140675.12	Adipose_Subcutaneous_SLC5A2_rs3841244	rs3841244	G	GT	0.528	0.211	0.053	8.93E-05	581	NA	NA	NA	FALSE	NA	NA	FALSE
Brain_Spinal_cord_cervical_c-1	*SLC5A2*	ENSG00000140675.12	Brain_Spinal_cord_cervical_c-1_SLC5A2_rs2073918	rs2073918	G	C	0.119	0.737	0.185	1.33E-04	126	-0.003	0.006	6.19E-01	FALSE	NA	NA	FALSE
Skin_Sun_Exposed_Lower_leg	*SLC5A2*	ENSG00000140675.12	Skin_Sun_Exposed_Lower_leg_SLC5A2_rs8058958	rs8058958	A	G	0.014	0.815	0.212	1.35E-04	605	NA	NA	NA	FALSE	NA	NA	FALSE
Uterus	*SLC5A2*	ENSG00000140675.12	Uterus_SLC5A2_rs35445454	rs35445454	T	C	0.275	0.430	0.109	1.00E-04	129	-0.030	0.006	1.24E-07	TRUE	rs113138456_G_A	81%	TRUE
Ovary	*SLC5A2*	ENSG00000140675.12	Ovary_SLC5A2_rs779234311	rs779234311	TGGGGG	T	0.018	1.347	0.353	2.08E-04	167	NA	NA	NA	FALSE	NA	NA	FALSE
Spleen	*SLC5A2*	ENSG00000140675.12	Spleen_SLC5A2_rs8044603	rs8044603	G	A	0.313	0.379	0.102	2.71E-04	227	0.009	0.007	2.24E-01	FALSE	NA	NA	FALSE
Adipose_Visceral_Omentum	*SLC5A2*	ENSG00000140675.12	Adipose_Visceral_Omentum_SLC5A2_rs4488457	rs4488457	G	T	0.682	0.239	0.065	2.72E-04	469	-0.055	0.011	2.90E-07	TRUE	rs7205195_A_G	100%	TRUE
Breast_Mammary_Tissue	*SLC5A2*	ENSG00000140675.12	Breast_Mammary_Tissue_SLC5A2_rs144959592	rs144959592	G	A	0.016	-0.780	0.219	4.19E-04	396	0.016	0.012	1.82E-01	FALSE	NA	NA	FALSE
Esophagus_Mucosa	*SLC5A2*	ENSG00000140675.12	Esophagus_Mucosa_SLC5A2_rs72796569	rs72796569	T	C	0.044	-0.436	0.126	6.12E-04	497	0.018	0.012	1.51E-01	FALSE	NA	NA	FALSE
Esophagus_Muscularis	*SLC5A2*	ENSG00000140675.12	Esophagus_Muscularis_SLC5A2_rs34235897	rs34235897	C	CT	0.123	-0.270	0.078	6.41E-04	465	NA	NA	NA	FALSE	NA	NA	FALSE
Thyroid	*SLC5A2*	ENSG00000140675.12	Thyroid_SLC5A2_rs78641771	rs78641771	T	C	0.019	-0.462	0.137	7.63E-04	574	-0.005	0.014	7.33E-01	FALSE	NA	NA	FALSE
Brain_Caudate_basal_ganglia	*SLC5A2*	ENSG00000140675.12	Brain_Caudate_basal_ganglia_SLC5A2_rs529028063	rs529028063	CT	C	0.013	1.571	0.461	8.40E-04	194	0.005	0.007	4.41E-01	FALSE	NA	NA	FALSE
Brain_Hippocampus	*SLC5A2*	ENSG00000140675.12	Brain_Hippocampus_SLC5A2_rs116872173	rs116872173	C	T	0.021	1.118	0.330	9.54E-04	165	-0.034	0.011	1.37E-03	TRUE	rs4889630_T_C	4%	FALSE
Brain_Nucleus_accumbens_basal_ganglia	*SLC5A2*	ENSG00000140675.12	Brain_Nucleus_accumbens_basal_ganglia_SLC5A2_rs117219760	rs117219760	G	T	0.050	0.711	0.212	1.00E-03	202	-0.012	0.012	2.87E-01	FALSE	NA	NA	FALSE
Brain_Substantia_nigra	*SLC5A2*	ENSG00000140675.12	Brain_Substantia_nigra_SLC5A2_rs77088799	rs77088799	T	A	0.022	-1.223	0.362	1.09E-03	114	-0.002	0.008	7.85E-01	FALSE	NA	NA	FALSE
Artery_Coronary	*SLC5A2*	ENSG00000140675.12	Artery_Coronary_SLC5A2_rs8057326	rs8057326	C	T	0.484	0.267	0.082	1.42E-03	213	-0.031	0.009	2.80E-04	TRUE	rs34742827_T_C	79%	TRUE
Whole_Blood	*SLC5A2*	ENSG00000140675.12	Whole_Blood_SLC5A2_rs41476751	rs41476751	C	T	0.216	-0.183	0.058	1.57E-03	670	0.008	0.018	6.56E-01	FALSE	NA	NA	FALSE
Nerve_Tibial	*SLC5A2*	ENSG00000140675.12	Nerve_Tibial_SLC5A2_rs9930811	rs9930811	G	A	0.388	0.188	0.060	1.80E-03	532	-0.087	0.013	8.68E-12	TRUE	rs7199585_C_T	100%	TRUE
Brain_Amygdala	*SLC5A2*	ENSG00000140675.12	Brain_Amygdala_SLC5A2_rs865900824	rs865900824	C	A	0.012	-1.743	0.549	1.97E-03	129	NA	NA	NA	FALSE	NA	NA	FALSE
Liver	*SLC5A2*	ENSG00000140675.12	Liver_SLC5A2_rs142133957	rs142133957	A	G	0.007	1.557	0.502	2.26E-03	208	0.010	0.006	7.10E-02	FALSE	NA	NA	FALSE
Minor_Salivary_Gland	*SLC5A2*	ENSG00000140675.12	Minor_Salivary_Gland_SLC5A2_rs11642535	rs11642535	A	C	0.097	0.530	0.174	2.86E-03	144	-0.018	0.007	5.35E-03	TRUE	rs9933843_C_T	16%	FALSE
Brain_Putamen_basal_ganglia	*SLC5A2*	ENSG00000140675.12	Brain_Putamen_basal_ganglia_SLC5A2_rs71374021	rs71374021	GCTC	G	0.559	-0.355	0.118	3.13E-03	170	-0.047	0.007	4.91E-13	TRUE	NA	NA	FALSE
Vagina	*SLC5A2*	ENSG00000140675.12	Vagina_SLC5A2_rs72785569	rs72785569	T	C	0.082	-0.531	0.176	3.24E-03	141	-0.017	0.006	6.46E-03	TRUE	rs11574632_T_C	46%	FALSE
Pituitary	*SLC5A2*	ENSG00000140675.12	Pituitary_SLC5A2_rs118009866	rs118009866	G	A	0.023	-0.659	0.223	3.47E-03	237	0.000	0.010	9.86E-01	FALSE	NA	NA	FALSE
Small_Intestine_Terminal_Ileum	*SLC5A2*	ENSG00000140675.12	Small_Intestine_Terminal_Ileum_SLC5A2_rs117391625	rs117391625	T	C	0.078	-0.372	0.128	4.22E-03	174	0.005	0.011	6.22E-01	FALSE	NA	NA	FALSE
Brain_Anterior_cingulate_cortex_BA24	*SLC5A2*	ENSG00000140675.12	Brain_Anterior_cingulate_cortex_BA24_SLC5A2_rs4613077	rs4613077	A	C	0.017	-1.200	0.430	6.13E-03	147	NA	NA	NA	FALSE	NA	NA	FALSE
Cells_Cultured_fibroblasts	*SLC5A2*	ENSG00000140675.12	Cells_Cultured_fibroblasts_SLC5A2_rs76378339	rs76378339	T	C	0.009	0.630	0.232	6.94E-03	483	-0.034	0.014	1.35E-02	TRUE	rs11644104_C_T	7%	FALSE
Testis	*SLC5A2*	ENSG00000140675.12	Testis_SLC5A2_rs35530358	rs35530358	C	CGT	0.717	0.086	0.032	7.83E-03	322	NA	NA	NA	FALSE	NA	NA	FALSE

Notes 1: After instrument selection, eight variants for nine gene-tissue combination passed the selection process. After LD clumping of these eight variants with r2<0.1, two variants were kept as primary instruments (listed in eTable S2)

Notes 2: Tissus, Gene.name, ENSG.ID and Phenotype are the expression levels of a gene in a specific tissue that had been proxied by the genetic instruments. Variant ID, CHR and Position are the ID of the genetic variant. Effect allele, other allele, effect allele freq, beta, SE, N and P are the genetic association information of the genetic variant on the exposure. Beta.mr, se.mr, pval.mr are the effect, standard error and p value of the MR analysis of expression of SCL5A2 on HbA1c. HbA1c-signal-in-region, LD-r2 and pass.LD.check are the approximate colocalization that been conducted between expression of SLC5A2 gene and HbA1c.

Supplementary Table 4. Genetic instruments been selected to proxy SGLT2 inhibition and HbA1c levels (data from MAGIC consortium). Related to STAR Methods

Phenotype	SNP	Effect_allele	Other_allele	Effect_allele_fre	Beta	Se	P	N	maf	r2 - variance e	Sum_r2	N_SNPs	F-statistics
SGLT2_MAGIC_new	rs8050500	C	T	0.467	-0.043	0.009	1.56E-06	128609	0.467	9.06E-04	9.06E-04	2	58.295

Notes: Phenotype is the exposure that been proxied by the genetic instruments. id.phenotype is the IEU OpenGWAS database ID of the outcome. Variant ID, CHR and Position are the ID, chromosome and position of the genetic variant. Effect allele, other allele, effect allele freq, beta, SE, N and P are the genetic association information of the genetic variant on the exposure.

Supplementary Table 5. Characteristics of outcome data been used in this study. Related to STAR Methods

Outcome data information								
**MR-phenotype name**	**Purpose**	**IEU-OpenGWASdb-ID**	**Case group**	**Control group**	**GWAS Model**	**n_cases**	**n_controls**	**n_total**
Total prostate cancer	PrCa risk	ieu-b-85	All PrCa cases	Non-PrCa controls	Logistic	79148	61106	140254
Advanced prostate cancer	PrCa risk	ieu-a-1238	Advanced PrCa	Non-PrCa controls	Logistic	15167	58308	73475
Early-onset prostate cancer	PrCa risk	ieu-a-1240	PrCa Age at Dx<=55	Non-PrCa controls	Logistic	6988	44256	51244
High vs low aggressive prostate cancer	PrCa risk	ieu-a-1243	High aggressive	Low aggressive	Logistic	15561	9739	25300
High vs low and intermediate aggressive prostate cancer	PrCa risk	ieu-a-1244	High aggressive	Low/intermediate aggressive	Logistic	20658	38093	58751
Advaced vs non-advanced prostate cancer	PrCa risk	ieu-a-1241	Advanced PrCa	Non Advanced PrCa	Logistic	14160	62421	76581
Gleason score	PrCa risk	ieu-a-1242	Continuous score	/	Linear	/	/	61978
PSA levels	PrCa diagnosis	/	Continuous level	/	Linear	/	/	95768
Type 2 diabetes	Validation	Mahajan 2018	Type 2 diabetes	/	Logistic	14160	62421	76581

* low aggressive:	T stage from the TNM staging<=T1, and Gleason score (GS)<=6, and PSA<10;
** intermediate aggressive	T stage: T2, and GS=7, and PSA 10~20;
*** high aggressive	T stage: T3/T4 or N1 or M1 or GS>=8 or PSA>20;
# advanced	Metastatic disease or GS>=8 or PSA>100 or PrCa Death.

Supplementary Table 6. Mendelian randomization estimates of SGLT2 on prostate cancer using seven instruments selected from UK Biobank. Related to STAR Methods

exposure	outcome	method	nsnp	b	se	lci	uci	pval	Q	Q_df	Q_pval	egger_interc	se	pval	OR	LCI	UCI
SGLT2 conventional male	Advanced prostate cancer	MR Egger	7	1.866	0.939	0.025	3.707	0.104	4.029	5	0.545	-0.028	0.020	0.226	0.155	0.025	0.975
SGLT2 conventional male	Advanced prostate cancer	Weighted median	7	0.660	0.420	-0.163	1.483	0.116	NA	NA	NA	NA	NA	NA	0.517	0.227	1.177
SGLT2 conventional male	Advanced prostate cancer	Inverse variance weighted	7	0.655	0.332	0.003	1.306	0.049	5.931	6	0.431	NA	NA	NA	0.519	0.271	0.997
SGLT2 conventional male	Advanced prostate cancer	Simple mode	7	0.533	0.664	-0.769	1.834	0.453	NA	NA	NA	NA	NA	NA	0.587	0.160	2.158
SGLT2 conventional male	Advanced prostate cancer	Weighted mode	7	0.625	0.465	-0.287	1.537	0.228	NA	NA	NA	NA	NA	NA	0.535	0.215	1.332
SGLT2 conventional male	Advanced prostate cancer	gIVW	7	0.739	0.384	-0.014	1.492	0.054	4.974	6	0.547	NA	NA	NA	0.477	0.225	1.014
SGLT2 conventional male	Early-onset prostate cancer	MR Egger	7	0.652	1.386	-2.065	3.369	0.658	4.197	5	0.521	0.015	0.030	0.641	0.521	0.034	7.883
SGLT2 conventional male	Early-onset prostate cancer	Weighted median	7	0.792	0.645	-0.473	2.057	0.220	NA	NA	NA	NA	NA	NA	0.453	0.128	1.604
SGLT2 conventional male	Early-onset prostate cancer	Inverse variance weighted	7	1.296	0.488	0.339	2.252	0.008	4.443	6	0.617	NA	NA	NA	0.274	0.105	0.712
SGLT2 conventional male	Early-onset prostate cancer	Simple mode	7	0.869	0.943	-0.978	2.717	0.392	NA	NA	NA	NA	NA	NA	0.419	0.066	2.660
SGLT2 conventional male	Early-onset prostate cancer	Weighted mode	7	0.799	0.676	-0.526	2.123	0.282	NA	NA	NA	NA	NA	NA	0.450	0.120	1.692
SGLT2 conventional male	Early-onset prostate cancer	gIVW	7	1.090	0.563	-0.013	2.193	0.053	5.271	6	0.509	NA	NA	NA	0.336	0.112	1.014
SGLT2 conventional male	Advaced vs non-advanced prostate cancer	MR Egger	7	2.502	0.947	0.645	4.359	0.046	4.280	5	0.510	-0.054	0.020	0.045	0.082	0.013	0.524
SGLT2 conventional male	Advaced vs non-advanced prostate cancer	Weighted median	7	0.703	0.445	-0.169	1.576	0.114	NA	NA	NA	NA	NA	NA	0.495	0.207	1.185
SGLT2 conventional male	Advaced vs non-advanced prostate cancer	Inverse variance weighted	7	0.148	0.461	-0.756	1.052	0.749	11.343	6	0.078	NA	NA	NA	0.863	0.349	2.130
SGLT2 conventional male	Advaced vs non-advanced prostate cancer	Simple mode	7	-0.588	0.732	-2.023	0.847	0.453	NA	NA	NA	NA	NA	NA	1.800	0.429	7.563
SGLT2 conventional male	Advaced vs non-advanced prostate cancer	Weighted mode	7	0.755	0.477	-0.179	1.690	0.164	NA	NA	NA	NA	NA	NA	0.470	0.185	1.196
SGLT2 conventional male	Advaced vs non-advanced prostate cancer	gIVW	7	0.296	0.474	-0.634	1.226	0.533	8.987	6	0.174	NA	NA	NA	0.744	0.294	1.885
SGLT2 conventional male	Gleason score	MR Egger	7	0.602	0.408	-0.198	1.402	0.200	4.984	5	0.418	-0.016	0.009	0.137	NA	NA	NA
SGLT2 conventional male	Gleason score	Weighted median	7	0.090	0.192	-0.286	0.465	0.640	NA	NA	NA	NA	NA	NA	NA	NA	NA
SGLT2 conventional male	Gleason score	Inverse variance weighted	7	-0.072	0.169	-0.404	0.260	0.671	8.106	6	0.230	NA	NA	NA	NA	NA	NA
SGLT2 conventional male	Gleason score	Simple mode	7	-0.038	0.286	-0.598	0.522	0.898	NA	NA	NA	NA	NA	NA	NA	NA	NA
SGLT2 conventional male	Gleason score	Weighted mode	7	0.124	0.188	-0.245	0.493	0.535	NA	NA	NA	NA	NA	NA	NA	NA	NA
SGLT2 conventional male	Gleason score	gIVW	7	-0.052	0.171	-0.387	0.283	0.763	6.146	6	0.407	NA	NA	NA	NA	NA	NA
SGLT2 conventional male	High vs low aggressive prostate cancer	MR Egger	7	0.755	1.660	-2.498	4.008	0.668	7.465	5	0.188	-0.021	0.036	0.592	0.470	0.018	12.158
SGLT2 conventional male	High vs low aggressive prostate cancer	Weighted median	7	0.140	0.640	-1.113	1.393	0.827	NA	NA	NA	NA	NA	NA	0.869	0.248	3.045
SGLT2 conventional male	High vs low aggressive prostate cancer	Inverse variance weighted	7	-0.132	0.556	-1.221	0.957	0.812	7.953	6	0.242	NA	NA	NA	1.141	0.384	3.392
SGLT2 conventional male	High vs low aggressive prostate cancer	Simple mode	7	0.170	1.075	-1.937	2.277	0.880	NA	NA	NA	NA	NA	NA	0.844	0.103	6.936
SGLT2 conventional male	High vs low aggressive prostate cancer	Weighted mode	7	0.339	0.721	-1.075	1.753	0.655	NA	NA	NA	NA	NA	NA	0.713	0.173	2.931
SGLT2 conventional male	High vs low aggressive prostate cancer	gIVW	7	-0.364	0.648	-1.634	0.906	0.574	8.093	6	0.231	NA	NA	NA	1.439	0.404	5.125
SGLT2 conventional male	High vs low and intermediate aggressive prostate cancer	MR Egger	7	1.644	0.883	-0.088	3.375	0.122	3.432	5	0.634	-0.029	0.019	0.183	0.193	0.034	1.092
SGLT2 conventional male	High vs low and intermediate aggressive prostate cancer	Weighted median	7	0.627	0.411	-0.178	1.432	0.127	NA	NA	NA	NA	NA	NA	0.534	0.239	1.195
SGLT2 conventional male	High vs low and intermediate aggressive prostate cancer	Inverse variance weighted	7	0.368	0.314	-0.247	0.983	0.240	5.818	6	0.444	NA	NA	NA	0.692	0.374	1.280
SGLT2 conventional male	High vs low and intermediate aggressive prostate cancer	Simple mode	7	0.490	0.705	-0.891	1.871	0.513	NA	NA	NA	NA	NA	NA	0.613	0.154	2.439
SGLT2 conventional male	High vs low and intermediate aggressive prostate cancer	Weighted mode	7	0.647	0.475	-0.283	1.577	0.222	NA	NA	NA	NA	NA	NA	0.524	0.207	1.328
SGLT2 conventional male	High vs low and intermediate aggressive prostate cancer	gIVW	7	0.303	0.363	-0.409	1.015	0.404	5.333	6	0.502	NA	NA	NA	0.739	0.362	1.505
SGLT2 conventional male	Total prostate cancer	MR Egger	7	0.532	0.545	-0.537	1.601	0.374	3.069	5	0.689	0.001	0.012	0.923	0.587	0.202	1.710
SGLT2 conventional male	Total prostate cancer	Weighted median	7	0.592	0.247	0.109	1.076	0.016	NA	NA	NA	NA	NA	NA	0.553	0.341	0.897
SGLT2 conventional male	Total prostate cancer	Inverse variance weighted	7	0.584	0.194	0.204	0.964	0.003	3.079	6	0.799	NA	NA	NA	0.557	0.381	0.815
SGLT2 conventional male	Total prostate cancer	Simple mode	7	0.435	0.324	-0.199	1.070	0.227	NA	NA	NA	NA	NA	NA	0.647	0.343	1.221
SGLT2 conventional male	Total prostate cancer	Weighted mode	7	0.542	0.256	0.040	1.044	0.079	NA	NA	NA	NA	NA	NA	0.581	0.352	0.961
SGLT2 conventional male	Total prostate cancer	gIVW	7	0.571	0.225	0.130	1.011	0.011	2.618	6	0.855	NA	NA	NA	0.565	0.364	0.878
SGLT2 conventional male	PSA levels	MR Egger	7	-0.540	0.235	-1.000	-0.080	0.070	1.912	5	0.861	0.010	0.005	0.124	NA	NA	NA
SGLT2 conventional male	PSA levels	Weighted median	7	-0.125	0.111	-0.342	0.093	0.263	NA	NA	NA	NA	NA	NA	NA	NA	NA
SGLT2 conventional male	PSA levels	Inverse variance weighted	7	-0.137	0.085	-0.304	0.030	0.107	5.313	6	0.504	NA	NA	NA	NA	NA	NA
SGLT2 conventional male	PSA levels	Simple mode	7	-0.009	0.167	-0.336	0.318	0.959	NA	NA	NA	NA	NA	NA	NA	NA	NA
SGLT2 conventional male	PSA levels	Weighted mode	7	-0.126	0.111	-0.343	0.092	0.300	NA	NA	NA	NA	NA	NA	NA	NA	NA
SGLT2 conventional male	PSA levels	gIVW	7	-0.132	0.102	-0.331	0.067	0.195	6.387	6	0.381	NA	NA	NA	NA	NA	NA
SGLT2 conventional male	Type 2 diabetes	Inverse variance weighted	6	0.422	0.150	0.128	0.715	0.005	3.087	5	0.687	NA	NA	NA	0.656	0.489	0.880
SGLT2 conventional male	Type 2 diabetes	MR Egger	6	-0.150	0.420	-0.974	0.674	0.739	0.967	4	0.915	0.013	0.009	0.219	1.162	0.510	2.649
SGLT2 conventional male	Type 2 diabetes	Weighted median	6	0.244	0.195	-0.138	0.627	0.211	NA	NA	NA	NA	NA	NA	0.783	0.534	1.148
SGLT2 conventional male	Type 2 diabetes	Simple mode	6	0.263	0.306	-0.336	0.862	0.429	NA	NA	NA	NA	NA	NA	0.769	0.422	1.399
SGLT2 conventional male	Type 2 diabetes	Weighted mode	6	0.256	0.202	-0.141	0.652	0.262	NA	NA	NA	NA	NA	NA	0.774	0.521	1.151
SGLT2 conventional male	Type 2 diabetes	gIVW	6	0.271	0.245	-0.208	0.751	0.268	7.673	4	0.175	NA	NA	NA	0.762	0.472	1.232

Notes: the conventional instrument selection process refers to seven genetic variants robustly associated with HbA1c (P<5e-8) in the *SLC5A2* region. nsnp means the number of instruments been used in the MR analysis. Method is the MR method been used in the analysis. Beta, se, pval, ci.lb

Supplementary Table 7. Mendelian randomization estimates of SGLT2 on prostate cancer based on the instruments selected either using a stringent selection process or from MAGIC data. Related to STAR Methods

exposure	outcome	method	nsnp	b	se	lci	uci	pval	Q	Q_df	Q_pval	egger_interc	se	pval	OR	LCI	UCI
SGLT2 ukbb male	Advanced prostate cancer	IVW	2	1.375	0.702	0.000	2.750	0.050	0.047	1	0.829	NA	NA	NA	0.253	0.064	1.000
SGLT2 ukbb male	Advanced prostate cancer	gIVW	2	1.401	0.785	-0.138	2.940	0.074	0.064	1	0.800	NA	NA	NA	0.246	0.053	1.147
SGLT2 ukbb male	Early-onset prostate cancer	IVW	2	0.591	1.757	-2.853	4.034	0.737	2.947	1	0.086	NA	NA	NA	0.554	0.018	17.334
SGLT2 ukbb male	Early-onset prostate cancer	gIVW	2	0.287	1.146	-1.958	2.532	0.802	4.063	1	0.044	NA	NA	NA	0.750	0.079	7.087
SGLT2 ukbb male	Advaced vs non-advanced prostate cancer	IVW	2	-0.085	0.709	-1.475	1.306	0.905	0.182	1	0.669	NA	NA	NA	1.088	0.271	4.371
SGLT2 ukbb male	Advaced vs non-advanced prostate cancer	gIVW	2	-0.033	0.794	-1.590	1.524	0.967	0.252	1	0.616	NA	NA	NA	1.033	0.218	4.902
SGLT2 ukbb male	Gleason score	IVW	2	0.132	0.310	-0.476	0.740	0.670	0.853	1	0.356	NA	NA	NA	0.876	0.477	1.609
SGLT2 ukbb male	Gleason score	gIVW	2	0.179	0.348	-0.503	0.861	0.607	1.182	1	0.277	NA	NA	NA	0.836	0.423	1.654
SGLT2 ukbb male	High vs low aggressive prostate cancer	IVW	2	0.830	1.061	-1.250	2.911	0.434	1.070	1	0.301	NA	NA	NA	0.436	0.054	3.489
SGLT2 ukbb male	High vs low aggressive prostate cancer	gIVW	2	0.654	1.151	-1.601	2.910	0.570	1.480	1	0.224	NA	NA	NA	0.520	0.055	4.958
SGLT2 ukbb male	High vs low and intermediate aggressive prostate cancer	IVW	2	-0.037	0.665	-1.341	1.266	0.955	0.031	1	0.861	NA	NA	NA	1.038	0.282	3.821
SGLT2 ukbb male	High vs low and intermediate aggressive prostate cancer	gIVW	2	-0.018	0.745	-1.479	1.443	0.981	0.042	1	0.837	NA	NA	NA	1.018	0.236	4.387
SGLT2 ukbb male	Total prostate cancer	IVW	2	1.240	0.410	0.436	2.044	0.002	0.077	1	0.782	NA	NA	NA	0.289	0.129	0.646
SGLT2 ukbb male	Total prostate cancer	gIVW	2	1.221	0.459	0.320	2.122	0.008	0.106	1	0.745	NA	NA	NA	0.295	0.120	0.726
SGLT2 ukbb male	PSA levels	IVW	2	-0.150	0.195	-0.532	0.232	0.442	0.411	1	0.521	NA	NA	NA	1.162	0.793	1.702
SGLT2 ukbb male	PSA levels	gIVW	2	-0.164	0.221	-0.597	0.268	0.456	0.580	1	0.446	NA	NA	NA	1.179	0.765	1.816
SGLT2_MAGIC	Advanced prostate cancer	Wald ratio	1	0.898	0.351	0.209	1.586	0.011	NA	NA	NA	NA	NA	NA	0.408	0.205	0.811
SGLT2_MAGIC	Early-onset prostate cancer	Wald ratio	1	0.372	0.516	-0.640	1.384	0.471	NA	NA	NA	NA	NA	NA	0.689	0.251	1.896
SGLT2_MAGIC	Advaced vs non-advanced prostate cancer	Wald ratio	1	0.712	0.356	0.014	1.409	0.046	NA	NA	NA	NA	NA	NA	0.491	0.244	0.986
SGLT2_MAGIC	Gleason score	Wald ratio	1	0.130	0.156	-0.175	0.436	0.403	NA	NA	NA	NA	NA	NA	0.878	0.647	1.191
SGLT2_MAGIC	High vs low aggressive prostate cancer	Wald ratio	1	0.065	0.512	-0.938	1.068	0.899	NA	NA	NA	NA	NA	NA	0.937	0.344	2.554
SGLT2_MAGIC	High vs low and intermediate aggressive prostate cancer	Wald ratio	1	0.433	0.335	-0.224	1.089	0.196	NA	NA	NA	NA	NA	NA	0.649	0.337	1.251
SGLT2_MAGIC	Total prostate cancer	Wald ratio	1	0.416	0.207	0.011	0.822	0.044	NA	NA	NA	NA	NA	NA	0.659	0.440	0.989
SGLT2_MAGIC	PSA levels	Wald ratio	1	-0.114	0.086	-0.283	0.055	0.185	NA	NA	NA	NA	NA	NA	1.121	0.947	1.327

Notes: the conventional instrument selection process refers to seven genetic variants robustly associated with HbA1c (P<5e-8) in the *SLC5A2* region. nsnp means the number of instruments been used in the MR analysis. Method is the MR method been used in the analysis. Beta, se, pval, ci.lb and ci.ub are the MR effect estimate, standard error, P value, lower and upper confidence intervals of the exposure on the outcome. Q, Q_df and Q_pval are the statistics that measuring heterogeneity across studies. Egger_intercept, egger_se and egger_pval are statistics to estimating levels of pleiotropy.OR, LCI and UCI are the odds ratio, lower and uppper confidence intervals of MR effect scaled from log odds ratio to odds ratio.

Supplementary Table 8. Genetic colocalization estimates of SGLT2 (proxied by HbA1c) on prostate cancer in the SCL5A2 region. Related to STAR Methods

Trait1	Trait2	nsnp	PP.H4/(PP.H3+PP.H4)
SGLT2 (proxied by its HbA1c lowering effect)	Total prostate cancer	2291	71.91%
SGLT2 (proxied by expression levels of *SLC5A2*)	Total prostate cancer	256	90.75%

Notes: we are under the assumption that PP.H0, PP.H1 and PP.H2 were unlikely to be true given strong MR evidence to support a genetic signal in both exposure (trait1) and outcome (trait2). We therefore estimated the probability of PP.H4/(PP.H3+PP.H4) as the evidence source for colocalization. nsnp means the number of genetic variants been used in the colocalization analysis.

Supplementary Table 9. Phenome-wide association (PheWAS) results of SLGT2 instruments. The PheWAS association with P value < 1e-5 was listed in this table. Related to STAR Methods

Phenotype	id.phenotype	Variant ID	chr	position	Effect_allele	Other_allele	Effect_allele_	beta	se	p	n
Red cell distribution width	ebi-a-GCST90002404	rs8050500	16	31404571	C	T	0.446	-0.026	0.002	4.00E-35	408112
High light scatter reticulocyte percentage of red cells	ebi-a-GCST90002386	rs8050500	16	31404571	C	T	0.446	0.019	0.002	1.80E-19	408112
Sum basophil neutrophil counts	ebi-a-GCST004621	rs8050500	16	31404571	C	T	0.446	-0.032	0.004	5.22E-19	171529
Hip circumference	ukb-b-15590	rs55766044	16	31117698	T	C	0.280	0.018	0.002	1.70E-15	462117
Weight	ukb-b-11842	rs55766044	16	31117698	T	C	0.280	0.015	0.002	1.30E-14	461632
Red cell distribution width	ebi-a-GCST006804	rs8050500	16	31404571	C	T	0.446	-0.030	0.004	1.40E-14	116666
Waist circumference	ukb-b-9405	rs55766044	16	31117698	T	C	0.280	0.015	0.002	5.70E-14	462166
Trunk fat mass	ukb-b-20044	rs55766044	16	31117698	T	C	0.280	0.017	0.002	7.00E-14	454588
Weight	ukb-b-12039	rs55766044	16	31117698	T	C	0.280	0.014	0.002	1.00E-13	454893
Arm fat mass (right)	ukb-b-6704	rs55766044	16	31117698	T	C	0.280	0.016	0.002	1.30E-13	454757
Arm fat mass (left)	ukb-b-8338	rs55766044	16	31117698	T	C	0.280	0.016	0.002	2.20E-13	454684
Whole body fat mass	ukb-b-19393	rs55766044	16	31117698	T	C	0.280	0.016	0.002	6.50E-13	454137
Trunk fat percentage	ukb-b-16407	rs55766044	16	31117698	T	C	0.280	0.014	0.002	3.30E-12	454613
Leg fat mass (right)	ukb-b-18096	rs55766044	16	31117698	T	C	0.280	0.012	0.002	6.50E-12	454846
Arm fat percentage (right)	ukb-b-12854	rs55766044	16	31117698	T	C	0.280	0.012	0.002	7.80E-12	454789
Body fat percentage	ukb-b-8909	rs55766044	16	31117698	T	C	0.280	0.012	0.002	7.80E-12	454633
Low density lipoprotein cholesterol levels	ebi-a-GCST90002412	rs55766044	16	31117698	T	C	0.280	0.015	0.002	3.90E-13	431167
Leg fat mass (left)	ukb-b-7212	rs55766044	16	31117698	T	C	0.280	0.012	0.002	8.80E-12	454823
Arm fat percentage (left)	ukb-b-20188	rs55766044	16	31117698	T	C	0.280	0.012	0.002	9.10E-12	454724
diastolic blood pressure	ieu-b-39	rs55766044	16	31117698	T	C	0.279	0.130	0.020	5.04E-11	721678
Red cell distribution width	ebi-a-GCST90002404	rs28692853	16	31573030	A	C	0.506	-0.013	0.002	4.60E-10	408112
Basal metabolic rate	ukb-b-16446	rs55766044	16	31117698	T	C	0.280	0.009	0.001	2.60E-09	454874
Leg fat percentage (left)	ukb-b-18377	rs55766044	16	31117698	T	C	0.280	0.008	0.001	3.30E-09	454826
Leg fat percentage (right)	ukb-b-20531	rs55766044	16	31117698	T	C	0.280	0.008	0.001	4.20E-09	454854
Body mass index (BMI)	ukb-b-19953	rs55766044	16	31117698	T	C	0.280	0.013	0.002	4.30E-09	461460
Alzheimer's disease or family history of Alzheimer's disease	ebi-a-GCST90012877	rs55766044	16	31117698	T	C	0.280	-0.062	0.011	7.32E-09	472868
Body mass index (BMI)	ukb-b-2303	rs55766044	16	31117698	T	C	0.280	0.013	0.002	7.40E-09	454884
Arm predicted mass (left)	ukb-b-9093	rs55766044	16	31117698	T	C	0.280	0.008	0.001	1.30E-08	454655
Snoring	ukb-b-17400	rs55766044	16	31117698	T	C	0.280	-0.006	0.001	1.60E-08	430438
Diastolic blood pressure, automated reading	ukb-b-7992	rs55766044	16	31117698	T	C	0.280	0.013	0.002	1.80E-08	436424
Red cell distribution width	ebi-a-GCST90002404	rs28675289	16	31463252	T	C	0.045	-0.029	0.005	2.10E-08	408112
Worry	ebi-a-GCST006478	rs55766044	16	31117698	T	C	NA	-0.015	0.003	3.30E-08	348219
Snoring	ebi-a-GCST009760	rs55766044	16	31117698	T	C	NA	0.006	0.001	4.30E-08	408317
Comparative body size at age 10	ukb-b-4650	rs55766044	16	31117698	T	C	0.280	0.009	0.002	4.40E-08	454718

Notes: Phenotype is the exposure that been proxied by the genetic instruments. id.phenotype is the IEU OpenGWAS database ID of the outcome. Variant ID, CHR and Position are the ID, chromosome and position of the genetic variant. Effect allele, other allele, effect allele freq, beta, SE, N and P are the genetic association information of the genetic variant on the exposure.

Supplementary Table 10. Genetic instruments used for the multivariable Mendelian randomization model and the multivariable Mendelian randomization results. Related to STAR Methods

Supplementary Table 10A. Genetic instruments for SGLT2 and red blood cell distribution been used in the multivariable Mendelian randomization model. Related to STAR Methods

Phenotype	SNP	Effect_allele	Other_allele	Effect_allele_freq	Beta	Se	P
SGLT2 primary	rs1232538	T	G	0.273	0.011	0.004	4.20E-03
SGLT2 primary	rs28675289	T	C	0.044	-0.040	0.008	2.66E-06
SGLT2 primary	rs28692853	A	C	0.507	-0.018	0.003	3.16E-07
SGLT2 primary	rs45625038	T	C	0.030	0.031	0.010	2.45E-03
SGLT2 primary	rs55766044	T	C	0.280	0.017	0.004	1.54E-05
SGLT2 primary	rs557720784	T	C	0.054	0.029	0.008	2.16E-04
SGLT2 primary	rs8050500	C	T	0.446	-0.029	0.003	2.03E-17
Red blood cell (erythrocyte) count	rs1232538	T	G	0.273	-0.003	0.002	2.03E-01
Red blood cell (erythrocyte) count	rs28675289	T	C	0.044	-0.003	0.005	5.97E-01
Red blood cell (erythrocyte) count	rs28692853	A	C	0.508	-0.002	0.002	2.68E-01
Red blood cell (erythrocyte) count	rs45625038	T	C	0.030	-0.009	0.006	1.29E-01
Red blood cell (erythrocyte) count	rs55766044	T	C	0.280	-0.015	0.002	1.11E-10
Red blood cell (erythrocyte) count	rs557720784	T	C	0.053	0.000	0.005	9.24E-01
Red blood cell (erythrocyte) count	rs8050500	C	T	0.446	-0.009	0.002	8.67E-06

Phenotype	SNP	Effect_allele	Other_allele	Effect_allele_freq	Beta	Se	P
SGLT2 primary	rs1232538	T	G	0.273	0.011	0.004	4.20E-03
SGLT2 primary	rs28675289	T	C	0.044	-0.040	0.008	2.66E-06
SGLT2 primary	rs28692853	A	C	0.507	-0.018	0.003	3.16E-07
SGLT2 primary	rs45625038	T	C	0.030	0.031	0.010	2.45E-03
SGLT2 primary	rs55766044	T	C	0.280	0.017	0.004	1.54E-05
SGLT2 primary	rs8050500	C	T	0.446	-0.029	0.003	2.03E-17
Body mass index	rs1232538	T	G	0.602	0.003	0.003	2.70E-01
Body mass index	rs28675289	T	C	0.678	-0.008	0.006	2.30E-01
Body mass index	rs28692853	A	C	0.491	0.002	0.003	4.40E-01
Body mass index	rs45625038	T	C	0.693	0.002	0.011	8.80E-01
Body mass index	rs55766044	T	C	0.588	0.014	0.003	1.20E-05
Body mass index	rs8050500	C	T	0.523	0.005	0.003	5.90E-02

Phenotype	SNP	Effect_allele	Other_allele	Effect_allele_freq	Beta	Se	P
SGLT2 primary	rs1232538	T	G	0.273	0.011	0.004	4.20E-03
SGLT2 primary	rs28675289	T	C	0.044	-0.040	0.008	2.66E-06
SGLT2 primary	rs28692853	A	C	0.507	-0.018	0.003	3.16E-07
SGLT2 primary	rs45625038	T	C	0.030	0.031	0.010	2.45E-03
SGLT2 primary	rs55766044	T	C	0.280	0.017	0.004	1.54E-05
SGLT2 primary	rs557720784	T	C	0.054	0.029	0.008	2.16E-04
SGLT2 primary	rs8050500	C	T	0.446	-0.029	0.003	2.03E-17
Low density lipoprotein cholesterol levels	rs1232538	T	G	0.274	0.004	0.002	9.70E-02
Low density lipoprotein cholesterol levels	rs28675289	T	C	0.045	-0.010	0.005	4.00E-02
Low density lipoprotein cholesterol levels	rs28692853	A	C	0.506	0.000	0.002	9.00E-01
Low density lipoprotein cholesterol levels	rs45625038	T	C	0.029	0.008	0.006	1.50E-01
Low density lipoprotein cholesterol levels	rs55766044	T	C	0.280	0.015	0.002	3.90E-13
Low density lipoprotein cholesterol levels	rs557720784	T	C	0.052	-0.008	0.004	8.40E-02
Low density lipoprotein cholesterol levels	rs8050500	C	T	0.445	0.001	0.002	6.80E-01

Phenotype	SNP	Effect_allele	Other_allele	Effect_allele_freq	Beta	Se	P
SGLT2 primary	rs1232538	T	G	0.273	0.011	0.004	4.20E-03
SGLT2 primary	rs28675289	T	C	0.044	-0.040	0.008	2.66E-06
SGLT2 primary	rs28692853	A	C	0.507	-0.018	0.003	3.16E-07
SGLT2 primary	rs45625038	T	C	0.030	0.031	0.010	2.45E-03
SGLT2 primary	rs55766044	T	C	0.280	0.017	0.004	1.54E-05
SGLT2 primary	rs8050500	C	T	0.446	-0.029	0.003	2.03E-17
diastolic blood pressure	rs1232538	T	G	0.280	-0.003	0.019	8.91E-01
diastolic blood pressure	rs28675289	T	C	0.047	-0.006	0.044	8.86E-01
diastolic blood pressure	rs28692853	A	C	0.499	0.012	0.017	4.86E-01
diastolic blood pressure	rs45625038	T	C	0.028	-0.018	0.058	7.64E-01
diastolic blood pressure	rs55766044	T	C	0.279	0.130	0.020	5.04E-11
diastolic blood pressure	rs8050500	C	T	0.445	-0.009	0.018	6.11E-01

Phenotype	SNP	Effect_allele	Other_allele	Effect_allele_freq	Beta	Se	P
SGLT2 primary	rs1232538	T	G	0.273	0.011	0.004	4.20E-03
SGLT2 primary	rs28675289	T	C	0.044	-0.040	0.008	2.66E-06
SGLT2 primary	rs28692853	A	C	0.507	-0.018	0.003	3.16E-07
SGLT2 primary	rs45625038	T	C	0.030	0.031	0.010	2.45E-03
SGLT2 primary	rs55766044	T	C	0.280	0.017	0.004	1.54E-05
SGLT2 primary	rs8050500	C	T	0.446	-0.029	0.003	2.03E-17
Type 2 diabetes	rs1232538	T	G	0.290	0.0051	0.0042	0.227
Type 2 diabetes	rs28675289	T	C	0.055	-0.0059	0.0084	0.482
Type 2 diabetes	rs28692853	A	C	0.489	-0.0089	0.0037	0.016
Type 2 diabetes	rs45625038	T	C	0.023	-0.0032	0.0131	0.807
Type 2 diabetes	rs55766044	T	C	0.291	0.0114	0.0041	0.005
Type 2 diabetes	rs8050500	C	T	0.442	-0.0074	0.0038	0.048

Notes: Phenotype is the exposure that been proxied by the genetic instruments. Variant ID, CHR and Position are the ID, chromosome and position of the genetic variant. Effect allele, other allele, effect allele freq, beta, SE, N and P are the genetic association information of the genetic variant on the exposure.

Supplementary Table 10B. Multivariable Mendelian randomization estimate of SGLT2 on prostate cancer adjusted for red blood cell distribution. Related to STAR Methods

exposure	outcome	nsnp	beta	se	pval	ci.lb	ci.ub	OR	LCI	UCI
SGLT2 primary	Prostate cancer	7	0.580	0.143	4.96E-05	0.300	0.861	0.560	0.423	0.741
Red blood cell (erythrocyte) count	Prostate cancer	7	0.299	0.367	0.416	-0.421	1.019	0.741	0.361	1.524
**exposure**	**outcome**	**nsnp**	**beta**	**se**	**pval**	**ci.lb**	**ci.ub**	**OR**	**LCI**	**UCI**
SGLT2 primary	Prostate cancer	6	0.538	0.079	8.64E-12	0.383	0.692	0.584	0.500	0.681
Body mass index	Prostate cancer	6	-0.387	0.223	0.082	-0.824	0.049	1.473	0.952	2.280
**exposure**	**outcome**	**nsnp**	**beta**	**se**	**pval**	**ci.lb**	**ci.ub**	**OR**	**LCI**	**UCI**
SGLT2 primary	Prostate cancer	7	0.690	0.129	9.32E-08	0.436	0.943	0.502	0.390	0.646
Low density lipoprotein cholesterol levels	Prostate cancer	7	-0.663	0.352	0.060	-1.354	0.028	1.941	0.973	3.872
**exposure**	**outcome**	**nsnp**	**beta**	**se**	**pval**	**ci.lb**	**ci.ub**	**OR**	**LCI**	**UCI**
SGLT2 primary	Prostate cancer	6	0.561	0.090	3.91E-10	0.386	0.737	0.570	0.478	0.680
diastolic blood pressure	Prostate cancer	6	-0.044	0.030	0.148	-0.104	0.016	1.045	0.985	1.110
**exposure**	**outcome**	**nsnp**	**beta**	**se**	**pval**	**ci.lb**	**ci.ub**	**OR**	**LCI**	**UCI**
SGLT2 primary	Prostate cancer	6	0.494	0.212	1.97E-02	0.079	0.909	0.610	0.403	0.924
Type 2 diabetes	Prostate cancer	6	0.054	0.536	0.920	-0.996	1.104	0.947	0.332	2.707

Notes: nsnp means the number of instruments been used in the MR analysis. Beta, se, pval, ci.lb and ci.ub are the MR effect estimate, standard error, P value, lower and upper confidence intervals of the exposure on the outcome. OR, LCI and UCI are the odds ratio, lower and uppper confidence intervals of MR effect scaled from log odds ratio to odds ratio.

Supplementary Table 11. Genetic associations of the two SGLT2 instruments on expression level of other genes in nearby gene regions. Genetic variants with genetic association P<1e-4 was listed in this table. Related to STAR Methods

Gene ID	Gene	Variant ID	CHR	Position	Effect_allele	Other_allele	Effect_allele_freq	Beta	SE	N	P	Known glycemic gene?	Drug	Drug function
ENSG00000131797	CLUHP3	rs35445454	16	31699326	T	C	0.336	-0.549	0.012	17997	3.27E-300	NO	NA	NA
ENSG00000156886	ITGAD	rs9930811	16	31400360	G	A	0.351	-0.290	0.012	31346	2.67E-124	NO	NA	NA
ENSG00000261731	CTD-2358C21.4	rs35445454	16	31699326	T	C	0.336	-0.292	0.012	3831	2.70E-123	NO	NA	NA
ENSG00000140678	ITGAX	rs9930811	16	31400360	G	A	0.351	-0.287	0.012	26057	1.19E-121	NO	NA	NA
ENSG00000131797	CLUHP3	rs9930811	16	31400360	G	A	0.351	-0.180	0.012	22545	3.91E-48	NO	NA	NA
ENSG00000140678	ITGAX	rs35445454	16	31699326	T	C	0.336	-0.181	0.013	21723	3.30E-47	NO	NA	NA
ENSG00000140691	ARMC5	rs9930811	16	31400360	G	A	0.351	0.165	0.012	30597	1.56E-40	NO	NA	NA
ENSG00000156886	ITGAD	rs35445454	16	31699326	T	C	0.336	-0.139	0.013	26798	1.77E-28	NO	NA	NA
ENSG00000103507	BCKDK	rs9930811	16	31400360	G	A	0.351	-0.119	0.012	31346	7.59E-22	NO	NA	NA
ENSG00000140682	TGFB1I1	rs9930811	16	31400360	G	A	0.351	0.119	0.012	31132	8.86E-22	NO	NA	NA
ENSG00000261245	RP11-120K18.3	rs9930811	16	31400360	G	A	0.351	-0.105	0.012	4656	4.16E-17	NO	NA	NA
ENSG00000260911	RP11-196G11.2	rs9930811	16	31400360	G	A	0.351	0.097	0.012	5164	7.38E-15	NO	NA	NA
ENSG00000103496	STX4	rs9930811	16	31400360	G	A	0.351	0.094	0.012	31132	3.41E-14	NO	PHENPROCOUMON	Anticoagulant
ENSG00000197302	ZNF720	rs35445454	16	31699326	T	C	0.336	-0.093	0.013	26758	1.07E-13	NO	NA	NA
ENSG00000167394	ZNF668	rs9930811	16	31400360	G	A	0.351	-0.077	0.012	31346	6.76E-10	NO	NA	NA
ENSG00000103549	RNF40	rs9930811	16	31400360	G	A	0.351	-0.075	0.012	31346	1.45E-09	NO	NA	NA
ENSG00000261731	CTD-2358C21.4	rs9930811	16	31400360	G	A	0.351	-0.070	0.012	4656	1.80E-08	NO	NA	NA
ENSG00000169877	AHSP	rs9930811	16	31400360	G	A	0.351	0.069	0.012	31346	2.67E-08	NO	NA	NA
ENSG00000140688	C16orf58	rs9930811	16	31400360	G	A	0.351	0.064	0.012	31346	2.46E-07	NO	NA	NA
ENSG00000099377	HSD3B7	rs9930811	16	31400360	G	A	0.351	-0.063	0.012	31346	3.74E-07	NO	NA	NA
ENSG00000140691	ARMC5	rs35445454	16	31699326	T	C	0.336	0.064	0.013	26049	3.80E-07	NO	NA	NA
ENSG00000103507	BCKDK	rs35445454	16	31699326	T	C	0.336	-0.061	0.013	26798	1.39E-06	NO	NA	NA
ENSG00000169877	AHSP	rs35445454	16	31699326	T	C	0.336	0.060	0.013	26798	1.59E-06	NO	NA	NA
ENSG00000169896	ITGAM	rs9930811	16	31400360	G	A	0.351	-0.056	0.012	31346	5.98E-06	NO	ROVELIZUMAB	Treat paroxysmal nocturnal hemoglobinuria
ENSG00000140675	SLC5A2	rs9930811	16	31400360	G	A	0.351	0.052	0.012	31306	3.05E-05	NO	SGLT2 inhibitor	Treat diabetes

Notes: The expression data of gene were obtained from whole blood. Gene ID and Gene refers to each gene in the nearby genomic region. Variant ID, CHR and Position are the ID, chromosome and position of the genetic variant. Effect allele, other allele, effect allele freq, beta, SE, N and P are the genetic association information of the genetic variant on the expression of the related gene. Known glycemic gene, drug and function are the annoations of related gene, which refers to whether the gene is a reported gene for any glycemic traits, whether the gene has any interaction with any drug as well as the function of the related drug.

Supplementary Table 12. Association between use of SGLT2i compared with DPP4i and risk of prostate cancer. Related to STAR Methods

	Original cohort							PS-matched cohort						
	SGLT2i			DPP4i			Hazard ratio (95% CI)	SGLT2i			DPP4i			Hazard ratio (95% CI)
	Events	Person years	Incidence rate	Events	Person years	Incidence rate		Events	Person years	Incidence rate	Events	Person years	Incidence rate	
Main analysis	114	25171.09	452.90	574	102208.72	561.60	0.76 (0.61-0.94)	106	22678.6	467.4	224	45458.9	492.75	0.77 (0.61-0.99)
Lag period														
1-month	85	25119.20	338.39	451	102153.20	441.49	0.86 (0.67-1.10)	80	22662.70	353	182	44455.1	409.40	0.81 (0.61-1.07)
2-month	74	24952.08	296.57	423	101978.74	414.79	0.83 (0.64-1.09)	69	22585	305.51	172	43573.6	394.73	0.75 (0.56-1.00)
3-month	67	24733.13	270.89	408	101759.12	400.95	0.81 (0.62-1.07)	63	22425.8	280.93	164	42548.9	385.44	0.78 (0.57-1.06)
6-month	49	23449.53	208.96	339	100444.48	337.50	0.90 (0.66-1.25)	46	21423.9	214.71	143	38982.9	366.83	0.75 (0.53-1.08)

Notes: prostate cancer refers to individuals with incident prostate cancer plus those with total prostate specific antigen [PSA] level>10 ng/mL during the follow-up period SGLT2i. PS-matched cohort refers to 1:1 propensity-score matching cohort of 48,310 patients, sodium-glucose cotransporter-2 inhibitor; CI, confidence interval. The unit of the incidence rate was 100,000 person years.

Supplementary Table 13. Association between HBA1c levels and type 2 diabetes with total prostate cancer. Related to STAR Methods

Supplementary Table 13A. Mendelian randomization estimates of HBA1c levels on total prostate cancer. Related to STAR Methods

exposure	outcome	method	nsnp	b	se	pval	Q	Q_df	Q_pval	lci	uci	OR (inhibitio	OR_UCI	OR_LCL
Glycated haemoglobin (UK Biobank)	Prostate cancer (PRACTICAL)	Inverse variance weighted	287	0.016	0.032	0.629	992.021	286	1.15E-78	-0.048	0.079	0.984	1.049	0.924
Glycated haemoglobin (UK Biobank)	Prostate cancer (PRACTICAL)	Weighted median	287	-0.029	0.034	0.394	NR	NR	NR	-0.096	0.038	1.029	1.100	0.963
Glycated haemoglobin (UK Biobank)	Prostate cancer (PRACTICAL)	Simple mode	287	-0.009	0.073	0.899	NR	NR	NR	-0.151	0.133	1.009	1.163	0.876
Glycated haemoglobin (UK Biobank)	Prostate cancer (PRACTICAL)	Weighted mode	287	-0.009	0.031	0.768	NR	NR	NR	-0.071	0.052	1.009	1.073	0.949
Glycated haemoglobin (MAGIC)	Prostate cancer (PRACTICAL)	Inverse variance weighted	91	0.004	0.019	0.816	272.549	90	2.98E-20	-0.033	0.042	0.996	1.034	0.959
Glycated haemoglobin (MAGIC)	Prostate cancer (PRACTICAL)	Weighted median	91	0.003	0.020	0.879	NR	NR	NR	-0.036	0.042	0.997	1.037	0.958
Glycated haemoglobin (MAGIC)	Prostate cancer (PRACTICAL)	Simple mode	91	0.034	0.038	0.384	NR	NR	NR	-0.042	0.109	0.967	1.043	0.897
Glycated haemoglobin (MAGIC)	Prostate cancer (PRACTICAL)	Weighted mode	91	0.006	0.022	0.791	NR	NR	NR	-0.038	0.050	0.994	1.039	0.951
Glycated haemoglobin without SGLT2 variant (UK Biobank)	Prostate cancer (PRACTICAL)	Inverse variance weighted	268	-0.007	0.024	0.778	813.157	267	1.58E-56	-0.054	0.041	1.007	1.056	0.960
Glycated haemoglobin without SGLT2 variant (UK Biobank)	Prostate cancer (PRACTICAL)	Weighted median	268	-0.021	0.027	0.446	NR	NR	NR	-0.074	0.033	1.021	1.077	0.968
Glycated haemoglobin without SGLT2 variant (UK Biobank)	Prostate cancer (PRACTICAL)	Simple mode	268	-0.023	0.060	0.700	NR	NR	NR	-0.141	0.094	1.023	1.151	0.910
Glycated haemoglobin without SGLT2 variant (UK Biobank)	Prostate cancer (PRACTICAL)	Weighted mode	268	-0.013	0.023	0.581	NR	NR	NR	-0.057	0.032	1.013	1.059	0.969

Supplementary Table 13B. Association of observed HbA1c (mmol/mol) with prostate cancer incidence in 165,430 men with European ancestry from UK Biobank. Related to STAR Methods

Cox model	Considering competing risk in the model	N cases/N control	HR (95% CI) per 1 increase in HbA1c
Crude	No	7986/157,444	1.02 (1.01, 1.02)
Crude	Yes (13,192 men dead due to other reasons before 2022-02-01)	7986/157,444	1.02 (1.01, 1.03)
Confounder-adjusted	No	7789/153,633	0.99 (0.99, 1.00)
Confounder-adjusted	Yes (12,498 men dead due to other reasons before 2022-02-01)	7789/153,633	0.99 (0.99, 1.00)

Note: age, BMI, smoking status, alcohol consumption, physical activity and diet score were adjusted as covariates in the Cox model.

Supplementary Table 13C. Association of baseline type 2 diabetes with prostate cancer incidence in men with East Asian ancestry from 4C. Related to STAR Methods

	NGR	IGR	T2DM
Events	36	103	74
Incidence rate (per 1000 person-year)	0.32 (0.23-0.45)	0.36 (0.30-0.44)	0.47 (0.37-0.60)
Multivariable adjusted HR (95%CI)	1.0 (reference)	0.93 (0.61-1.42)	1.13 (0.72-1.75)
Multivariable adjusted HR (95%CI)*	1.0 (reference)	0.92 (0.61-1.40)	1.09 (0.70-1.70)

Note: NGR, normal glucose regulation; IGR, impaired glucose regulation; T2DM, Type 2 diabetes mellitus. Adjusted for age, gender, body-mass index, family history of diabetes, smoking, drinking, high school or above education, moderate or vigorous physical activity, diet score, systolic blood pressure, LDL-cholesterol. *Excluding prostate cancers occur during the first year.

Supplementary Table 14. MELODI Preso results to identify potential intermediate traits that linking SGLT2 inhibitors with prostate cancer. Related to STAR Methods

X Subject	X Pval	X Predicate	Overlap	Y Predicate	Y Object	Y Pval	Y
Sodium-Glucose Transporter 2 Inhibitors	0.00E+00	TREATS	Obesity	AUGMENTS	Urinary Incontinence	#######	prostate_cancer
dapagliflozin	0.00E+00	TREATS	Obesity	AUGMENTS	Urinary Incontinence	#######	prostate_cancer
dapagliflozin	6.80E-09	STIMULATES	FRAP1 protein, human|MTOR	COEXISTS_WITH	FLVCR1	#######	prostate_cancer
canagliflozin	6.80E-09	STIMULATES	Heme Oxygenase-1	COEXISTS_WITH	FLVCR1	#######	prostate_cancer
canagliflozin	6.80E-09	STIMULATES	Heme Oxygenase-1	INTERACTS_WIT	FLVCR1	#######	prostate_cancer
canagliflozin	6.80E-09	STIMULATES	Heme Oxygenase-1	STIMULATES	VEGF protein, human|VEGFA	#######	prostate_cancer
Licogliflozin	6.80E-09	TREATS	Obesity	AUGMENTS	Urinary Incontinence	#######	prostate_cancer
ertugliflozin	6.80E-09	TREATS	Obesity	AUGMENTS	Urinary Incontinence	#######	prostate_cancer
empagliflozin	2.92E-06	INTERACTS_WIT	cytokine	STIMULATES	Androgen Receptor|AR	#######	prostate_cancer
empagliflozin	2.92E-06	STIMULATES	Heme Oxygenase-1	COEXISTS_WITH	FLVCR1	#######	prostate_cancer
empagliflozin	2.92E-06	STIMULATES	Heme Oxygenase-1	INTERACTS_WIT	FLVCR1	#######	prostate_cancer
dapagliflozin	2.92E-06	TREATS	Coronary Arteriosclerosis	CAUSES	Congestive heart failure	#######	prostate_cancer
Sodium-Glucose Transporter 2 Inhibitors	2.92E-06	COEXISTS_WITH	Insulin	STIMULATES	Mitogen Activated Protein Kinase 1	#######	prostate_cancer
Sodium-Glucose Transporter 2 Inhibitors	2.92E-06	INHIBITS	Insulin	STIMULATES	Mitogen Activated Protein Kinase 1	#######	prostate_cancer
dapagliflozin	2.92E-06	COEXISTS_WITH	Insulin	STIMULATES	Mitogen Activated Protein Kinase 1	#######	prostate_cancer
empagliflozin	2.92E-06	STIMULATES	Heme Oxygenase-1	STIMULATES	VEGF protein, human|VEGFA	#######	prostate_cancer
canagliflozin	2.92E-06	TREATS	Obesity	AUGMENTS	Urinary Incontinence	#######	prostate_cancer
empagliflozin	2.92E-06	INTERACTS_WIT	cytokine	COEXISTS_WITH	FLVCR1	#######	prostate_cancer
