# Supplementary material for: The effect of SGLT2 inhibition on prostate cancer: Mendelian randomization and observational analysis using electronic healthcare and cohort data
Source: Cell Rep Med. 2024 Aug 20;5(8):101688. doi: 10.1016/j.xcrm.2024.101688 (PMC11384955; doi:10.1016/j.xcrm.2024.101688)
Supplement: Data S1. The STROBE-MR checklist of the current study, related to STAR Methods [file mmc3.pdf]

Data S1: The STROBE-MR check list of the current study, related to STAR Method.

Item	Complete/location
**1. Title and Abstract:** "Mendelian randomization" is named both in the title and the abstract	Mendelian randomization been added in the title and abstract.
**Introduction**	
**2. Background:** Explain the scientific background and rationale for the reported study. Is causality between exposure and outcome plausible? Justify why MR is a helpful method to address the study question.	Concept of Mendelian randomization was explained in the third paragraph of the introduction. Specific request for drug target Mendelian randomization were explained in the third paragraph of the introduction.
**3. Objectives:** State specific objectives clearly, including pre-specified causal hypotheses (if any).	The causal question has been stated in the fourth paragraph of the introduction.
**Methods**	
**4. Study design and data sources:** Present key elements of study design early in the paper. Consider including a table listing sources of data for all phases of the study. For each data source contributing to the analysis, describe the following:<br>a) Describe the study design and the underlying population from which it was drawn. Describe also the setting, locations, and relevant dates, including periods of recruitment, exposure, follow-up, and data collection, if available.<br>b) Give the eligibility criteria, and the sources and methods of selection of participants.<br>c) Explain how the analyzed sample size was arrived at.<br>d) Describe measurement, quality and selection of genetic variants.<br>e) For each exposure, outcome and other relevant variables, describe methods of assessment and, in the case of diseases, the diagnostic criteria used.<br>f) Provide details of ethics committee approval and participant informed consent, if relevant.	All necessary information about the GWAS studies been used in this study have been described in the method section and eTable 4.<br><br>The genetic predictor selection process has been described in in the Methods section "Genetic instrument selection for SGLT2 inhibition and HbA1c" and in eNote 1.<br><br>Ethics approval and informed consent info in the " Study Design and Data sources" section of the method section.
**5. Assumptions:** Explicitly state assumptions for the main analysis (e.g. relevance, exclusion, independence, homogeneity) as well assumptions for any additional or sensitivity analysis.	The Mendelian randomization assumptions have been described in method section "Validation of MR assumptions".
**6. Statistical methods main analysis** Describe statistical methods and statistics used.<br>a) Describe how quantitative variables were handled in the analyses (i.e., scale, units, model).<br>b) Describe the process for identifying genetic variants and weights to be included in the	(a) Described in the methods section, eNote 1 and Figure 1, 2.<br>b) Described in the Methods section " Genetic instrument selection for SGLT2 inhibition and HbA1c". The flow chart been provided in Figure 1.<br>c) Described in the section " Statistical analyses" within Methods.

analyses (i.e, independence and model). Consider a flow diagram.<br>c) Describe the MR estimator, e.g. two-stage least squares, Wald ratio, and related statistics.<br>Detail the included covariates and, in case of two-sample MR, whether the same covariate set was used for adjustment in the two samples.<br>d) Explain how missing data were addressed.<br>e) If applicable, say how multiple testing was dealt with.	d) Described in the section " Statistical analyses" within Methods.<br>e) Described in the section " Validation of MR assumptions" within Methods.
**7. Assessment of assumptions: Describe any methods used to assess the assumptions or justify their validity.**	We have drafted a specific section "Validation of MR assumptions" in the Methods, which explained how we deal with each of the Mendelian randomization assumption in this study.
**8. Sensitivity analyses:** Describe any sensitivity analyses or additional analyses performed.	The Mendelian randomization sensitivity analyses have been listed in "Validation of MR assumptions" section of the Method and in Supplementary Note 2.
**9. Software and pre-registration**<br><br>a) Name statistical software and package(s), including version and settings used.<br>b) State whether the study protocol and details were pre-registered (as well as when and where).	a) All statistical software and settings used are described in the "Validation of MR assumptions" section.<br>b) The analysis plan was described in the " Study design and data sources" section of the Methods and Figure 1.
**Results**	
**10. Descriptive data**<br>a) Report the numbers of individuals at each stage of included studies and reasons for exclusion. Consider use of a flow-diagram.<br>b) Report summary statistics for phenotypic exposure(s), outcome(s) and other relevant variables (e.g. means, standard deviations, proportions).<br>c) If the data sources include meta-analyses of previous studies, provide the number of studies, their reported ancestry, if available, and assessments of heterogeneity across these studies. Consider using a supplementary table for each data source.<br>d) For two-sample Mendelian randomization:<br>i. Provide information on the similarity of the genetic variant-exposure associations between the exposure and outcome samples.<br>ii. Provide information on extent of sample overlap between the exposure and outcome data sources.	a) Information is given in Figure 1 and Figure 3A.<br>b) We listed the detailed information of the summary statistics for our instruments in Table 1 and eTable S1-3. Summary statistics are in eTable 4.<br>c) We give this information in eTable 4.<br>d) We provide this information in eNote 1.
**11. Main results**<br><br>a) Report the associations between genetic variant and exposure, and between genetic variant and outcome, preferably on an interpretable scale (e.g.	a) Genetic exposure associations have been reported in eTable 1-3.<br>b) The causal effect estimates between exposures and outcomes were listed in Figure 2, eFigure 2 and 3; Table 2; and eTable 6, 7, 9 and 10.

comparing 25th and 75th percentile of allele count or genetic risk score, if individual-level data available).<br>b) Report causal effect estimate between exposure and outcome, and the measures of uncertainty from the MR analysis. Use an intuitive scale, such as odds ratio, or relative<br>risk, per standard deviation difference.<br>c) If relevant, consider translating estimates of relative risk into absolute risk for a meaningful time-period.<br>d) Consider any plots to visualize results (e.g. forest plot, scatterplot of associations between genetic variants and outcome versus between genetic variants and exposure).	Our results were presented in terms of odds ratio and confidence intervals throughout the results section for binary outcomes.<br>d) We visualize results using three sets of forest plot in Figure 2 and eFigure 3.
**12. Assessment of assumptions**<br><br>a) Assess the validity of the assumptions.<br>b) Report any additional statistics (e.g., assessments of heterogeneity, such as I2, Q statistic).	a) We assess the validity using sensitivity analyses, generalized inverse variance weighted, weighted median approach and mode estimate approach and signal variant Mendelian randomization approach. Results were presented in the Results section.<br>b) We discuss the use of Cochran's Q and Rucker's Q in the Results.
**13. Sensitivity and additional analyses**<br>a) Use sensitivity analyses to assess the robustness of the main results to violations of the assumptions.<br>b) Report results from other sensitivity analyses (e.g., replication study with different dataset, analyses of subgroups, validation of instrument(s), simulations, etc.).<br>c) Report any assessment of direction of causality (e.g., bidirectional MR).<br>d) When relevant, report and compare with estimates from non-MR analyses.<br>e) Consider any additional plots to visualize results (e.g., leave-one-out analyses).	a) we reported the use of genetic colocalization as additional approach to test for Mendelian randomization for the causal gene analysis of SGLT2 on prostate cancer.<br>b) validation of instruments was reported in the first paragraph of the Results section.
**Discussion**	
**14. Key results**	Discussion paragraph 1
**15. Limitations**<br>Discuss limitations of the study, taking into account the validity of the MR assumptions, other sources of potential bias, and imprecision. Discuss both direction and magnitude of any potential bias, and any efforts to address them.	Discussion paragraph 5
**16. Interpretations**<br>a) Give a cautious overall interpretation of results considering objectives and limitations.<br>Compare with results from other relevant studies.<br>b) Discuss underlying biological mechanisms that could be modelled by using the genetic	a) Interpretation: Discussion paragraphs 1, 2, 3, 4; Comparison with other studies: Discussion paragraphs 2, 3, 4.<br>b) Discussion paragraph 3<br>c) Discussion paragraph 1, 2, 3, 4.

variants to assess the relationship between the exposure and the outcome.<br>c) Discuss whether the results have clinical or policy relevance, and whether interventions<br>could have the same size effect.	
**17. Generalizability:**	We have discussed the potential caveats in terms of generalizability of our findings in the 4th and 5th paragraph of the Discussion section.
**18. Funding:**	We have reported all sources of funding in the "Acknowledgements" section.
**19. Data and data sharing:**	We have provided the link/approach to access genetic data used in this study in the "Data and materials availability" section. The software and scripts been used in this study was listed in the same section.
**20. Conflicts of Interest:**	All authors have declared conflicts of interest (none reported).
