# Supplementary material for: The effect of SGLT2 inhibition on prostate cancer: Mendelian randomization and observational analysis using electronic healthcare and cohort data
Source: Cell Rep Med. 2024 Aug 20;5(8):101688. doi: 10.1016/j.xcrm.2024.101688 (PMC11384955; doi:10.1016/j.xcrm.2024.101688)
Supplement: Document S2. Article plus supplemental information [file mmc4.pdf]

Cell Reports Medicine

Article

The effect of SGLT2 inhibition on prostate cancer: Mendelian randomization and observational analysis using electronic healthcare and cohort data

Graphical abstract

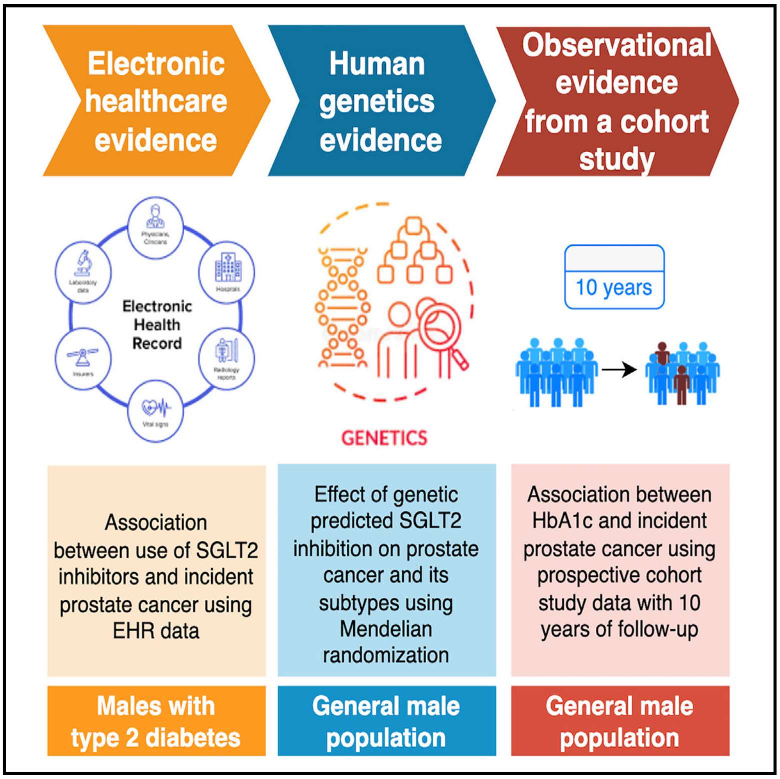

Authors

Jie Zheng, Jieli Lu, Jiying Qi, ..., Bin Cui, Weiqing Wang, Yufang Bi

Correspondence

zj12477@rjh.com.cn (J.Z.),
richard.martin@bristol.ac.uk (R.M.M.),
wqingw61@163.com (W.W.),
byf10784@rjh.com.cn (Y.B.)

In brief

Zheng et al. combined genetic, real-world, and cohort evidence to show a causal protective effect of SGLT2 inhibition on the risk of prostate cancer and showed that this effect is likely through a non-glycemic pathway. This study prioritized the prescription of SGLT2 inhibitors for those with prostate cancer risk.

Highlights

- A causal protective effect of SGLT2 inhibition on the risk of prostate cancer was observed
- This is likely to be a non-glycemic effect of SGLT2 inhibition on prostate cancer
- The prescription of SGLT2 inhibitors was prioritized for those with prostate cancer risk

Zheng et al., 2024, Cell Reports Medicine 5, 101688
August 20, 2024 © 2024 The Authors. Published by Elsevier Inc.
https://doi.org/10.1016/j.xcrm.2024.101688

CellPress

Cell Reports Medicine

CellPress
OPEN ACCESS

Article

The effect of SGLT2 inhibition on prostate cancer: Mendelian randomization and observational analysis using electronic healthcare and cohort data

Jie Zheng,[1,2,3,18,20,*] Jieli Lu,[1,2,18] Jiying Qi,[1,2] Qian Yang,[3] Huiling Zhao,[3] Haoyu Liu,[1,2] Zhihe Chen,[1,2] Lanhui Huang,[1,2] Youqiong Ye,[4,5] Min Xu,[1,2] Yu Xu,[1,2] Tiange Wang,[1,2] Mian Li,[1,2] Zhiyun Zhao,[1,2] Ruizhi Zheng,[1,2] Shuangyuan Wang,[1,2] Hong Lin,[1,2] Chunyan Hu,[1,2] Celine Sze Ling Chui,[6,7,8] Shiu Lun Au Yeung,[7] Shan Luo,[7] Olympia Dimopoulou,[3,9] Padraig Dixon,[3,10] Sean Harrison,[3,10] Yi Liu,[3] Jamie Robinson,[3] James Yarmolinsky,[3] Philip Haycock,[3] Jinqiu Yuan,[12,13,14,15] Sarah Lewis,[3] Zhongshang Yuan,[16] Tom R. Gaunt,[3,11,17] George Davey Smith,[3,11,17] Guang Ning,[1,2] Richard M. Martin,[3,7,17,19,*] Bin Cui,[1,2] Weiqing Wang,[1,2,19,*] and Yufang Bi[1,2,19,*]

[1]Department of Endocrine and Metabolic Diseases, Shanghai Institute of Endocrine and Metabolic Diseases, Ruijin Hospital, Shanghai Jiao Tong University School of Medicine, Shanghai, China
[2]Shanghai National Clinical Research Center for metabolic Diseases, Key Laboratory for Endocrine and Metabolic Diseases of the National Health Commission of the PR China, Shanghai Key Laboratory for Endocrine Tumor, Shanghai Digital Medicine Innovation Center, Ruijin Hospital, Shanghai Jiao Tong University School of Medicine, Shanghai, China
[3]MRC Integrative Epidemiology Unit (IEU), Bristol Medical School, University of Bristol, Oakfield House, Oakfield Grove, Bristol BS8 2BN, UK
[4]Center for Immune-Related Diseases at Shanghai Institute of Immunology, Department of Gastroenterology, Ruijin Hospital, Shanghai Jiao Tong University School of Medicine, Shanghai, China
[5]Shanghai Institute of Immunology, State Key Laboratory of Oncogenes and Related Genes, Department of Immunology and Microbiology, Shanghai Jiao Tong University School of Medicine, Shanghai 200025, China
[6]School of Nursing, Li Ka Shing Faculty of Medicine, The University of Hong Kong, Hong Kong Special Administration Region, China
[7]School of Public Health, Li Ka Shing Faculty of Medicine, The University of Hong Kong, Hong Kong Special Administration Region, China
[8]Laboratory of Data Discovery for Health (D24H), Hong Kong Science Park, Hong Kong Science and Technology Park, Hong Kong Special Administration Region, China
[9]Population Health Sciences, Bristol Medical School, University of Bristol, Bristol BS8 2PS, UK
[10]Nuffield Department of Primary Care Health Sciences, University of Oxford, Oxford OX2 6GG, UK
[11]Department of Population Health Sciences, Bristol Medical School, University of Bristol, Bristol BS8 2BN, UK
[12]Clinical Research Center, The Seventh Affiliated Hospital, Sun Yat-sen University, Shenzhen, Guangdong 518107, China
[13]Center for Digestive Disease, The Seventh Affiliated Hospital, Sun Yat-sen University, Shenzhen, Guangdong 518107, China
[14]Guangzhou Women and Children Medical Center, Guangzhou, Guangdong 510623, China
[15]Division of Epidemiology, the JC School of Public Health & Primary Care, the Chinese University of Hong Kong, Hong Kong
[16]Department of Biostatistics, School of Public Health, Cheeloo College of Medicine, Shandong University, Jinan, China
[17]NIHR Biomedical Research Centre at the University Hospitals Bristol and Weston NHS Foundation Trust and the University of Bristol, Bristol, UK
[18]These authors contributed equally
[19]Senior author
[20]Lead contact
*Correspondence: zj12477@rjh.com.cn (J.Z.), richard.martin@bristol.ac.uk (R.M.M.), wqingw61@163.com (W.W.), byf10784@rjh.com.cn (Y.B.)
https://doi.org/10.1016/j.xcrm.2024.101688

SUMMARY

We evaluated the effect of sodium-glucose cotransporter 2 (SGLT2) inhibition on prostate cancer by evidence triangulation. Using Mendelian randomization, we found that genetically proxied SGLT2 inhibition reduced the risk of overall (odds ratio = 0.56, 95% confidence interval [CI] = 0.38 to 0.82; 79,148 prostate cancer cases and 61,106 controls), advanced, and early-onset prostate cancer. Using electronic healthcare data (n_{SGLT2i} = 24,155; n_{DPP4i} = 24,155), we found that the use of SGLT2 inhibitors was associated with a 23% reduced risk of prostate cancer (hazard ratio = 0.77, 95% CI = 0.61 to 0.99) in men with diabetes. Using data from two prospective cohorts (n_{4C} = 57,779; $n_{UK\_Biobank}$ = 165,430), we found little evidence to support the association of HbA_{1c} with prostate cancer, implying a non-glycemic effect of SGLT2 inhibition on prostate cancer. In summary, this study provides multiple layers of evidence to support the beneficial effect of SGLT2 inhibition on reducing prostate cancer risk. Future trials are warranted to investigate whether SGLT2 inhibitors can be recommended for prostate cancer prevention.

© 2024 The Authors. Published by Elsevier Inc.
This is an open access article under the CC BY license (http://creativecommons.org/licenses/by/4.0/).

INTRODUCTION

Diabetes is one of the most common chronic conditions, affecting 537 million individuals in 2021.[1] Among various types of anti-diabetic drugs, recent clinical trials have demonstrated the beneficial effect of sodium-glucose cotransporter 2 (SGLT2) inhibitors in reducing the risk of atherosclerotic cardiovascular disease (ASCVD) in addition to improvements in HbA_{1c}.[2–4] Based on the robust trial evidence, the American Diabetes Association and European Association for the Study of Diabetes guidelines have, since 2020, recommended SGLT2 inhibitors as first-line therapy for patients with or at high risk for ASCVD, heart failure, or chronic kidney disease.[5] It has now been widely used by clinicians from endocrinology and cardiology departments.

Cancer is recognized as a common comorbidity for type 2 diabetes mellitus (T2DM).[6] Among various cancer types, prostate cancer is the second most commonly diagnosed malignancy in men, with nearly 1.41 million new cases reported worldwide in 2020, and is a major cause of cancer death in men.[7] However, no clinical guideline recommends the use of anti-diabetic drugs for individuals with cancers or those at high risk of developing cancers, especially for males with both diabetes and prostate cancer. A recent review has summarized the anti-cancer mechanisms of SGLT2 inhibitors.[8] Observational studies have also reported a decreased risk of prostate cancer among men with diabetes who are taking SGLT2 inhibitors.[9] However, the largest meta-analysis of randomized controlled trials (RCTs) in individuals with T2DM suggested little difference in prostate cancer incidence between users of SGLT2 inhibitors and users of placebo or active comparators.[10] Notably, this study's statistical power might be limited due to the small number of incident prostate cancer cases (n = 41) included in the analysis. Collectively, existing epidemiology studies provide some clues, but the evidence supporting the protective effect of SGLT2 inhibition on prostate cancer risk remains insufficient. Whether SGLT2 inhibition can be recommended for diabetic individuals at high risk of cancers or potentially repurposed as an anti-cancer therapeutic target needs further investigation.

Evidence triangulation is the practice of obtaining more reliable answers to research questions through integrating results from several different methods.[11] These methods have different assumptions and unrelated sources of biases. If results of these methods point to a similar conclusion, this will strengthen confidence in the finding. For the causal question aimed at identifying the effect of a drug target on a disease, human genetics, electronic healthcare, and cohort data are commonly employed data sources.[12,13] Triangulating evidence from these methods in a single study may provide an attractive strategy to improve evidence level for drug repurposing. Mendelian randomization (MR) is a method that utilizes germline genetic variants as proxy measures of exposure to estimate the causal effect of an exposure on an outcome.[14] An individual's germline genetic makeup influences their biology from conception, meaning that causal estimates from MR studies reflect lifelong exposures (e.g., lifelong SGLT2 inhibition) and are generally not susceptible to reverse causation or confounding.[15] Observational associations regarding the use of a drug on disease incidence are normally estimated using Cox proportional hazard models, where a "new user active comparators" design may reduce the influence of confounders.[16] Prospective cohort studies provide observational associations between an exposure and an outcome, which may be influenced by confounding factors. Due to the availability of enriched data sources supporting the application of all three methods,[17,18] studying the effect of SGLT2 inhibition on prostate cancer serves as a preferred example for evidence triangulation.

The objective of this study was to estimate the causal effects of SGLT2 inhibition on prostate cancer and its subtypes by triangulating evidence from human genetics, electronic healthcare, and biological data. The effect of HbA_{1c} on prostate cancer was further estimated using human genetics and observational epidemiology approaches.

RESULTS

Summary of study design and data sources

Figure 1 presents an overview of three sets of analyses conducted in this study. Each analysis aims to answer the same causal question in different subpopulations. All studies contributing data to this analysis had the relevant institutional review board approval from each country, and all participants provided informed consent.

First, the association of the use of SGLT2 inhibitors with incident prostate cancer was estimated in diabetic individuals using data derived from electronic health record data in the Shanghai Link Healthcare Database (SLHD; n = 81,122 men with diabetes; Table S1), a representative clinical database covering electronic healthcare records for over 99% of Shanghai residents since 2013[19] (more details in the STAR Methods, expermential model and subject details).

Second, the human genetics analysis was applied in the general male population. We estimated the putative causal effects of SGLT2 inhibition and genetically predicted HbA_{1c} on the risks of prostate cancer and its subtypes using MR (Tables S2, S3, and S4; Figure S1). The summary genetic association data from a case-control genome-wide association study (GWAS) of prostate cancer in the PRACTICAL and GAME-ON/ELLIPSE Consortium[17,18] were used (n = 140,254 men from the general population; Table S5; more details in the STAR Methods, the PRACTICAL and GAME-ON/ELLIPSE Consortium). MR has three key assumptions (Figure S2): (1) the germline genetic instruments used to proxy SGLT2 inhibition are robustly associated with the exposure ("relevance"); (2) there is no confounding of the relationship between the instruments and the outcome ("independence"); and (3) the instruments are only associated with the outcome through the exposure under study ("exclusion restriction"). The validity of these assumptions was tested using a set of sensitivity analyses.

Third, the association of baseline HbA_{1c} levels with incident prostate cancer during 10 years of follow-up was estimated using data from the China Cardiometabolic and Cancer Cohort (4C) study[6] (n = 57,779 men from the general population; more details in the STAR Methods, the China Cardiometabolic and Cancer Cohort (4C) study) and UK Biobank (n = 165,430). Both human genetics and observational analyses were related to prostate cancer risk, which are related to disease prevention.

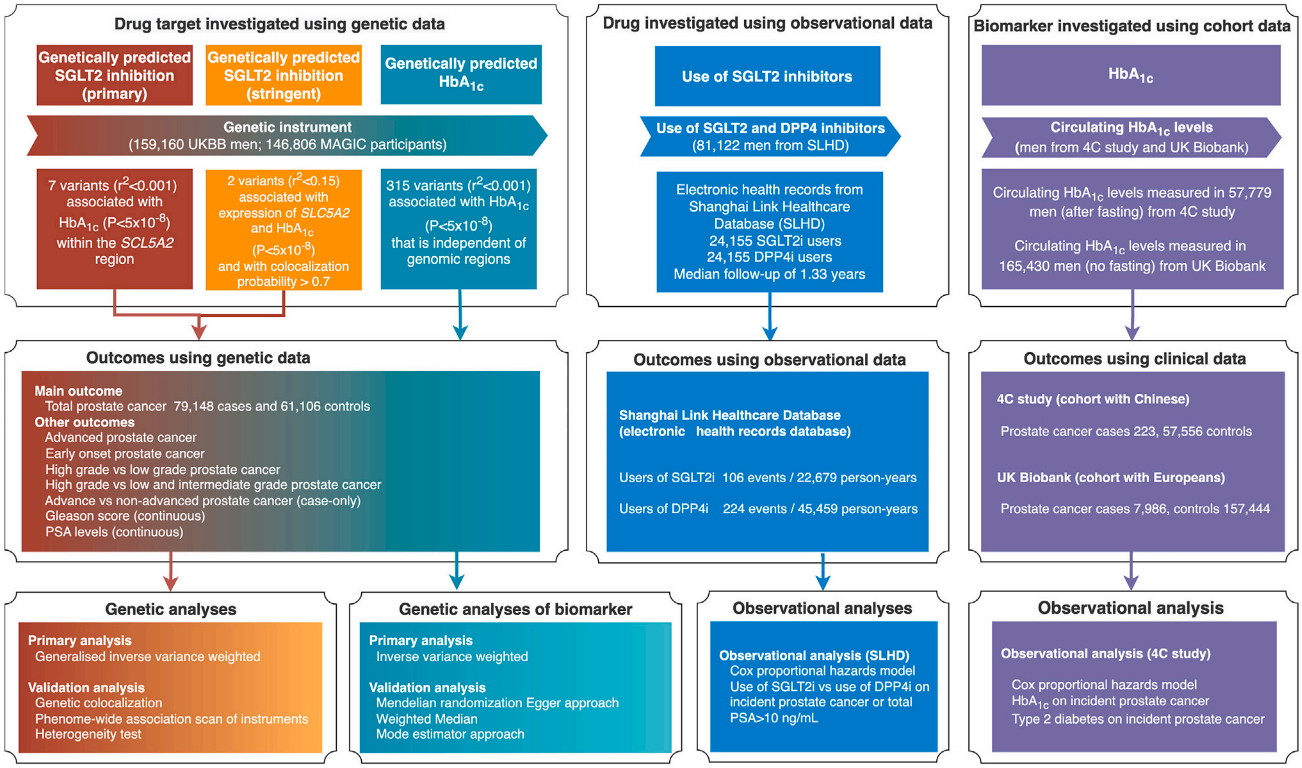

Figure 1. Genetic instrument selection, data sources, and analysis strategy in a triangulation study of the effect of SGLT2 inhibition on prostate cancer

For human genetic analyses, the effect of sodium-glucose cotransporter 2 (SGLT2) inhibition on the risk of prostate cancer and its subtypes were estimated using Mendelian randomization. For observational analyses, the effect of use of SGLT2 inhibitors on incident prostate cancer risk was estimated in males with diabetes. DPP4 inhibitors were used as active comparators. For observational analysis of biomarker, the association of HbA1c on incident prostate cancer was estimated in UK Biobank and 4C study. More details of instrument selection and analysis strategies were listed in the STAR Methods, instrument selection and Mendelian randomization analyses.

Effects of SGLT2 inhibition on prostate cancer risk

The characteristics of the primary and stringent genetic instruments used to proxy SGLT2 inhibition are listed in Tables 1, S2, S3, and S4, respectively. Across these exposures, the F-statistics used to test the relevance MR assumption suggested that weak instrument bias was unlikely to be an issue in this study (Figure S2).

Genetically proxied SGLT2 inhibition (estimated by primary instruments), equivalent to a one SD (0.62%) reduction in HbA$_{1c}$, reduced the risk of total prostate cancer by 44% (odds ratio [OR] = 0.56, 95% CI = 0.38 to 0.82, p = 0.003; Tables 2 and S6). This effect was consistent across the seven instruments (heterogeneity p = 0.80; Figure 2). The other four sensitivity MR models showed similar effect estimates (Figure S3).

Genetically proxied SGLT2 inhibition lowered the risk of advanced (OR = 0.52, 95% CI = 0.27 to 0.99; p = 0.049) and early-onset (OR = 0.27, 95% CI = 0.11 to 0.71; p = 0.008) prostate cancer. Little evidence was observed to support an effect of SGLT2 inhibition on other prostate-cancer-related outcomes (Table 2). In addition, there was little evidence to support an effect of SGLT2 inhibition on prostate-specific antigen (PSA) levels (β = −0.14, 95% CI = −0.30 to 0.03, p = 0.11; Table S6), which suggested that SGLT2 inhibition is likely to show an effect on reducing risk rather than influencing the diagnostic workup for prostate cancer. As a positive control, we confirmed the well-established effect of SGLT2 inhibition on reducing the risk of T2DM (OR = 0.66, 95% CI = 0.49 to 0.88, p = 0.005; Table S6).

The validation MR analysis using the two instruments selected by the stringent approach and using SGLT2 instruments derived from the MAGIC consortium validated the effect of SGLT2 inhibition on total, advanced, and advanced vs. localized prostate cancer (Figure 2; Table S7).

Tests of MR assumptions

The exchangeability MR assumption was tested using genetic colocalization between SGLT2 inhibition and prostate cancer (Figure S2), where we observed evidence of colocalization of the two traits in the *SLC5A2* region (colocalization probability = 72%; Table S8).

The exclusion restriction MR assumption was examined in several analyses (Figure S2). The phenome-wide association study (PheWAS) of the primary SGLT2 instruments showed that these genetic variants were associated with blood cell traits (e.g., red blood cell counts), body weight traits (e.g., waist circumference), diastolic blood pressure, and low-density lipoprotein cholesterol (Table S10). Multivariable MR adjusting for these traits, respectively (Table S10A), suggested that the effect

Table 1. Characteristics of genetic variants associated with HbA_{1c} (per 0.62% lowering) or expression levels of the *SLC5A2* gene and used as proxies for SGLT2 inhibition in the general population

Genetic variant	Gene	Effect allele/ non-effect allele	Effect allele frequency	Effect (95% CI)	*p* value
SGLT2 (primary)					
rs1232538	*SLC5A2*	G/T	0.73	−0.014 (−0.009 to −0.019)	4.0×10^{-8}
rs28675289	*SLC5A2*	T/C	0.04	−0.038 (−0.027 to −0.049)	1.5×10^{-11}
rs28692853	*SLC5A2*	A/C	0.50	−0.015 (−0.010 to −0.019)	2.8×10^{-10}
rs45625038	*SLC5A2*	C/T	0.97	−0.041 (−0.028 to −0.055)	1.2×10^{-9}
rs55766044	*SLC5A2*	C/T	0.72	−0.018 (−0.013 to −0.023)	3.9×10^{-12}
rs557720784	*SLC5A2*	C/T	0.95	−0.026 (−0.016 to −0.037)	6.1×10^{-7}
rs8050500	*SLC5A2*	C/T	0.45	−0.027 (−0.022 to −0.031)	1.2×10^{-30}
SGLT2 (stringent)					
rs9930811	*SLC5A2*	G/A	0.37	−0.016 (−0.021 to −0.012)	8.7×10^{-12}
rs35445454	*SLC5A2*	T/C	0.34	−0.013 (−0.018 to −0.008)	1.2×10^{-8}

Notation: two sets of instruments proxying SGLT2 inhibition using different instrument selection processes are listed here. For the main analysis, primary instruments selected genetic variants that were robustly associated with HbA_{1c} ($p < 1 \times 10^{-6}$) in the *SLC5A2* region. Stringent instruments selected genetic variants that were associated with both expression of *SLC5A2* gene and HbA_{1c} levels and showed colocalization evidence between the two (colocalization probability > 0.7) in the *SLC5A2* region, which were used in the main analysis. Two pairs of primary and stringent instruments were in moderate LD (r^2 between rs9930811 and rs8050500 = 0.56, r^2 between rs35445454 and rs1232538 = 0.23), which suggested that the two different selection processes picked two shared genetic signals as instruments in this region.

of SGLT2 inhibition on prostate cancer was independent of these traits (Table S10B). We further tested the effect of SGLT2 inhibition on prostate cancer risk adjusted for T2DM using a multivariable MR model, and we found that the effect of SGLT2 inhibition on prostate cancer was independent of its effect on T2DM (Table S10B). In addition, the SGLT2 instruments showed associations with the expression of 17 genes excluding *SLC5A2*, with two genes being targets for existing drugs for coagulation and hemoglobinuria treatment. The 17 genes were not associated with glycemic traits or had an interaction with any anti-diabetic or anti-cancer drugs[20] (Table S11). The differential gene expression analysis further suggested that most of the 17 genes were not associated with prostate cancer, which further reduced their probability of being pleotripy.

The MR sensitivity analyses did not provide strong evidence of heterogeneity or pleiotropy for the effect of SGLT2 inhibition on prostate cancer, but the statistical power to clearly demonstrate this was low (Tables S6 and S7).

Association of usage of SGLT2 inhibitors with prostate cancer risk using electronic healthcare data

We identified 26,988 new users of SGLT2 inhibitors and 54,134 new users of DPP4 inhibitors who fulfilled the eligibility criteria out of 130,817 males from SLHD (Figure 3A). After a 1:1 propensity score matching, we identified a cohort of 48,310 patients (24,155 in each group) with well-balanced baseline characteristics (standardized mean differences less than 1.5%) between the two treatment groups (Table S1). Cox proportional hazards model showed that SGLT2 inhibitors use (compared with DPP4 inhibitors use) was associated with a 23% reduction in the risk of prostate cancer (SGLT2 inhibitors use = 467.4 versus DPP4 inhibitors use = 492.75 per 100,000 person-years; hazard ratio [HR] = 0.77, 95% CI = 0.61 to 0.99, $p = 0.03$) during a median follow-up of 1.33 years (Figure 3B). Sensitivity analyses lagging the outcome period between one and six months showed similar protective effects, albeit less precisely estimated (Table S12).

Validating the influence of glucose: MR and observational association of HbA_{1c} with prostate cancer

We estimated the association of HbA_{1c} with prostate cancer risk using MR and observational analyses, which aimed to investigate whether the effect of SGLT2 inhibition on prostate cancer is partly via lowering HbA_{1c} levels. Little evidence was observed to support the effect of genetically proxied HbA_{1c} on total prostate cancer risk (OR = 0.98, 95% CI = 0.92 to 1.05, $p = 0.63$; Table 3). Sensitivity MR analyses in which we removed variants within the *SLC5A2* region showed similar effects to those seen in our analyses of HbA_{1c} on prostate cancer (Table S13A). Observational analysis in the 4C study also provided little evidence to support the effect of baseline HbA_{1c} levels on incident prostate cancer after 10 years of follow-up (HR = 0.93, 95% CI = 0.80 to 1.10, $p = 0.40$); the findings barely change after excluding individuals using anti-diabetic drugs (Table 3). One additional observational analysis in 157,444 male participants from UK Biobank further confirmed the null association between HbA_{1c} and incident prostate cancer (Table S13B; Figure S4).

The existing literature primarily from individuals of European ancestry had reported a protective association between diabetes and prostate cancer, but the studies from the Chinese population appear to show less consistent results.[19,21–26] We therefore tested the observational association of T2DM on prostate cancer in the 4C study. This analysis using the 10-year follow-up data did not show any evidence to support a protective or risk-increasing effect between the two (Table S13C). The discrepancy in the findings may be attributable to factors such as the relatively small sample size and shorter follow-up duration in the 4C study.

Table 2. Effect estimates of genetically proxied SGLT2 inhibition on total, aggressive, and early-onset prostate cancer among men in general population using data from the PRACTICAL and GAME-ON/ELLIPSE Consortium

Exposure	Outcome	No. of cases	Model	Odds ratio (95% CI)	*p* value
Genetically proxied SGLT2 inhibition	total prostate cancer	79,148	inverse variance weighted MR	0.56 (0.38–0.82)	0.003
	advanced prostate cancer	15,167	inverse variance weighted MR	0.52 (0.27–0.99)	0.049
	early-onset prostate cancer	6,988	inverse variance weighted MR	0.27 (0.11–0.71)	0.008
	advanced vs. non-advanced	14,160	inverse variance weighted MR	0.86 (0.35–2.13)	0.75
	high vs. low aggressive	15,561	inverse variance weighted MR	1.14 (0.38–3.39)	0.81
	high vs. low + intermediate aggressive	20,658	inverse variance weighted MR	0.69 (0.37–1.28)	0.24

Notation: advanced prostate cancer was defined as metastatic disease or Gleason score (GS) $\geq$ 8 or PSA > 100 or prostate cancer death; early-onset refers to prostate cancer onset before age 55; low aggressive refers to T stage from the TNM staging $\leq$ T1, and GS $\leq$ 6, and PSA < 10; intermediate aggressive refers to T stage: T2, and GS = 7, and PSA 10$\sim$20; and high aggressive refers to T stage: T3/T4 or N1 or M1 or GS $\geq$ 8 or PSA > 20. Odds ratio means the reduced odds of prostate cancer risk per standard deviation unit (0.62%) reduction of HbA1c through SGLT2 inhibition.

To further identify the potential biological mechanisms of SGLT2 inhibitors on prostate cancer, we applied MELODI Presto[27] to identify potential mediators that can link SGLT2 inhibitors with prostate cancer. This analysis suggested that intermediated traits such as obesity, the mammalian target of rapamycin, heme oxygenase-1 (an antioxidant with anti-inflammatory properties),[28] and insulin are potential intermediate phenotypes that may inform the non-glycemic mediators of SGLT2 inhibitors on prostate cancer (Table S14).

DISCUSSION

In this study, we triangulated human genetics, electronic healthcare, and prospective cohort evidence to answer the same causal question: the effect of SGLT2 inhibition on prostate cancer. In the genetic analysis, we observed that genetically proxied lifelong SGLT2 inhibition reduced total, advanced, and early-onset prostate cancer in the general male population by 44%, 48%, and 73%, respectively.

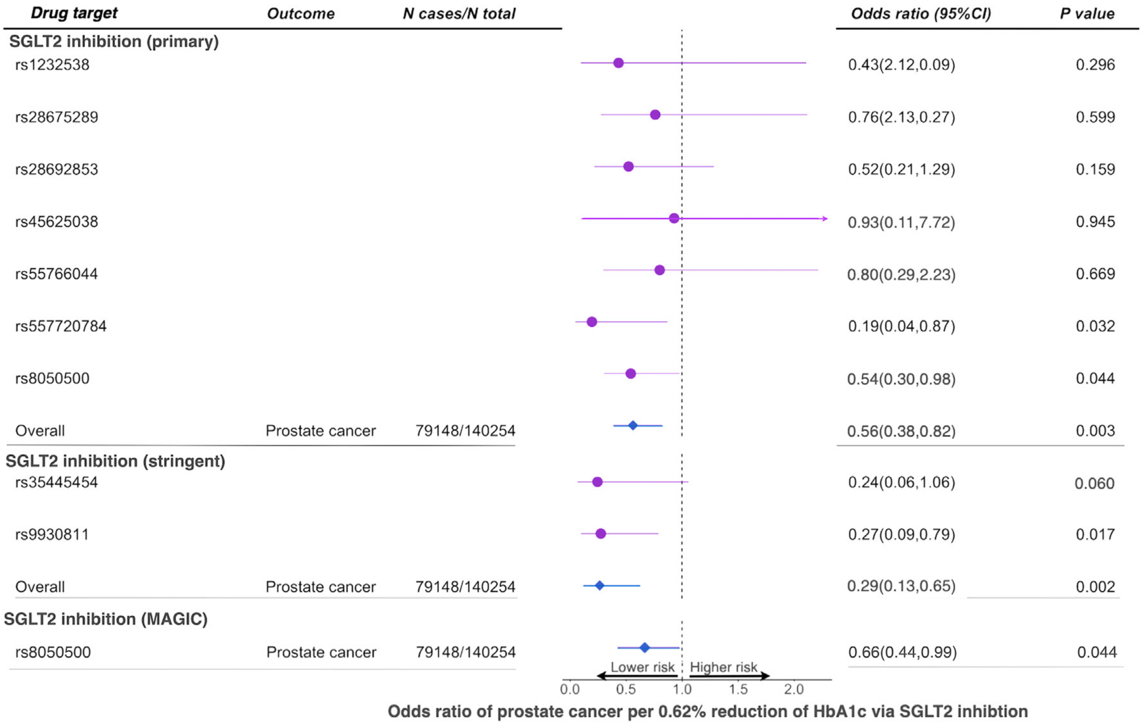

Figure 2. Mendelian randomization estimates of the effects of SGLT2 inhibition on prostate cancer risk in the general European population
Two sets of genetic instruments were used in this analysis. Primary instruments included seven genetic variants that were associated with HbA_{1c} ($p < 1 \times 10^{-6}$) in the *SLC5A2* region. Stringent instruments were two genetic variants associated with both expression levels of *SLC5A2* and HbA_{1c} levels (with colocalization probability >0.7 between the two) in the *SLC5A2* region. Odds ratio means the reduced odds of prostate cancer risk per standard deviation unit (0.62%) reduction of HbA1c through SGLT2 inhibition.

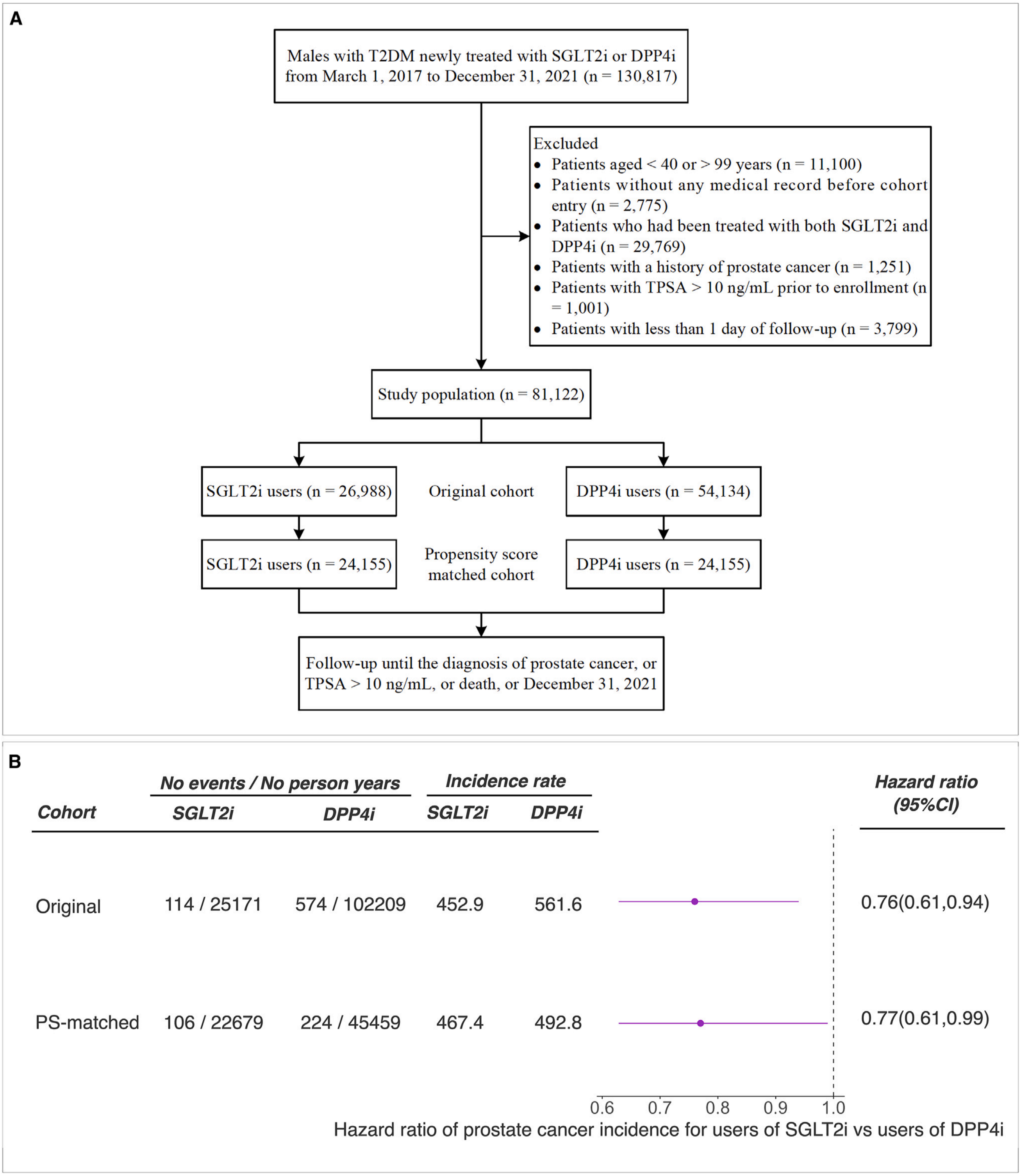

Figure 3. Flowchart of patient inclusion and association between the use of SGLT2 inhibitors and the risk of incident prostate cancer or being at high risk of prostate cancer

(A) Flowchart of patient inclusion in the study population. SGLT2i, sodium glucose cotransporter 2 inhibitors; DPP4i, dipeptidylpeptidase 4 inhibitors; TPSA, total prostate-specific antigen. A patient could be excluded for more than one reason.

(B) The association between use of SGLT2 inhibitors compared with DPP4 inhibitors and risk of prostate cancer or with total PSA > 10 ng/mL (which indicated high risk of prostate cancer). The covariates used in this analysis include demographic data (age), comorbidities (benign prostatic hyperplasia, hypertension,

(legend continued on next page)

Validation using various selection processes and datasets confirmed the protective effect of SGLT2 inhibition on the risk of prostate cancer and its subtypes, rather than an effect on PSA biasing the diagnosis of prostate cancer. In the validation using electronic healthcare data, we showed that SGLT2 inhibitor use reduced the risk of prostate cancer by 23% in men with T2DM. In the analyses validating the influence of glucose, we found little genetic and observational evidence to support an association of HbA_{1c} with prostate cancer, which implies a possible non-glucose mechanism of SGLT2 inhibition on prostate cancer prevention. Correctively, we provided three strands of evidence to prioritize SGLT2 inhibition as a target for prostate cancer prevention.

According to the US Centers for Disease Control and Prevention, adults aged between 45 and 64 receive the greatest number of new diagnoses of diabetes, which was also the age group that men are likely to receive diagnoses of prostate cancer. However, there was little evidence to support the setting up of clinical guidelines concerning the modification of SGLT2 inhibitor treatment among diabetic patients with co-existing or high-risk prostate cancer until now. A small number of observational studies supported the protective role of SGLT2 inhibitors on prostate cancer risk.[9] A recent systematic review of RCTs provided weak evidence of an effect of SGLT2 inhibitors on cancers.[29] Only one phase 1 trial was registered in ClinicalTrials.gov (NCT04887935), which aims to investigate the safety of dapagliflozin, one type of SGLT2 inhibitor, for men considered at high risk of prostate cancer. In the present study, we observed robust human genetics and electronic healthcare evidence to support the effect of SGLT2 inhibition on reducing the risk of prostate cancer, both in the general male population and in males with diabetes. Our results further support that SGLT2 inhibition may have better efficacy on the prevention of early-onset prostate cancer than on total and advanced prostate cancer. Our evidence supports the prioritization of future clinical trials of SGLT2 inhibitors in diabetic men at high risk of prostate cancer, which may have the potential to influence clinical guidelines/standards for diabetes.

It has been hypothesized that the primary mechanism of a beneficial effect of SGLT2 inhibitors on cancer is through inhibiting glycolysis in tumor cells, thus reducing tumor cell proliferation and tumorigenesis.[30] Another study showed that canagliflozin, one type of SGLT2 inhibitor, inhibits mitochondrial complex-I and cellular proliferation in prostate cancer cells.[31] However, the lack of MR and observational evidence of a role for HbA_{1c}[32] suggests that HbA_{1c} may not be driving the observed association of SGLT2 inhibition with prostate cancer. Correctively, our genetic evidence implies that SGLT2 inhibition may have a direct effect on prostate cancer prevention, which could be independent to its glucose control effect. Some well-designed clinical trials have also provided evidence to support that SGLT2 inhibitors have good tolerance and safety profiles to be used in individuals without diabetes.[33] Further functional and clinical studies are warranted to better understand the anti-cancer mechanism of SGLT2 inhibitors and test their anti-prostate cancer efficacy in individuals without diabetes.

Our study has several strengths. First, we estimated the effects of SGLT2 inhibition on prostate cancer prevention using genetic, electronic healthcare, and epidemiological approaches, which have different assumptions, key source of biases (e.g., pleiotropy for MR and confounders for observational analysis),[11] and different subgroup of population (i.e., the general male population and males with diabetes). Triangulation of evidence suggests that SGLT2 inhibition is likely to have a protective effect on prostate cancer in all subpopulation groups, which strengthens confidence in this finding. Second, the instruments for SGLT2 were selected using two widely applied pipelines.[32] The reliability of these instruments has been tested thoroughly in this study. Third, we paid special attention to the potential influence of our genetic variant-exposure estimates on our MR results and only used male-specific instruments in this study. Fourth, the results from colocalization analysis, PheWAS, multivariable MR, and other sensitivity MR analyses suggested that the effect of SGLT2 inhibition on prostate cancer is unlikely to violate the exchangeability and the exclusion restriction assumptions of MR. More interestingly, we extended the scope of differential gene expression analysis to distinguish pleiotropy from causality, and the strategy can be widely applied to other drug target genes and complex diseases.

Limitations of the study

This study has several limitations. First, our MR estimates of the effect of SGLT2 inhibition were scaled to represent the on-target reductions in HbA_{1c} levels rather than the direct effect of SGLT2 inhibitors. This assumes that SGLT2 inhibition has a proportional impact on lowering of HbA_{1c}. Second, caution is needed to interpret the causal effect estimate from this study. This is because the MR estimate reflects the long-term modulation of drug targets on disease risk, which may suggest different levels of risk reductions per unit change in drug target compared with those observed from clinical trials/observational studies over a relatively short duration, which would explain the attenuated effect estimate of our observational analysis. Furthermore, the estimated effect of SGLT2 inhibition on prostate cancer could at least in part be influenced by different ancestries, disease status, and survival bias, given the relatively late age-at-onset of prostate cancer. Third, the MR analyses presented assume no gene-environment interaction in the association of genetic proxies for drug targets and prostate cancer. Fourth, SGLT2 inhibitors have been marketed in China since March 2017; the median follow-up time for the observational analysis was therefore only 1.33 years. Therefore, we consider this result as a validation for evidence triangulation rather than a stand-alone finding. Fifth, due to lack of data in the SLHD database, we were not able to include socioeconomic status, family history of

dyslipidemia, diabetic complications, ischemic heart disease, peripheral vascular disease, heart failure, cerebrovascular disease, chronic lung disease, moderate or severe kidney disease, moderate or severe liver disease, and other cancers), anti-diabetic drugs (metformin, insulin, glucagon-like peptide-1 receptor agonist, sulfonylurea, glinide, α-glucosidase inhibitor, and thiazolidinedione), and other medications (angiotensin-converting enzyme inhibitor, angiotensin receptor blocker, calcium channel blocker, α/β-blockers, diuretic, statin, fibrate, aspirin, other antiplatelet drugs, non-steroidal anti-inflammatory drug, and 5α-reductase inhibitor). The unit of the incidence rate was 100,000 person-years. Harzard ratio is the probability of occurrence of prostate cancer in SGLT2 inhibitor users versus that in DPP4 ihibitor users during the follow-up period.

Table 3. Effect estimates of genetically proxied HbA_{1c} levels on total, aggressive, and early-onset prostate cancer among men in the general population using data from the PRACTICAL Consortium and association of observed HbA_{1c} levels on incident prostate cancer among men in the general population using data from the 4C study

Exposure	Outcome	No. of cases	Model	Odds ratio (95% CI)	Hazard ratio (95% CI)	*p* value
Genetically proxied HbA_{1c} levels	total prostate cancer	79,148	inverse variance weighted MR	0.98 (0.92–1.05)	–	0.63
	aggressive prostate cancer	15,167	inverse variance weighted MR	0.99 (0.92–1.07)	–	0.81
	early-onset prostate cancer	6,988	inverse variance weighted MR	0.94 (0.82–1.08)	–	0.37
Observed HbA_{1c} levels (one SD unit = 1.11%)	incident prostate cancer (including all 57,779 males)	223	Cox proportional hazard model	–	0.93 (0.80–1.10)	0.40
Observed HbA_{1c} levels (one SD unit = 0.91%)	incident prostate cancer (excluding users of anti-diabetic drugs)	201	Cox proportional hazard model	–	0.95 (0.80–1.12)	0.53

Notation: aggressive prostate cancer, defined as Gleason score $\geq$ 8, PSA > 100 ng/mL, metastatic disease (M1), or death from prostate cancer, and early-onset prostate cancer, defined as participants diagnosed with prostate cancer before the age of 55 years. SD refers to standard deviation. Odds ratio is the reduced odds of prostate cancer per standard deviation unit reduction of HbA1c levels (0.62%). Hazard ratio is the probability of occurence of prostate cancer in SGLT2 inhibitor users versus that in DPP4 inhibitor users during the follow-up period.

diseases, and lifestyle factors into the regression model, which may introduce confounding and bias the results. Finally, it is important to notice that the observational analyses using electronic healthcare records were mainly conducted in East Asian participants, while the genetic analysis was conducted only using GWAS of European ancestry. Given variation in the prevalence of prostate cancer across ancestries,[34] such ancestry disparities may influence the interpretation of the results. Therefore, we refrain from interpreting our findings as indicating that SGLT2 inhibition exhibits a protective effect on prostate cancer in both ancestries.

Conclusion

Genetic, electronic healthcare, and epidemiological evidence with different assumptions and using different subpopulations support the role of SGLT2 inhibition in reducing prostate cancer risk. Further clinical trials should be prioritized to establish whether there is a similar effect with the long-term prescription of SGLT2 inhibitors, at what age chemoprevention/treatment would need to commence, whether high-risk men should be targeted, and the potential harms.

STAR★METHODS

Detailed methods are provided in the online version of this paper and include the following:

- KEY RESOURCES TABLE
- RESOURCE AVAILABLILITY
  - Lead contact
  - Materials availability
  - Data and code availability
- EXPERIMENTAL MODEL AND SUBJECT DETAILS
  - The PRACTICAL and GAME-ON/ELLIPSE consortium
  - The Shanghai Link Healthcare Database
  - The China Cardiometabolic and Cancer Cohort (4C) study
- METHOD DETAILS
  - Causal inference analyses using Mendelian randomization
  - Outcome selection for human genetics analysis
  - Mendelian randomization analyses
  - Observational analysis using electronic healthcare data
  - Validation using prospective cohort data with over 10 years of follow-up
  - The prospective association of HbA1c with incident prostate cancer in UK Biobank
- QUALIFICATION AND STATISTICAL ANALYSIS

SUPPLEMENTAL INFORMATION

Supplemental information can be found online at https://doi.org/10.1016/j.xcrm.2024.101688.

ACKNOWLEDGMENTS

J.Z. is supported by the Academy of Medical Sciences (AMS) Springboard Award, the Wellcome Trust, the Government Department of Business, Energy and Industrial Strategy (BEIS), the British Heart Foundation, and Diabetes UK (SBF006\1117). J.Z. is funded by the Vice-Chancellor Fellowship from the University of Bristol. This project was supported by grants from the National Key Research and Development Program of China (2022YFC2505203). J.L., M.X., G.N., and Y.B. are supported by the National Natural Science Foundation of China (82370810, 82170819, 82088102, 81970691, 81970728, 81930021, and 81941017) and Shanghai Clinical Research Center for Metabolic Disease (19MC1910100). J.L., M.X., Y.X., T.W., M.L., Z.Z., S.W., H.L., G.N., W.W., and Y.B. are members of the Innovative Research Team of High-level Local Universities in Shanghai. G.N. and Y.B. are supported by the Shanghai Shenkang Hospital Development Center (SHDC12019101, SHDC2020CR1001A, and SHDC2020CR3064B). G.D.S. and T.R.G. are supported by the UK Medical Research Council Integrative Epidemiology Unit (MC_UU_00032/03). R.M.M., S.H., and P.D. received support from a Cancer Research UK (C18281/A29019) program grant (the Integrative Cancer Epidemiology Grant). R.M.M., T.R.G., and G.D.S. are also supported by the NIHR Bristol Biomedical Research Centre, which is funded by the NIHR and is a partnership between University Hospitals Bristol and Weston NHS Foundation Trust and the University of Bristol. Department of Health and Social Care disclaimer: the views expressed are those of the author(s) and not necessarily those of the NHS, the NIHR, or the Department of Health and Social Care. J.Y. is supported by a Cancer Research UK Population Research Postdoctoral Fellowship (C68933/A28534). R.M.M. is a National Institute for Health Research Senior Investigator (NIHR202411). This research has been conducted using the UKB Resources under application number 15825.

We thank the individual patients who provided the sample that made data available; without them the study would not have been possible. We thank the PRACTICAL and GAME-ON/ELLIPSE consortia for providing key genetic association summary statistics data to support this study.

AUTHOR CONTRIBUTIONS

J.Z., G.N., R.M.M., W.W., and Y.B. designed the study, wrote the research plan, and interpreted the results. J.Z. undertook the main, replication and sensitivity MR analyses with feedback from Q.Y., O.D., J. Yarmolinsky, and J.R. B.C. and J.Q. collected data from the Shanghai Link Healthcare Database and conducted the survival and linear regression analyses. C.S.L.C., S.L.A.Y., S. Luo, and J. Yuan provided critical suggestions. The observational analysis in UK Biobank was conducted by Q.Y. J.Z. and J.L. wrote the first draft of the manuscript with critical comments and revision from M.X., Y.X., T.W., M.L., Z.Z., R.Z., S.W., H. Lin, C.H., C.S.L.C., S.L.A.Y., S. Luo, O.D., P.D., S.H., Y.L., J.R., J. Yarmolinsky, P.H., J. Yuan, S. Lewis, T.R.G., G.D.S., R.M.M., W.W., Y.B., and G.N. J.Z. is the guarantor.

DECLARATION OF INTERESTS

G.D.S. reports scientific advisory board membership for Relation Therapeutics and Insitro. UK Biobank has received ethical approval from the UK National Health Service's National Research Ethics Service (ref. 11/NW/0382). All other studies contributing data to this analysis had the relevant institutional review board approval from each country and all participants provided informed consent.

Received: February 1, 2024
Revised: June 29, 2024
Accepted: July 24, 2024
Published: August 20, 2024

REFERENCES

1. International Diabetes Federation. (2021). IDF Diabetes Atlas, 10th edition. https://diabetesatlas.org/atlas/tenth-edition/.
2. GBD 2016 Causes of Death Collaborators (2017). Global, regional, and national age-sex specific mortality for 264 causes of death, 1980-2016: a systematic analysis for the Global Burden of Disease Study 2016. Lancet *390*, 1151–1210.
3. Neal, B., Perkovic, V., Mahaffey, K.W., de Zeeuw, D., Fulcher, G., Erondu, N., Shaw, W., Law, G., Desai, M., and Matthews, D.R.; CANVAS Program Collaborative Group (2017). Canagliflozin and Cardiovascular and Renal Events in Type 2 Diabetes. N. Engl. J. Med. *377*, 644–657.
4. Wiviott, S.D., Raz, I., Bonaca, M.P., Mosenzon, O., Kato, E.T., Cahn, A., Silverman, M.G., Zelniker, T.A., Kuder, J.F., Murphy, S.A., et al. (2019). Dapagliflozin and Cardiovascular Outcomes in Type 2 Diabetes. N. Engl. J. Med. *380*, 347–357.
5. Buse, J.B., Wexler, D.J., Tsapas, A., Rossing, P., Mingrone, G., Mathieu, C., D'Alessio, D.A., and Davies, M.J. (2020). 2019 Update to: Management of Hyperglycemia in Type 2 Diabetes, 2018. A Consensus Report by the American Diabetes Association (ADA) and the European Association for the Study of Diabetes (EASD). Diabetes Care *43*, 487–493.
6. Lu, J., He, J., Xu, Y., Zheng, R., Zheng, J., Qin, G., Qin, Y., Chen, Y., Tang, X., et al. (2024). Association of social determinants, lifestyle, and metabolic factors with mortality in Chinese adults: A nationwide 10-year prospective cohort study. Cell Rep. Med. *24*, 101656.
7. Sung, H., Ferlay, J., Siegel, R.L., Laversanne, M., Soerjomataram, I., Jemal, A., and Bray, F. (2021). Global Cancer Statistics 2020: GLOBOCAN Estimates of Incidence and Mortality Worldwide for 36 Cancers in 185 Countries. CA A Cancer J. Clin. *71*, 209–249.
8. Dutka, M., Bobiński, R., Francuz, T., Garczorz, W., Zimmer, K., Ilczak, T., Ćwiertnia, M., and Hajduga, M.B. (2022). SGLT-2 Inhibitors in Cancer Treatment-Mechanisms of Action and Emerging New Perspectives. Cancers *14*, 5811. https://doi.org/10.3390/cancers14235811.
9. Murtola, T.J., Tammela, T.L.J., Lahtela, J., and Auvinen, A. (2008). Antidiabetic medication and prostate cancer risk: a population-based case-control study. Am. J. Epidemiol. *168*, 925–931.
10. Tang, H., Dai, Q., Shi, W., Zhai, S., Song, Y., and Han, J. (2017). SGLT2 inhibitors and risk of cancer in type 2 diabetes: a systematic review and meta-analysis of randomised controlled trials. Diabetologia *60*, 1862–1872.
11. Lawlor, D.A., Tilling, K., and Davey Smith, G. (2016). Triangulation in aetiological epidemiology. Int. J. Epidemiol. *45*, 1866–1886.
12. Holmes, M.V., Richardson, T.G., Ference, B.A., Davies, N.M., and Davey Smith, G. (2021). Integrating genomics with biomarkers and therapeutic targets to invigorate cardiovascular drug development. Nat. Rev. Cardiol. *18*, 435–453.
13. Soni, P.D., Hartman, H.E., Dess, R.T., Abugharib, A., Allen, S.G., Feng, F.Y., Zietman, A.L., Jagsi, R., Schipper, M.J., and Spratt, D.E. (2019). Comparison of Population-Based Observational Studies With Randomized Trials in Oncology. J. Clin. Oncol. *37*, 1209–1216.
14. Smith, G.D., and Ebrahim, S. (2003). Mendelian randomization': can genetic epidemiology contribute to understanding environmental determinants of disease? Int. J. Epidemiol. *32*, 1–22.
15. Sanderson, E., Glymour, M.M., Holmes, M.V., Kang, H., Morrison, J., Munafò, M.R., Palmer, T., Schooling, C.M., Wallace, C., Zhao, Q., and Davey Smith, G. (2022). Mendelian randomization. Nat. Rev. Methods Primers *2*, 6.
16. Lund, J.L., Richardson, D.B., and Stürmer, T. (2015). The active comparator, new user study design in pharmacoepidemiology: historical foundations and contemporary application. Curr. Epidemiol. Rep. *2*, 221–228.
17. Schumacher, F.R., Al Olama, A.A., Berndt, S.I., Benlloch, S., Ahmed, M., Saunders, E.J., Dadaev, T., Leongamornlert, D., Anokian, E., Cieza-Borrella, C., et al. (2018). Association analyses of more than 140,000 men identify 63 new prostate cancer susceptibility loci. Nat. Genet. *50*, 928–936.
18. Elsworth, B., Lyon, M., Alexander, T., Liu, Y., Matthews, P., Hallett, J., Bates, P., Palmer, T., Haberland, V., and Smith, G.D. (2020). The MRC IEU OpenGWAS data infrastructure. Preprint at bioRxiv. https://doi.org/10.1101/2020.08.10.244293.
19. Qi, J., He, P., Yao, H., Song, R., Ma, C., Cao, M., Cui, B., and Ning, G. (2019). Cancer risk among patients with type 2 diabetes: A real-world study in Shanghai, China. J. Diabetes *11*, 878–883.
20. Cotto, K.C., Wagner, A.H., Feng, Y.-Y., Kiwala, S., Coffman, A.C., Spies, G., Wollam, A., Spies, N.C., Griffith, O.L., and Griffith, M. (2018). DGIdb 3.0: a redesign and expansion of the drug-gene interaction database. Nucleic Acids Res. *46*, D1068–D1073.
21. Bansal, D., Bhansali, A., Kapil, G., Undela, K., and Tiwari, P. (2013). Type 2 diabetes and risk of prostate cancer: a meta-analysis of observational studies. Prostate Cancer Prostatic Dis. *16*, 151–158.
22. Jian Gang, P., Mo, L., Lu, Y., Runqi, L., and Xing, Z. (2015). Diabetes mellitus and the risk of prostate cancer: an update and cumulative meta-analysis. Endocr. Res. *40*, 54–61.
23. Amadou, A., Freisling, H., Jenab, M., Tsilidis, K.K., Trichopoulou, A., Boffetta, P., Van Guelpen, B., Mokoroa, O., Wilsgaard, T., Kee, F., et al. (2021). Prevalent diabetes and risk of total, colorectal, prostate and breast cancers in an ageing population: meta-analysis of individual participant data from cohorts of the CHANCES consortium. Br. J. Cancer *124*, 1882–1890.
24. Peila, R., and Rohan, T.E. (2020). Diabetes, Glycated Hemoglobin, and Risk of Cancer in the UK Biobank Study. Cancer Epidemiol. Biomarkers Prev. *29*, 1107–1119.
25. Zaccardi, F., Ling, S., Brown, K., Davies, M., and Khunti, K. (2023). Duration of Type 2 Diabetes and Incidence of Cancer: An Observational Study in England. Diabetes Care *46*, 1923–1930.

26. Laurberg, T., Witte, D.R., Gudbjörnsdottir, S., Eliasson, B., and Bjerg, L. (2024). Diabetes-related risk factors and survival among individuals with type 2 diabetes and breast, lung, colorectal, or prostate cancer. Sci. Rep. *14*, 10956.

27. Elsworth, B., and Gaunt, T.R. (2021). MELODI Presto: A fast and agile tool to explore semantic triples derived from biomedical literature. Bioinformatics *37*, 583–585. https://doi.org/10.1093/bioinformatics/btaa726.

28. Campbell, N.K., Fitzgerald, H.K., and Dunne, A. (2021). Regulation of inflammation by the antioxidant haem oxygenase 1. Nat. Rev. Immunol. *21*, 411–425.

29. Marilly, E., Cottin, J., Cabrera, N., Cornu, C., Boussageon, R., Moulin, P., Lega, J.C., Gueyffier, F., Cucherat, M., and Grenet, G. (2022). SGLT2 inhibitors in type 2 diabetes: a systematic review and meta-analysis of cardiovascular outcome trials balancing their risks and benefits. Diabetologia *65*, 2000–2010. https://doi.org/10.1007/s00125-022-05773-8.

30. Wright, E.M. (2020). SGLT2 and cancer. Pflügers Archiv *472*, 1407–1414.

31. Villani, L.A., Smith, B.K., Marcinko, K., Ford, R.J., Broadfield, L.A., Green, A.E., Houde, V.P., Muti, P., Tsakiridis, T., and Steinberg, G.R. (2016). The diabetes medication Canagliflozin reduces cancer cell proliferation by inhibiting mitochondrial complex-I supported respiration. Mol. Metabol. *5*, 1048–1056.

32. Au Yeung, S.L., and Schooling, C.M. (2019). Impact of glycemic traits, type 2 diabetes and metformin use on breast and prostate cancer risk: a Mendelian randomization study. BMJ Open Diabetes Res. Care *7*, e000872.

33. Packer, M., Anker, S.D., Butler, J., Filippatos, G., Pocock, S.J., Carson, P., Januzzi, J., Verma, S., Tsutsui, H., Brueckmann, M., et al. (2020). Cardiovascular and Renal Outcomes with Empagliflozin in Heart Failure. N. Engl. J. Med. *383*, 1413–1424.

34. Rawla, P. (2019). Epidemiology of Prostate Cancer. World J. Oncol. *10*, 63–89.

35. Hemani, G., Zheng, J., Elsworth, B., Wade, K.H., Haberland, V., Baird, D., Laurin, C., Burgess, S., Bowden, J., Langdon, R., et al. (2018). The MR-Base platform supports systematic causal inference across the human phenome. Elife *7*, e34408. https://doi.org/10.7554/eLife.34408.

36. Giambartolomei, C., Vukcevic, D., Schadt, E.E., Franke, L., Hingorani, A.D., Wallace, C., and Plagnol, V. (2014). Bayesian test for colocalisation between pairs of genetic association studies using summary statistics. PLoS Genet. *10*, e1004383.

37. Kachuri, L., Hoffmann, T.J., Jiang, Y., Berndt, S.I., Shelley, J.P., Schaffer, K., Machiela, M.J., Freedman, N.D., Huang, W.Y., Li, S.A., and Easterlin, R. (2022). Leveraging genetic determinants of prostate-specific antigen levels towards improving prostate cancer screening. Preprint at medRxiv. https://doi.org/10.1101/2022.04.18.22273850.

38. Saisho, Y. (2020). SGLT2 Inhibitors: the Star in the Treatment of Type 2 Diabetes? Diseases *8*, 14. https://doi.org/10.3390/diseases8020014.

39. Consortium, G.T.E. (2020). The GTEx Consortium atlas of genetic regulatory effects across human tissues. Science *369*, 1318–1330.

40. Võsa, U., Claringbould, A., Westra, H.-J., Bonder, M.J., Deelen, P., Zeng, B., Kirsten, H., Saha, A., Kreuzhuber, R., Yazar, S., et al. (2021). Large-scale cis- and trans-eQTL analyses identify thousands of genetic loci and polygenic scores that regulate blood gene expression. Nat. Genet. *53*, 1300–1310.

41. Chen, J., Spracklen, C.N., Marenne, G., Varshney, A., Corbin, L.J., Luan, J., Willems, S.M., Wu, Y., Zhang, X., Horikoshi, M., et al. (2021). The trans-ancestral genomic architecture of glycemic traits. Nat. Genet. *53*, 840–860.

42. Sun, B.B., Maranville, J.C., Peters, J.E., Stacey, D., Staley, J.R., Blackshaw, J., Burgess, S., Jiang, T., Paige, E., Surendran, P., et al. (2018). Genomic atlas of the human plasma proteome. Nature *558*, 73–79.

43. Folkersen, L., Fauman, E., Sabater-Lleal, M., Strawbridge, R.J., Frånberg, M., Sennblad, B., Baldassarre, D., Veglia, F., Humphries, S.E., Rauramaa, R., et al. (2017). Mapping of 79 loci for 83 plasma protein biomarkers in cardiovascular disease. PLoS Genet. *13*, e1006706.

44. Suhre, K., Arnold, M., Bhagwat, A.M., Cotton, R.J., Engelke, R., Raffler, J., Sarwath, H., Thareja, G., Wahl, A., DeLisle, R.K., et al. (2017). Connecting genetic risk to disease end points through the human blood plasma proteome. Nat. Commun. *8*, 14357.

45. Yao, C., Chen, G., Song, C., Keefe, J., Mendelson, M., Huan, T., Sun, B.B., Laser, A., Maranville, J.C., Wu, H., et al. (2018). Genome-wide mapping of plasma protein QTLs identifies putatively causal genes and pathways for cardiovascular disease. Nat. Commun. *9*, 3268.

46. Emilsson, V., Ilkov, M., Lamb, J.R., Finkel, N., Gudmundsson, E.F., Pitts, R., Hoover, H., Gudmundsdottir, V., Horman, S.R., Aspelund, T., et al. (2018). Co-regulatory networks of human serum proteins link genetics to disease. Science *361*, 769–773. https://doi.org/10.1126/science.aaq1327.

47. Võsa, U., Claringbould, A., Westra, H.-J., Bonder, M.J., Deelen, P., Zeng, B., Kirsten, H., Saha, A., Kreuzhuber, R., Yazar, S., et al. (2021). Large-scale cis- and trans-eQTL analyses identify thousands of genetic loci and polygenic scores that regulate blood gene expression. Nat. Genet. *53*, 1300–1310. https://doi.org/10.1038/s41588-021-00913-z.

48. Aguet, F., Barbeira, A.N., Bonazzola, R., Brown, A., Castel, S.E., Jo, B., Kasela, S., Kim-Hellmuth, S., Liang, Y., Oliva, M., et al. (2019). The GTEx Consortium atlas of genetic regulatory effects across human tissues. Preprint at bioRxiv. https://doi.org/10.1101/787903.

49. Zheng, J., Haberland, V., Baird, D., Walker, V., Haycock, P.C., Hurle, M.R., Gutteridge, A., Erola, P., Liu, Y., Luo, S., et al. (2020). Phenome-wide Mendelian randomization mapping the influence of the plasma proteome on complex diseases. Nat. Genet. *52*, 1122–1131.

50. Mahajan, A., Taliun, D., Thurner, M., Robertson, N.R., Torres, J.M., Rayner, N.W., Payne, A.J., Steinthorsdottir, V., Scott, R.A., Grarup, N., et al. (2018). Fine-mapping type 2 diabetes loci to single-variant resolution using high-density imputation and islet-specific epigenome maps. Nat. Genet. *50*, 1505–1513.

51. Skrivankova, V.W., Richmond, R.C., Woolf, B.A.R., Yarmolinsky, J., Davies, N.M., Swanson, S.A., VanderWeele, T.J., Higgins, J.P.T., Timpson, N.J., Dimou, N., et al. (2021). Strengthening the Reporting of Observational Studies in Epidemiology Using Mendelian Randomization: The STROBE-MR Statement. JAMA *326*, 1614–1621.

52. Skrivankova, V.W., Richmond, R.C., Woolf, B.A.R., Davies, N.M., Swanson, S.A., VanderWeele, T.J., Timpson, N.J., Higgins, J.P.T., Dimou, N., Langenberg, C., et al. (2021). Strengthening the reporting of observational studies in epidemiology using mendelian randomisation (STROBE-MR): explanation and elaboration. BMJ *375*, n2233.

53. Burgess, S., Zuber, V., Valdes-Marquez, E., Sun, B.B., and Hopewell, J.C. (2017). Mendelian randomization with fine-mapped genetic data: Choosing from large numbers of correlated instrumental variables. Genet. Epidemiol. *41*, 714–725.

54. Sanderson, E., Davey Smith, G., Windmeijer, F., and Bowden, J. (2019). An examination of multivariable Mendelian randomization in the single-sample and two-sample summary data settings. Int. J. Epidemiol. *48*, 713–727. https://doi.org/10.1093/ije/dyy262.

55. Davey Smith, G., Holmes, M.V., Davies, N.M., and Ebrahim, S. (2020). Mendel's laws, Mendelian randomization and causal inference in observational data: substantive and nomenclatural issues. Eur. J. Epidemiol. *35*, 99–111.

56. Burgess, S., and Thompson, S.G.; CRP CHD Genetics Collaboration (2011). CRP CHD Genetics Collaboration. Avoiding bias from weak instruments in Mendelian randomization studies. Int. J. Epidemiol. *40*, 755–764.

57. Burgess, S., and Thompson, S.G. (2015). Multivariable Mendelian randomization: the use of pleiotropic genetic variants to estimate causal effects. Am. J. Epidemiol. *181*, 251–260.

58. Kazmi, N., Haycock, P., Tsilidis, K., Lynch, B.M., Truong, T., PRACTICAL Consortium, CRUK, BPC3, CAPS, PEGASUS; Martin, R.M., and Lewis, S.J. (2020). Appraising causal relationships of dietary, nutritional and physical-activity exposures with overall and aggressive prostate

CellPress
OPEN ACCESS

cancer: two-sample Mendelian-randomization study based on 79 148 prostate-cancer cases and 61 106 controls. Int. J. Epidemiol. *49*, 587–596.

59. Bowden, J., Del Greco M, F., Minelli, C., Davey Smith, G., Sheehan, N., and Thompson, J. (2017). A framework for the investigation of pleiotropy in two-sample summary data Mendelian randomization. Stat. Med. *36*, 1783–1802.

60. Bowden, J., Del Greco, M.F., Minelli, C., Zhao, Q., Lawlor, D.A., Sheehan, N.A., Thompson, J., and Davey Smith, G. (2019). Improving the accuracy of two-sample summary data Mendelian randomization: moving beyond the NOME assumption. Int. J. Epidemiol. *48*, 728–742.

61. Bowden, J., Davey Smith, G., Haycock, P.C., and Burgess, S. (2016). Consistent Estimation in Mendelian Randomization with Some Invalid Instruments Using a Weighted Median Estimator. Genet. Epidemiol. *40*, 304–314.

62. Hartwig, F.P., Davey Smith, G., and Bowden, J. (2017). Robust inference in summary data Mendelian randomization via the zero modal pleiotropy assumption. Int. J. Epidemiol. *46*, 1985–1998. https://doi.org/10.1093/ije/dyx102.

63. Lu, J., He, J., Li, M., Tang, X., Hu, R., Shi, L., Su, Q., Peng, K., Xu, M., Xu, Y., et al. (2019). Predictive Value of Fasting Glucose, Postload Glucose, and Hemoglobin A1c on Risk of Diabetes and Complications in Chinese Adults. Diabetes Care *42*, 1539–1548.

64. Lu, J., Wang, S., Li, M., Gao, Z., Xu, Y., Zhao, X., Hu, C., Zhang, Y., Liu, R., Hu, R., et al. (2021). Association of Serum Bile Acids Profile and Pathway Dysregulation With the Risk of Developing Diabetes Among Normoglycemic Chinese Adults: Findings From the 4C Study. Diabetes Care *44*, 499–510.

65. Lu, J., Li, M., Xu, Y., Bi, Y., Qin, Y., Li, Q., Wang, T., Hu, R., Shi, L., Su, Q., et al. (2020). Early Life Famine Exposure, Ideal Cardiovascular Health Metrics, and Risk of Incident Diabetes: Findings From the 4C Study. Diabetes Care *43*, 1902–1909.

66. Wang, T.G., Lu, J.L., Shi, L.X., Chen, G., Xu, M., Xu, Y., Su, Q., Mu, Y.M., Chen, L.L., Hu, R.Y., et al. (2020). Association of insulin resistance and beta-cell dysfunction with incident diabetes among adults in China: a nationwide, population-based, prospective cohort study. Lancet Diabetes Endocrinol. *8*, 115–124.

67. Sudlow, C., Gallacher, J., Allen, N., Beral, V., Burton, P., Danesh, J., Downey, P., Elliott, P., Green, J., Landray, M., et al. (2015). UK biobank: an open access resource for identifying the causes of a wide range of complex diseases of middle and old age. PLoS Med. *12*, e1001779.

68. Lourida, I., Hannon, E., Littlejohns, T.J., Langa, K.M., Hyppönen, E., Kuzma, E., and Llewellyn, D.J. (2019). Association of Lifestyle and Genetic Risk With Incidence of Dementia. JAMA *322*, 430–437.

STAR★METHODS

KEY RESOURCES TABLE

REAGENT or RESOURCE	SOURCE	IDENTIFIER
Deposited data		
GWAS of HbA1c	UK Biobank	https://www.nealelab.is/uk-biobank
eQTL of *SLC5A2*	GTEX	N/A
GWAS of prostate cancer	PRACTICAL	http://practical.icr.ac.uk/
Cohort study with HbA1c and prostate cancer	The 4C study	https://www.rjh.com.cn/2018RJPortal/4c/index.shtml
Electronic healthcare data for usage of SGLT2i, DPP4i and prostate cancer events	The Shanghai Link Healthcare Database	https://pubmed.ncbi.nlm.nih.gov/37400692/
rowhead**Software and algorithms**		
MR models	Hemani et al.[35]	https://github.com/MRCIEU/TwoSampleMR
Colocalization analysis	Giambartolomei et al. [36]	https://github.com/chr1swallace/coloc

RESOURCE AVAILABLILITY

Lead contact

Further information and requests for resources and reagents should be directed to and will be fulfilled by the lead contact, Jie Zheng (jie.zheng@bristol.ac.uk).

Materials availability

This study did not involve any other unique materials.

Data and code availability

The data, analytic methods, and study materials will be made available to other researchers for purposes of reproducing the results. In more details, the genetic association data of the selected risk factors are available in the supplemental tables. The summary level GWAS statistics for the primary and secondary outcomes are available from the MRC IEU OpenGWAS database: https://gwas.mrcieu.ac.uk/. UK Biobank received ethical approval from the Research Ethics Committee (REC reference for UK Biobank is 11/NW/0382). The analytical script of the MR analysis that had been used in this study is available via the GitHub repository of the TwoSampleMR R package (17). Any additional information required to reanalyze the data reported in this work paper is available from the lead contact upon request.

EXPERIMENTAL MODEL AND SUBJECT DETAILS

The PRACTICAL and GAME-ON/ELLIPSE consortium

Genome-wide association study summary statistics were obtained from the PRACTICAL and GAME-ON/ELLIPSE consortia or Kachuri et al.[17,37] (n = 140,254 men from the general population). In total, eight prostate cancer related phenotypes were selected as outcomes for this study: total-, aggressive-, early-onset-, high aggressive vs. low aggressive-, high aggressive vs. low and intermediate aggressive-, advanced stage vs. localized stage prostate cancer. Advanced prostate cancer was defined as metastatic disease or Gleason score (GS) $\geq$ 8 or PSA >100 or prostate cancer death; early-onset refers to prostate cancer onset before age 55; low aggressive refers to T stage from the TNM staging $\leq$ T1, and GS $\leq$ 6, and PSA<10; intermediate aggressive refers to T stage: T2, and GS = 7, and PSA 10–20; and high aggressive refers to T stage: T3/T4 or N1 or M1 or GS $\geq$ 8 or PSA >20. PSA levels were included as they drive prostate cancer diagnoses, and we wanted to exclude an effect of the exposures on PSA that could bias the prostate cancer associations. Detailed information of the prostate cancer related outcomes was listed in Table S5.

The Shanghai Link Healthcare Database

The Shanghai Link Healthcare Database (SLHD) is developed and operated by the Shanghai Hospital Development Center (SHDC),[19] which is an administrative department of the Shanghai Municipal Government. The SHDC is responsible for the surveillance of 35 tertiary hospitals in Shanghai. In China, government-run hospitals are classified as primary (grade I), secondary (grade II), or tertiary (grade III) hospitals according to their abilities in medical care, medical education, and medical research, with tertiary hospitals being the best. According to administrative regulations, all 35 tertiary hospitals are required to upload general medical practice

data (i.e., outpatient visits, emergency department visits, and hospital admissions) to the SLHD. Any personally identifiable information is scrambled to protect privacy. The SLHD has released data for academic research since 2013, which requires review and approval to access.

The China Cardiometabolic and Cancer Cohort (4C) study

The China Cardiometabolic and Cancer Cohort (4C) study was a multi-center, population-based, prospective cohort study aiming to demonstrate whether abnormal glucose metabolism (diabetes and prediabetes) was associated with increased risk for cancer in the Chinese population and to identify factors that modify the risk of cancer among individuals with abnormal glucose metabolism.[6] Between 2011 and 2012, a total of 259,657 individuals aged 40 years and older were recruited from 25 communities of various regions of China. Eligible men and women aged $\geq$40 years were identified from local resident registration systems. Trained community health workers visited eligible individuals' homes and invited them to participate in the study.

METHOD DETAILS

Causal inference analyses using Mendelian randomization

Identification of drug target of SGLT2 and exposure data

This study investigated drug target for SGLT2 inhibitors. The drug targeted gene of for SGLT2, SLC5A2 was well defined in the literature.[38]

Three sets of genetic instruments were used to proxy effect of SGLT2 inhibition (Figure S1). For main drug target MR, summary data were obtained from a GWAS of HbA1c levels in the UK Biobank (n = 159,160 males), in which genetic variants associated with HbA1c in the SGLT2 region were selected as instruments. For the validation MR, a set of genetic variants associated with both HbA1c and expression levels of SGLT2 (data from the GTEX and eQTLGen consortia [$n \leq$ 31,684][39,40]).

For independent validation MR analyses, the GWAS of HbA1c levels from the MAGIC consortium[41] were used. The primary MAGIC GWAS was a *trans*-ancestry meta-analysis, for which we consider population structure may be a confounder to bias the MR estimates. We therefore used the European-only GWAS results from 146,806 European individuals. In addition, since the genetic effects of the MAGIC HbA1c GWAS was scaled to percentage unit in the original study. We conducted a beta transformation for the genetic effects of HbA1c. After transformation, the unit of HbA1c GWAS was changed to standard deviation (SD) decreasing unit. By applying this transformation, the MR effect estimates were comparable between UK Biobank and MAGIC. In addition, For the MAGIC GWAS, individuals with type 1 or type 2 diabetes, with usage of diabetes-relevant medications or has a fasting glucose 7 mmol L^{-1}, 2-h glucose $\geq$11.1 mmol L^{-1} or HbA1c $\geq$ 6.5% were excluded from the analysis.

Instrument selection

As demonstrated in Figure S1, we applied three instrument selection approaches to select genetic instruments for SGLT2 inhibition from two independent datasets.

The first approach selected SGLT2 instruments from a classic drug target instrument selection process (primary instruments). The genetic variants associated with HbA1c with a region-wide association threshold of $p < 1 \times 10^{-6}$ in the SLC5A2 gene region (target gene for SGLT2 inhibition) were selected as candidate instruments. After selection, seven variants that proxying SGLT2 inhibition were selected as set 2 instruments for SGLT2 inhibition (Table S2).

The second approach selected instruments for the main drug target MR analyses (stringent instruments). Genetic variants associated with expression levels of drug target genes in a regional-wide significance threshold ($p < 0.001$) and HbA1c in a region-wide significance level ($p < 1 \times 10^{-6}$) in a genomic region near the drug target gene (±1Mb window) were selected as candidate instruments. We systematically scanned genetic variants associated with the expression levels of SLC5A2 using data from seven recent GWAS studies of genes level in 49 human tissues and proteins in plasma.[42–48] This is because targets for SGLT2 inhibition may influence glycemic traits via biological mechanisms in different tissues. A set of genetic colocalization methods[36,49] were then used to select genetic variants with shared causal variants of expression level of the drug target gene and HbA1c in the gene coding region. This step mapped 44 genetic variants for SGLT2 (Table S3). We further applied linkage disequilibrium (LD) clumping to select those with the lowest p value that had an LD (which refers to pairwise squared correlation [r^2]) less than 0.15 as this indicates weak correlation among the selected genetic variants. European population specific LD among variants were estimated from the 1000 Genomes Project (phase 3) implemented in the two-sample MR package.[35,44] After filtering, two variants were selected as instruments for SGLT2 inhibition (Table S2A).

The third approach selected instruments of SGLT2 inhibition from an independent dataset from MAGIC consortium. The genetic variants passed regional-wide association threshold of $p < 1 \times 10^{-5}$ in the SLC5A2 region were selected as candidate instruments. LD clumping with a threshold of 0.01 was further applied to select complete independent genetic variants as genetic instruments. After selection, one genetic variant that proxying SGLT2 inhibition were selected as instrument (Table S4).

Outcome selection for human genetics analysis

Eight prostate cancer related phenotypes were selected as outcomes for the MR analysis: total-, aggressive-, early-onset-, high aggressive vs. low aggressive-, high aggressive vs. low and intermediate aggressive-, advanced stage vs. localised stage prostate cancer. Detailed information of the prostate cancer related outcomes was listed in Table S5.

Mendelian randomization analyses

Germline genetic variants used to proxy SGLT2 inhibition were matched to prostate cancer datasets by orienting effects of the exposure and the outcome to the same effect allele. If an instrument was missing in the outcome dataset, a genetic variant with high LD ($r^2 > 0.8$) to the instrument was selected as a proxy instrument where possible. An inverse-variance weighted approach was used to combine variant-level Wald ratio estimates into an overall effect estimate. All MR estimates (odds ratios [ORs]) were scaled to SD unit to reflect the equivalent of a one SD unit (0.62%) reduction in HbA_{1c}.

In the main MR analyses, the effects of genetically proxied SGLT2 inhibition (using seven primary instruments) were estimated on total prostate cancer, its subtypes and PSA levels in the general male population (PRACTICAL and GAME-ON/ELLIPSE).[17] The effect of SGLT2 inhibition on T2DM[50] was estimated as a positive control analysis. For the validation MR analyses, the effects of SGLT2 inhibition on the prostate cancer related outcomes were estimated using the stringent instruments and instruments from the independent dataset (MAGIC).

We report findings according to the STROBE-MR (Strengthening the Reporting of Mendelian Randomization Studies) guidelines[51,52] (the STROBE-MR check list as Data S1, related to STAR Methods). The three key MR assumptions were tested using the sensitivity methods, including generalized inverse variance weighted (gIVW),[53] genetic colocalization,[36,49] phenome-wide association studies (including classic risk factors associated with SGLT2 instruments) using data from the IEU OpenGWAS database,[18] heterogeneity tests across instruments using Cochran's Q, weighted median and mode-based estimate approaches and Multivariable MR.[54]

In more details, MR exploits both Mendel's Law of Heredity.[55] The Law of Independent Assortment refers to the fact that alleles of genes in different parts of the genome are inherited independently. Compliance with this Law was evaluated using a generalized inverse variance weighted (gIVW) model,[53] which takes into account the weak LD ($r^2 = 0.089$) between the SGLT2 instruments.

The MR assumption of relevance was tested by generating estimates of the proportion of variance in each drug target explained by the instrument (R2) and F statistics. An F statistic of at least 10 is indicative of evidence against weak instrument bias (a reduction in statistical power to reject the null hypothesis when an instrument explains only a small proportion of variance in an exposure).[56]

The MR assumption of exchangeability was tested by performing a genetic colocalization analysis between the drug target and prostate cancer.[36,49] This can be used to assess whether false-positive drug target-disease associations were created due to confounding by LD between nearby genetic variants (genetic confounding). A posterior probability of colocalization over 70% between a drug target and prostate cancer was used as evidence of colocalization.

The MR assumption involving the exclusion restriction was tested using a whole set of sensitivity methods. First, the presence of an association between an instrument for SGLT2 inhibition and an off-target phenotype could provide evidence of horizontal pleiotropy (which means a genetic variant influences a phenotype through biological pathways that are independent of the exposure under investigation), which is a violation of the exclusion restriction criterion. A phenome-wide association study (PheWAS) of the genetic instruments for SGLT2 inhibition was performed among a comprehensive list of 22,479 human phenotypes included in the IEU OpenGWAS database.[18] If there was evidence of effect of genetic instruments for SGLT2 inhibition with unintended phenotypes at a genetic association threshold of 5×10^{-8}, multivariable analyses were performed to examine associations between the genetic instruments for SGLT2 inhibition and prostate cancer outcomes, adjusted for genetically proxied phenotype.[57]

Second, if there was evidence of genetic effect of the SGLT2 instruments on expression levels of other genes, where the expression levels of these genes were associated with prostate cancer, then this will violate the exclusion restriction assumption of MR. We therefore conducted a transcriptome wide variant lookup to identify all genes that are associated with the SGLT2 instruments with $p < 1 \times 10^{-4}$ (Table S11). Differential expression analysis was then applied for expression levels of these genes in prostate tumor tissue versus normal prostate tissue. If expression level did not different between the two tissues, we will be more confident that these genes are not likely to be pleiotropic exposures that linking SGLT2 instruments with prostate cancer risk.

Third, the violations of the exclusion restriction assumption were further tested by examining associations of the genetic instruments with four previously reported causal prostate cancer risk factors (accelerometer-based physical activity measurement, serum iron, body mass index and monounsaturated fatty acids).[58] A marginal MR threshold ($p < 0.05$) was used as evidence of a potential pleitropy effect of the genetic instruments for SGLT2 inhibition on prostate cancer via a prostate cancer risk factor.

Fourth, for genetic instruments for SGLT2 inhibition with two or more SNPs, evidence of horizontal pleiotropy was examined via the following sensitivity analyses: heterogeneity test across instruments using Cochran's Q and Rücker's Q,[59,60] weighted median[61] and mode-based estimate approaches.[62] Weighted median MR and mode estimator approaches[61,62] are two additional sensitivity analyses, which provide consistent causal estimates of the exposure on the outcome even when up to 50% (or up to 100% for the mode estimator approach) of the information contributing to the analysis comes from genetic variants that exhibit pleiotropy (or even the majority of information in the case of the mode-based MR).

If all MR sensitivity methods provide similar causal estimates of genetic proxied SGLT2 inhibition on prostate cancer, we are more confident that the causal estimates were robust to various MR assumptions.

Moreover, the SGLT2 instruments were associated with other 17 genes. We estimated whether the 17 genes were associated with glycemic traits or to have an interaction with any anti-diabetic or anti-cancer drugs. For all MR analyses, Bonferroni corrections were applied to establish multiple testing-adjusted thresholds. All the MR analyses were conducted using the TwoSampleMR R package v0.5.6.[35]

Observational analysis using electronic healthcare data

The survival analysis was conducted using data from the Shanghai Link Healthcare Database (SLHD),[19] a representative clinical database covering >99% of Shanghai residents.

Figure 3A illustrates the selection process of the study population. First, all males aged between 40 and 99 years newly treated with SGLT2 inhibitors or DPP4 inhibitors from March 1, 2017 to December 31, 2021 were identified. Cohort entry was defined as the date of the first prescription. Exclusion criteria were defined as follows: patients without any medical record before cohort entry; patients who had been treated with both SGLT2 inhibitors and DPP4 inhibitors; patients with a history of prostate cancer; patients with total prostate specific antigen (PSA) > 10 ng/mL prior to enrollment; patients with less than 1 day of follow-up. All patients were followed until diagnosis of prostate cancer or death, or December 31, 2021, whichever occurred first.

The following covariates that may affect prostate cancer risk and/or total PSA levels were adjusted in the cox model:

(1) demographic data (age),
(2) comorbidities of diabetes (benign prostatic hypertrophy, hypertension, dyslipidemia, diabetic complications, ischemic heart disease, peripheral vascular disease, heart failure, cerebrovascular diseases, chronic lung disease, moderate or severe kidney disease, moderate or severe liver disease, cancers),
(3) usage of other antidiabetic drugs (including metformin, insulin, glucagon-like peptide-1 receptor agonist, sulfonylurea, glinide, α-glucosidase inhibitor, and thiazolidinedione),
(4) and other medications (angiotensin converting enzyme inhibitor, angiotensin receptor blocker, calcium channel blocker, α/β-blockers, diuretic, statin, fibrate, aspirin, other antiplatelet drugs, non-steroidal anti-inflammatory drug, and 5α-reductase inhibitor).

These factors are built up based on existing electronic healthcare records of outpatient patients. All comorbidities and medications records were assessed by relevant medical records prior to cohort entry.

In addition to the original cohort, we also established a 1:1 propensity score matched cohort of SGLT2 inhibitors users and DPP4 inhibitors users (caliper: 0.20 standard deviation of the logit of the estimated propensity score). Standardized mean differences (SMDs) were calculated for all covariates between SGLT2 inhibitors users and DPP4 inhibitors users, with values less than 10% likely to indicate relative balance.

For the survival analysis, baseline characteristics of SGLT2 inhibitors users and DPP4 inhibitors users are presented as medians with interquartile ranges (IQRs) for continuous variables and frequencies with percentages for categorical variables. The crude incidence rate of prostate cancer-by-proxy was calculated by dividing the number of cases by the number of person-years. Cox proportional hazards models were used to estimate hazard ratios (HRs) and 95% confidence intervals (CIs) of incident prostate cancer-by-proxy, comparing SGLT2 inhibitors use with DPP4 inhibitors use. Sensitivity analyses were performed by setting different lag periods: 1-month, 2-month, 3-month, and 6-month lag period. Statistical analyses were performed using R language software (version 4.1.2).

In addition, the analysis of prostate cancer subtypes was not conducted using the electronic healthcare data in SLHD since key information such as T stage and Gleason score were not available in the electronic healthcare records.

Validation using prospective cohort data with over 10 years of follow-up

We estimated the association between HbA1c and incident prostate cancer during a median of 10.1 years of follow-up in the China Cardiometabolic and Cancer Cohort (4C) study.[6,63–66] After excluding participants with prostate cancer at baseline, we included 57,779 men aged 40 years or older in the final analysis. The study was approved by the Medical Ethics Committee of Ruijin Hospital, Shanghai Jiao-Tong University. All study participants provided written informed consent.

As described previously[6,63–66] HbA1c was determined by using high-performance liquid chromatography (VARIANT II System; Bio-Rad Laboratories) in the central laboratory located at Ruijin Hospital, Shanghai, China, which is certificated by the U.S. National Glycohemoglobin Standardization Program and passed the Laboratory Accreditation Program of the College of American Pathologists. Information on prostate cancer were collected from local death and disease registries of the National Disease Surveillance Point System and National Health Insurance System with use of the ICD 10 code "C61" in the study. Cox proportional hazards model was applied to estimate the hazard ratio of HbA1c on incident prostate cancer in the overall population (n = 57,779). A sensitivity analysis was performed in participants without receiving glucose-lowering therapy at baseline (n = 53,037). Age, body mass index, tobacco consumption, alcohol consumption, physical activity, and diet score were included as covariates in the model.

The prospective association of HbA1c with incident prostate cancer in UK Biobank

During revision, we were required to estimate the association of HbA1c with incident prostate cancer during the follow-up in UKB men. All people in the UK National Health Service registry aged between 40 and 69 years and living within a 25 mile radius from one of 22 study centers were invited to participate between 2006-2010.[67] In total 503,325 adults (5.5% of the ~9.2 million invited) were recruited into UK Biobank.[67] Ethical approval for UKB was obtained from the North West Multi-centre Research Ethics Committee, and our study was performed under UKB application number 15825.

Prostate cancer (defined using ICD 10 code C61) together with its diagnostic date were obtained from UKB linked hospital inpatient data (field ID 41270 and 41280). HbA1c at baseline (field ID 30750) was measured via HPLC analysis on a Bio-Rad VARIANT II Turbo by UKB, and outliers with levels outside four standard deviation unit from the mean were excluded. We followed the same analysis in 4C study to adjust for participants' age (field ID 21021), body mass index (field ID 21001), smoking status (field ID 20116), drinking status (field ID 20117), regular physical activity, and healthy diet score, all of which were measured at UKB baseline. Specially, regular physical activity was derived based on the number of at least 10-min moderate (field ID 884) and vigorous (filed ID 904) PA per week, and duration of moderate (field ID 894) and vigorous (field ID 914) PA per day.[68] Healthy diet score was derived based on UKB food frequency questionnaire, including fruits (field ID 1309, 1319), vegetables (field ID 1289, 1299), fish (field ID 1329, 1339), processed meats (field ID 1349), unprocessed red meats (field ID 1369, 1379, 1389), whole and refined grains (field ID 1438, 1448, 1458, 1468).[68]

Cox proportional hazards model was applied to estimate the hazard ratio of HbA1c on incident prostate cancer. We restricted our analysis in 161,422 male participants of European descent, who had no missingness in the exposure, outcome and all covariates. In sensitivity analysis, we further considered competing risk in the Cox model by adding an index of death (i.e., whether participants were dead due to other diseases) as a cluster.

QUALIFICATION AND STATISTICAL ANALYSIS

Data are presented as means ± standard error of the mean (SEM). All statistical analyses were conducted using R scripts. Multiple testing correction was conducted for each of the statistical analysis. The significance between two groups was assessed using unpaired Student's t tests. A Bonferroni corrected p value <0.05 was considered as a threshold for putative causal evidence.

Cell Reports Medicine, Volume *5*

Supplemental information

The effect of SGLT2 inhibition on prostate cancer: Mendelian randomization and observational analysis using electronic healthcare and cohort data

Jie Zheng, Jieli Lu, Jiying Qi, Qian Yang, Huiling Zhao, Haoyu Liu, Zhihe Chen, Lanhui Huang, Youqiong Ye, Min Xu, Yu Xu, Tiange Wang, Mian Li, Zhiyun Zhao, Ruizhi Zheng, Shuangyuan Wang, Hong Lin, Chunyan Hu, Celine Sze Ling Chui, Shiu Lun Au Yeung, Shan Luo, Olympia Dimopoulou, Padraig Dixon, Sean Harrison, Yi Liu, Jamie Robinson, James Yarmolinsky, Philip Haycock, Jinqiu Yuan, Sarah Lewis, Zhongshang Yuan, Tom R. Gaunt, George Davey Smith, Guang Ning, Richard M. Martin, Bin Cui, Weiqing Wang, and Yufang Bi

Supplementary documents

Supplementary Figure 1. Four sets of instruments been used in the Mendelian randomization analysis. Related to STAR Methods

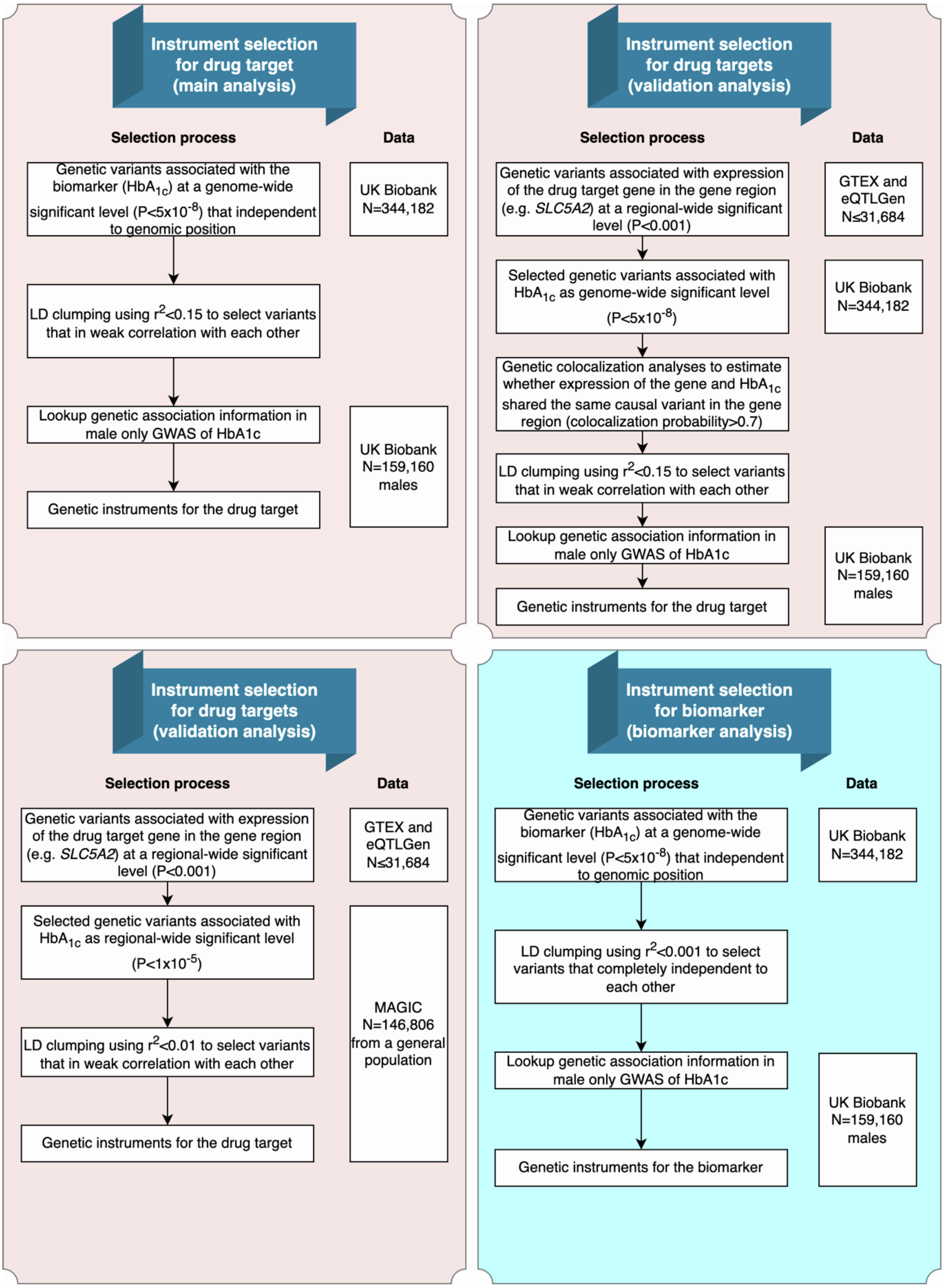

Supplementary Figure 2. Mendelian randomization assumptions. Related to STAR Methods

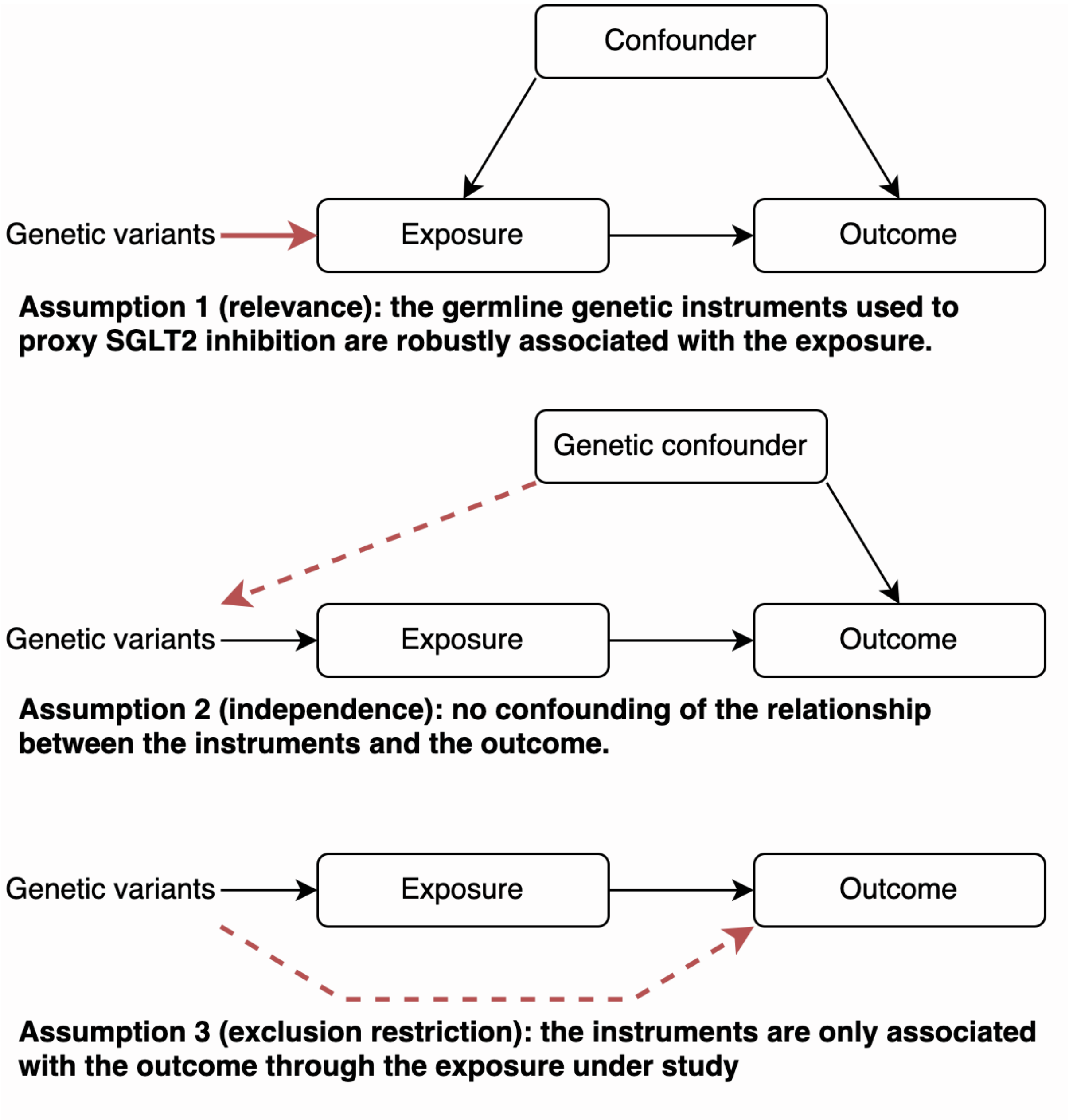

Supplementary Figure 3. Scatter plot and forest plot for the genetically proxied effect of SGLT2 inhibition on total prostate cancer. Related to STAR Methods

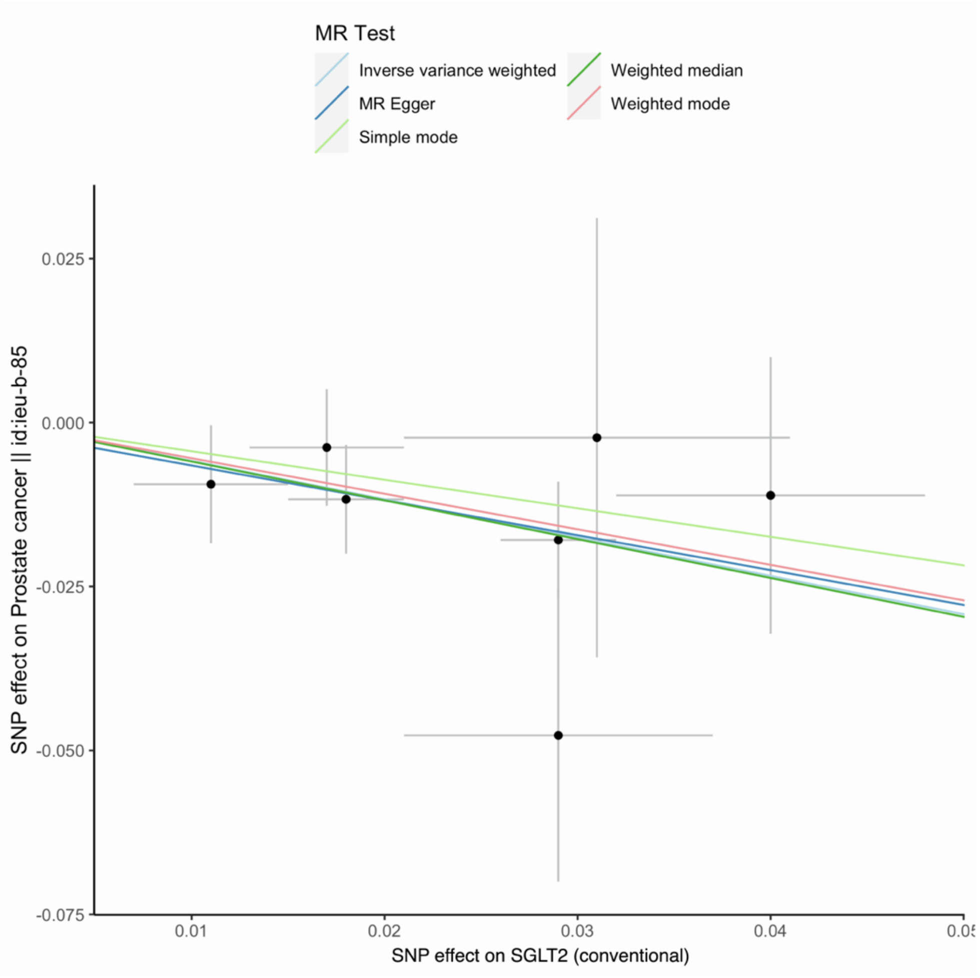

g

Supplementary Figure 4. Summary of key findings of the current study. Related to STAR Methods

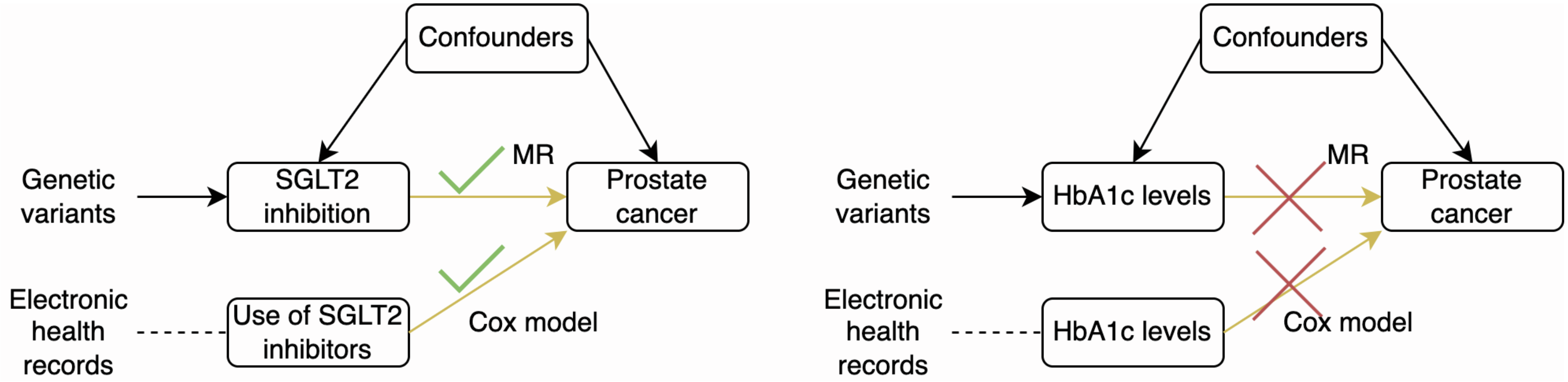

Supplementary Table 1. Baseline characteristics of users of sodium glucose cotransporter 2 (SGLT2) inhibitors and DPP4 inhibitors, before and after propensity score matching (for the survival analysis). Related to STAR Methods

	Original cohort			PS-matched cohort		
	SGLT2i (n = 26,988)	DPP4i (n = 54,134)	SMD	SGLT2i (n = 24,155)	DPP4i (n = 24,155)	SMD
Age, median (IQR)	62 (54-69)	63 (56-70)	14.2%	63 (55-69)	62 (55-69)	0.6%
Comorbidities, N (%)						
BPH	3,485 (12.91)	7,665 (14.16)	3.6%	3,138 (12.99)	3,256 (13.48)	1.4%
Hypertension	15,286 (56.64)	24,535 (45.32)	22.8%	13,456 (55.71)	13,581 (56.22)	1.0%
Dyslipidemia	6,824 (25.29)	10,386 (19.19)	14.7%	5,842 (24.19)	5,871 (24.31)	0.3%
Diabetic complications	5,920 (21.94)	9,703 (17.92)	10.1%	4,923 (20.38)	5,007 (20.73)	0.9%
Ischemic heart disease	9,364 (34.70)	12,104 (22.36)	27.6%	8,040 (33.29)	8,134 (33.67)	0.8%
Peripheral vascular disease	1,402 (5.19)	2,178 (4.02)	5.6%	1,242 (5.14)	1,251 (5.18)	0.2%
Heart failure	2,205 (8.17)	1,963 (3.63)	19.4%	1,661 (6.88)	1,662 (6.88)	<0.001
Cerebrovascular diseases	5,811 (21.53)	11,400 (21.06)	1.2%	5,247 (21.72)	5,316 (22.01)	0.7%
Chronic lung disease	3,750 (13.90)	7,335 (13.55)	1.0%	3,387 (14.02)	3,424 (14.18)	0.4%
Moderate or severe kidney disease	1,169 (4.33)	2,152 (3.98)	1.8%	1,061 (4.39)	1,100 (4.55)	0.8%
Moderate or severe liver disease	754 (2.79)	1,212 (2.24)	3.5%	650 (2.69)	658 (2.72)	0.2%
Other cancers	1,750 (6.48)	3,846 (7.10)	2.5%	1,607 (6.65)	1,625 (6.73)	0.3%
Antidiabetic drugs, N (%)						
Metformin	17,420 (64.55)	30,827 (56.95)	15.6%	15,199 (62.92)	15,320 (63.42)	1.0%
Insulin	12,191 (45.17)	22,753 (42.03)	6.3%	10,506 (43.49)	10,547 (43.66)	0.3%
GLP1RA	2,862 (10.60)	591 (1.09)	41.4%	625 (2.59)	591 (2.45)	0.9%
Sulfonylurea	8,573 (31.77)	17,515 (32.35)	1.3%	7,773 (32.18)	7,897 (32.69)	1.1%
Glinide	3,160 (11.71)	7,514 (13.88)	6.5%	2,859 (11.84)	2,908 (12.04)	0.6%
α-glucosidase inhibitor	9,958 (36.90)	21,510 (39.73)	5.8%	8,901 (36.85)	8,975 (37.16)	0.6%
Thiazolidinedione	5,594 (20.73)	8,222 (15.19)	14.5%	4,795 (19.85)	4,931 (20.41)	1.4%
Medications, N (%)						
ACEI	4,997 (18.52)	6,813 (12.59)	16.4%	4,283 (17.73)	4,294 (17.78)	0.1%
ARB	13,570 (50.28)	20,426 (37.73)	25.5%	11,736 (48.59)	11,790 (48.81)	0.4%
CCB	13,284 (49.22)	22,182 (40.98)	16.6%	11,665 (48.29)	11,797 (48.84)	1.1%
α/β-blockers	12,237 (45.34)	17,822 (32.92)	25.7%	10,594 (43.86)	10,693 (44.27)	0.8%
Diuretic	9,194 (34.07)	14,256 (26.33)	16.9%	7,971 (33.00)	7,984 (33.05)	0.1%
Statin	15,778 (58.46)	25,215 (46.58)	24.0%	13,705 (56.74)	13,852 (57.35)	1.2%
Fibrate	2,756 (10.21)	3,869 (7.15)	10.9%	2,284 (9.46)	2,283 (9.45)	<0.001
Aspirin	12,394 (45.92)	19,275 (35.61)	21.1%	10,777 (44.62)	10,917 (45.20)	1.2%
Other antiplatelet drugs	9,238 (34.23)	12,254 (22.64)	25.9%	7,985 (33.06)	8,015 (33.18)	0.3%
NSAID	10,399 (38.53)	19,863 (36.69)	3.8%	9,241 (38.26)	9,355 (38.73)	1.0%
5α-reductase inhibitor	1,362 (5.05)	3,458 (6.39)	5.8%	1,266 (5.24)	1,314 (5.44)	0.9%

Notes: PS-matched cohort, propensity score matched cohort; SGLT2i, sodium-glucose cotransporter-2 inhibitor; SMD, standardized mean difference.

Supplementary Table 3. Selection of genetic instruments that proxying SGLT2. Related to STAR Methods

Genetic association inforomation of the expression level of the genes													MR and colocalization of *SLC5A2* expression on HbA1c							
**Tissue**	**Gene.name**	**ENSG.ID**	**Phenotype**	**Variant_ID**	**Effect_al**	**Other_a**	**Effect_all**	**Beta**	**Se**	**P**	**N**	**beta.mr**	**se.mr**	**pval.mr**	**pass.mr**	**HbA1c-signal-in-region**	**LD-r2**	**pass.LD.check**
Lung	*SLC5A2*	ENSG00000140675.12	Lung_SLC5A2_rs9934336	rs9934336	A	G	0.252	0.522	0.045	2.85E-27	515	-0.021	0.005	1.89E-05	TRUE	rs11150626_C_T	99%	TRUE
Stomach	*SLC5A2*	ENSG00000140675.12	Stomach_SLC5A2_rs34497199	rs34497199	T	C	0.415	0.556	0.070	6.79E-14	324	-0.021	0.004	5.98E-07	TRUE	rs35856922_A_G	99%	TRUE
Artery_Tibial	*SLC5A2*	ENSG00000140675.12	Artery_Tibial_SLC5A2_rs11646054	rs11646054	C	G	0.414	-0.368	0.048	9.13E-14	584	-0.006	0.006	3.09E-01	FALSE	NA	NA	FALSE
Pancreas	*SLC5A2*	ENSG00000140675.12	Pancreas_SLC5A2_rs9934336	rs9934336	A	G	0.267	0.566	0.074	4.24E-13	305	-0.021	0.005	1.89E-05	TRUE	rs11150626_C_T	99%	TRUE
Colon_Sigmoid	*SLC5A2*	ENSG00000140675.12	Colon_Sigmoid_SLC5A2_rs11150624	rs11150624	T	C	0.442	-0.427	0.060	1.23E-11	318	-0.005	0.005	3.69E-01	FALSE	NA	NA	FALSE
Colon_Transverse	*SLC5A2*	ENSG00000140675.12	Colon_Transverse_SLC5A2_rs11865835	rs11865835	C	T	0.315	0.435	0.066	1.60E-10	368	-0.026	0.006	1.34E-05	TRUE	rs6565235_T_C	99%	TRUE
Whole_blood	*SLC5A2*	ENSG00000140675	Whole_blood_SLC5A2_rs6565236	rs6565236	T	A	0.249	0.084	0.014	9.86E-10	31191	-0.129	0.030	2.27E-05	TRUE	rs3934739_T_C	100%	TRUE
Brain_Cerebellum	*SLC5A2*	ENSG00000140675.12	Brain_Cerebellum_SLC5A2_rs8057029	rs8057029	C	A	0.699	-0.420	0.077	1.80E-07	209	0.019	0.006	1.29E-03	TRUE	rs28692853_A_C	48%	FALSE
Brain_Frontal_Cortex_BA9	*SLC5A2*	ENSG00000140675.12	Brain_Frontal_Cortex_BA9_SLC5A2_rs11150606	rs11150606	C	T	0.011	-2.259	0.491	9.51E-06	175	-0.001	0.004	8.77E-01	FALSE	NA	NA	FALSE
Artery_Aorta	*SLC5A2*	ENSG00000140675.12	Artery_Aorta_SLC5A2_rs140791727	rs140791727	T	C	0.008	1.661	0.371	1.04E-05	387	-0.012	0.008	1.05E-01	FALSE	NA	NA	FALSE
Esophagus_Gastroesophageal_Junction	*SLC5A2*	ENSG00000140675.12	Esophagus_Gastroesophageal_Junction_SLC5A2_rs11643752	rs11643752	A	G	0.414	-0.261	0.059	1.52E-05	330	0.003	0.009	7.12E-01	FALSE	NA	NA	FALSE
Brain_Cortex	*SLC5A2*	ENSG00000140675.12	Brain_Cortex_SLC5A2_rs12448775	rs12448775	T	C	0.044	0.996	0.228	2.26E-05	205	0.019	0.006	1.68E-03	TRUE	rs11644104_C_T	6%	FALSE
Brain_Cerebellar_Hemisphere	*SLC5A2*	ENSG00000140675.12	Brain_Cerebellar_Hemisphere_SLC5A2_rs8054784	rs8054784	C	T	0.583	-0.332	0.078	3.45E-05	175	0.005	0.007	4.92E-01	FALSE	NA	NA	FALSE
Prostate	*SLC5A2*	ENSG00000140675.12	Prostate_SLC5A2_rs4561482	rs4561482	G	A	0.643	0.350	0.083	4.03E-05	221	-0.011	0.007	9.38E-02	FALSE	NA	NA	FALSE
Brain_Hypothalamus	*SLC5A2*	ENSG00000140675.12	Brain_Hypothalamus_SLC5A2_rs138759654	rs138759654	A	C	0.012	1.477	0.352	5.08E-05	170	-0.009	0.007	1.96E-01	FALSE	NA	NA	FALSE
Skin_Not_Sun_Exposed_Suprapubic	*SLC5A2*	ENSG00000140675.12	Skin_Not_Sun_Exposed_Suprapubic_SLC5A2_rs74015509	rs74015509	C	T	0.017	0.955	0.240	7.86E-05	517	NA	NA	NA	FALSE	NA	NA	FALSE
Adipose_Subcutaneous	*SLC5A2*	ENSG00000140675.12	Adipose_Subcutaneous_SLC5A2_rs3841244	rs3841244	G	GT	0.528	0.211	0.053	8.93E-05	581	NA	NA	NA	FALSE	NA	NA	FALSE
Brain_Spinal_cord_cervical_c-1	*SLC5A2*	ENSG00000140675.12	Brain_Spinal_cord_cervical_c-1_SLC5A2_rs2073918	rs2073918	G	C	0.119	0.737	0.185	1.33E-04	126	-0.003	0.006	6.19E-01	FALSE	NA	NA	FALSE
Skin_Sun_Exposed_Lower_leg	*SLC5A2*	ENSG00000140675.12	Skin_Sun_Exposed_Lower_leg_SLC5A2_rs8058958	rs8058958	A	G	0.014	0.815	0.212	1.35E-04	605	NA	NA	NA	FALSE	NA	NA	FALSE
Uterus	*SLC5A2*	ENSG00000140675.12	Uterus_SLC5A2_rs35445454	rs35445454	T	C	0.275	0.430	0.109	1.00E-04	129	-0.030	0.006	1.24E-07	TRUE	rs113138456_G_A	81%	TRUE
Ovary	*SLC5A2*	ENSG00000140675.12	Ovary_SLC5A2_rs779234311	rs779234311	TGGGGC	T	0.018	1.347	0.353	2.08E-04	167	NA	NA	NA	FALSE	NA	NA	FALSE
Spleen	*SLC5A2*	ENSG00000140675.12	Spleen_SLC5A2_rs8044603	rs8044603	G	A	0.313	0.379	0.102	2.71E-04	227	0.009	0.007	2.24E-01	FALSE	NA	NA	FALSE
Adipose_Visceral_Omentum	*SLC5A2*	ENSG00000140675.12	Adipose_Visceral_Omentum_SLC5A2_rs4488457	rs4488457	G	T	0.682	0.239	0.065	2.72E-04	469	-0.055	0.011	2.90E-07	TRUE	rs7205195_A_G	100%	TRUE
Breast_Mammary_Tissue	*SLC5A2*	ENSG00000140675.12	Breast_Mammary_Tissue_SLC5A2_rs144959592	rs144959592	G	A	0.016	-0.780	0.219	4.19E-04	396	0.016	0.012	1.82E-01	FALSE	NA	NA	FALSE
Esophagus_Mucosa	*SLC5A2*	ENSG00000140675.12	Esophagus_Mucosa_SLC5A2_rs72796569	rs72796569	T	C	0.044	-0.436	0.126	6.12E-04	497	0.018	0.012	1.51E-01	FALSE	NA	NA	FALSE
Esophagus_Muscularis	*SLC5A2*	ENSG00000140675.12	Esophagus_Muscularis_SLC5A2_rs34235897	rs34235897	C	CT	0.123	-0.270	0.078	6.41E-04	465	NA	NA	NA	FALSE	NA	NA	FALSE
Thyroid	*SLC5A2*	ENSG00000140675.12	Thyroid_SLC5A2_rs78641771	rs78641771	T	C	0.019	-0.462	0.137	7.63E-04	574	-0.005	0.014	7.33E-01	FALSE	NA	NA	FALSE
Brain_Caudate_basal_ganglia	*SLC5A2*	ENSG00000140675.12	Brain_Caudate_basal_ganglia_SLC5A2_rs529028063	rs529028063	CT	C	0.013	1.571	0.461	8.40E-04	194	0.005	0.007	4.41E-01	FALSE	NA	NA	FALSE
Brain_Hippocampus	*SLC5A2*	ENSG00000140675.12	Brain_Hippocampus_SLC5A2_rs116872173	rs116872173	C	T	0.021	1.118	0.330	9.54E-04	165	-0.034	0.011	1.37E-03	TRUE	rs4889630_T_C	4%	FALSE
Brain_Nucleus_accumbens_basal_ganglia	*SLC5A2*	ENSG00000140675.12	Brain_Nucleus_accumbens_basal_ganglia_SLC5A2_rs117219760	rs117219760	G	T	0.050	0.711	0.212	1.00E-03	202	-0.012	0.012	2.87E-01	FALSE	NA	NA	FALSE
Brain_Substantia_nigra	*SLC5A2*	ENSG00000140675.12	Brain_Substantia_nigra_SLC5A2_rs77088799	rs77088799	T	A	0.022	-1.223	0.362	1.09E-03	114	-0.002	0.008	7.85E-01	FALSE	NA	NA	FALSE
Artery_Coronary	*SLC5A2*	ENSG00000140675.12	Artery_Coronary_SLC5A2_rs8057326	rs8057326	C	T	0.484	0.267	0.082	1.42E-03	213	-0.031	0.009	2.80E-04	TRUE	rs34742827_T_C	79%	TRUE
Whole_Blood	*SLC5A2*	ENSG00000140675.12	Whole_Blood_SLC5A2_rs41476751	rs41476751	C	T	0.216	-0.183	0.058	1.57E-03	670	0.008	0.018	6.56E-01	FALSE	NA	NA	FALSE
Nerve_Tibial	*SLC5A2*	ENSG00000140675.12	Nerve_Tibial_SLC5A2_rs9930811	rs9930811	G	A	0.388	0.188	0.060	1.80E-03	532	-0.087	0.013	8.68E-12	TRUE	rs7199585_C_T	100%	TRUE
Brain_Amygdala	*SLC5A2*	ENSG00000140675.12	Brain_Amygdala_SLC5A2_rs865900824	rs865900824	C	A	0.012	-1.743	0.549	1.97E-03	129	NA	NA	NA	FALSE	NA	NA	FALSE
Liver	*SLC5A2*	ENSG00000140675.12	Liver_SLC5A2_rs142133957	rs142133957	A	G	0.007	1.557	0.502	2.26E-03	208	0.010	0.006	7.10E-02	FALSE	NA	NA	FALSE
Minor_Salivary_Gland	*SLC5A2*	ENSG00000140675.12	Minor_Salivary_Gland_SLC5A2_rs11642535	rs11642535	A	C	0.097	0.530	0.174	2.86E-03	144	-0.018	0.007	5.35E-03	TRUE	rs9933843_C_T	16%	FALSE
Brain_Putamen_basal_ganglia	*SLC5A2*	ENSG00000140675.12	Brain_Putamen_basal_ganglia_SLC5A2_rs71374021	rs71374021	GCTC	G	0.559	-0.355	0.118	3.13E-03	170	-0.047	0.007	4.91E-13	TRUE	NA	NA	FALSE
Vagina	*SLC5A2*	ENSG00000140675.12	Vagina_SLC5A2_rs72785569	rs72785569	T	C	0.082	-0.531	0.176	3.24E-03	141	-0.017	0.006	6.46E-03	TRUE	rs11574632_T_C	46%	FALSE
Pituitary	*SLC5A2*	ENSG00000140675.12	Pituitary_SLC5A2_rs118009866	rs118009866	G	A	0.023	-0.659	0.223	3.47E-03	237	0.000	0.010	9.86E-01	FALSE	NA	NA	FALSE
Small_Intestine_Terminal_Ileum	*SLC5A2*	ENSG00000140675.12	Small_Intestine_Terminal_Ileum_SLC5A2_rs117391625	rs117391625	T	C	0.078	-0.372	0.128	4.22E-03	174	0.005	0.011	6.22E-01	FALSE	NA	NA	FALSE
Brain_Anterior_cingulate_cortex_BA24	*SLC5A2*	ENSG00000140675.12	Brain_Anterior_cingulate_cortex_BA24_SLC5A2_rs4613077	rs4613077	A	C	0.017	-1.200	0.430	6.13E-03	147	NA	NA	NA	FALSE	NA	NA	FALSE
Cells_Cultured_fibroblasts	*SLC5A2*	ENSG00000140675.12	Cells_Cultured_fibroblasts_SLC5A2_rs76378339	rs76378339	T	C	0.009	0.630	0.232	6.94E-03	483	-0.034	0.014	1.35E-02	TRUE	rs11644104_C_T	7%	FALSE
Testis	*SLC5A2*	ENSG00000140675.12	Testis_SLC5A2_rs35530358	rs35530358	C	CGT	0.717	0.086	0.032	7.83E-03	322	NA	NA	NA	FALSE	NA	NA	FALSE

Notes 1: After instrument selection, eight variants for nine gene-tissue combination passed the selection process. After LD clumping of these eight variants with r2<0.1, two variants were kept as primary instruments (listed in eTable S2)

Notes 2: Tissus, Gene.name, ENSG.ID and Phenotype are the expression levels of a gene in a specific tissue that had been proxied by the genetic instruments. Variant ID, CHR and Position are the ID of the genetic variant. Effect allele, other allele, effect allele freq, beta, SE, N and P are the genetic association information of the genetic variant on the exposure. Beta.mr, se.mr, pval.mr are the effect, standard error and p value of the MR analysis of expression of SCL5A2 on HbA1c. HbA1c-signal-in-region, LD-r2 and pass.LD.check are the approximate colocalization that been conducted between expression of SLC5A2 gene and HbA1c.

Supplementary Table 4. Genetic instruments been selected to proxy SGLT2 inhibition and HbA1c levels (data from MAGIC consortium). Related to STAR Methods

Phenotype	SNP	Effect_allele	Other_allele	ffect_allele_fre	Beta	Se	P	N	maf	r2 - variance e	Sum_r2	N_SNPs	F-statistics
SGLT2_MAGIC_new	rs8050500	C	T	0.467	-0.043	0.009	1.56E-06	128609	0.467	9.06E-04	9.06E-04	2	58.295

Notes: Phenotype is the exposure that been proxied by the genetic instruments. id.phenotype is the IEU OpenGWAS database ID of the outcome. Variant ID, CHR and Position are the ID, chromosome and position of the genetic variant. Effect allele, other allele, effect allele freq, beta, SE, N and P are the genetic association information of the genetic variant on the exposure.

Supplementary Table 5. Characteristics of outcome data been used in this study. Related to STAR Methods

Outcome data information								
**MR-phenotype name**	**Purpose**	**IEU-OpenGWASdb-ID**	**Case group**	**Control group**	**GWAS Model**	**n_cases**	**n_controls**	**n_total**
Total prostate cancer	PrCa risk	ieu-b-85	All PrCa cases	Non-PrCa controls	Logistic	79148	61106	140254
Advanced prostate cancer	PrCa risk	ieu-a-1238	Advanced PrCa	Non-PrCa controls	Logistic	15167	58308	73475
Early-onset prostate cancer	PrCa risk	ieu-a-1240	PrCa Age at Dx<=55	Non-PrCa controls	Logistic	6988	44256	51244
High vs low aggressive prostate cancer	PrCa risk	ieu-a-1243	High aggressive	Low aggressive	Logistic	15561	9739	25300
High vs low and intermediate aggressive prostate cancer	PrCa risk	ieu-a-1244	High aggressive	Low/intermediate aggressive	Logistic	20658	38093	58751
Advaced vs non-advanced prostate cancer	PrCa risk	ieu-a-1241	Advanced PrCa	Non Advanced PrCa	Logistic	14160	62421	76581
Gleason score	PrCa risk	ieu-a-1242	Continuous score	/	Linear	/	/	61978
PSA levels	PrCa diagnosis	/	Continuous level	/	Linear	/	/	95768
Type 2 diabetes	Validation	Mahajan 2018	Type 2 diabetes	/	Logistic	14160	62421	76581

*	low aggressive:	T stage from the TNM staging<=T1, and Gleason score (GS)<=6, and PSA<10;
**	intermediate aggressive	T stage: T2, and GS=7, and PSA 10~20;
***	high aggressive	T stage: T3/T4 or N1 or M1 or GS>=8 or PSA>20;
#	advanced	Metastatic disease or GS>=8 or PSA>100 or PrCa Death.

Supplementary Table 6. Mendelian randomization estimates of SGLT2 on prostate cancer using seven instruments selected from UK Biobank. Related to STAR Methods

exposure	outcome	method	nsnp	b	se	lci	uci	pval	Q	Q_df	Q_pval	egger_interc	se	pval	OR	LCI	UCI
SGLT2 conventional male	Advanced prostate cancer	MR Egger	7	1.866	0.939	0.025	3.707	0.104	4.029	5	0.545	-0.028	0.020	0.226	0.155	0.025	0.975
SGLT2 conventional male	Advanced prostate cancer	Weighted median	7	0.660	0.420	-0.163	1.483	0.116	NA	NA	NA	NA	NA	NA	0.517	0.227	1.177
SGLT2 conventional male	Advanced prostate cancer	Inverse variance weighted	7	0.655	0.332	0.003	1.306	0.049	5.931	6	0.431	NA	NA	NA	0.519	0.271	0.997
SGLT2 conventional male	Advanced prostate cancer	Simple mode	7	0.533	0.664	-0.769	1.834	0.453	NA	NA	NA	NA	NA	NA	0.587	0.160	2.158
SGLT2 conventional male	Advanced prostate cancer	Weighted mode	7	0.625	0.465	-0.287	1.537	0.228	NA	NA	NA	NA	NA	NA	0.535	0.215	1.332
SGLT2 conventional male	Advanced prostate cancer	gIVW	7	0.739	0.384	-0.014	1.492	0.054	4.974	6	0.547	NA	NA	NA	0.477	0.225	1.014
SGLT2 conventional male	Early-onset prostate cancer	MR Egger	7	0.652	1.386	-2.065	3.369	0.658	4.197	5	0.521	0.015	0.030	0.641	0.521	0.034	7.883
SGLT2 conventional male	Early-onset prostate cancer	Weighted median	7	0.792	0.645	-0.473	2.057	0.220	NA	NA	NA	NA	NA	NA	0.453	0.128	1.604
SGLT2 conventional male	Early-onset prostate cancer	Inverse variance weighted	7	1.296	0.488	0.339	2.252	0.008	4.443	6	0.617	NA	NA	NA	0.274	0.105	0.712
SGLT2 conventional male	Early-onset prostate cancer	Simple mode	7	0.869	0.943	-0.978	2.717	0.392	NA	NA	NA	NA	NA	NA	0.419	0.066	2.660
SGLT2 conventional male	Early-onset prostate cancer	Weighted mode	7	0.799	0.676	-0.526	2.123	0.282	NA	NA	NA	NA	NA	NA	0.450	0.120	1.692
SGLT2 conventional male	Early-onset prostate cancer	gIVW	7	1.090	0.563	-0.013	2.193	0.053	5.271	6	0.509	NA	NA	NA	0.336	0.112	1.014
SGLT2 conventional male	Advaced vs non-advanced prostate cancer	MR Egger	7	2.502	0.947	0.645	4.359	0.046	4.280	5	0.510	-0.054	0.020	0.045	0.082	0.013	0.524
SGLT2 conventional male	Advaced vs non-advanced prostate cancer	Weighted median	7	0.703	0.445	-0.169	1.576	0.114	NA	NA	NA	NA	NA	NA	0.495	0.207	1.185
SGLT2 conventional male	Advaced vs non-advanced prostate cancer	Inverse variance weighted	7	0.148	0.461	-0.756	1.052	0.749	11.343	6	0.078	NA	NA	NA	0.863	0.349	2.130
SGLT2 conventional male	Advaced vs non-advanced prostate cancer	Simple mode	7	-0.588	0.732	-2.023	0.847	0.453	NA	NA	NA	NA	NA	NA	1.800	0.429	7.563
SGLT2 conventional male	Advaced vs non-advanced prostate cancer	Weighted mode	7	0.755	0.477	-0.179	1.690	0.164	NA	NA	NA	NA	NA	NA	0.470	0.185	1.196
SGLT2 conventional male	Advaced vs non-advanced prostate cancer	gIVW	7	0.296	0.474	-0.634	1.226	0.533	8.987	6	0.174	NA	NA	NA	0.744	0.294	1.885
SGLT2 conventional male	Gleason score	MR Egger	7	0.602	0.408	-0.198	1.402	0.200	4.984	5	0.418	-0.016	0.009	0.137	NA	NA	NA
SGLT2 conventional male	Gleason score	Weighted median	7	0.090	0.192	-0.286	0.465	0.640	NA	NA	NA	NA	NA	NA	NA	NA	NA
SGLT2 conventional male	Gleason score	Inverse variance weighted	7	-0.072	0.169	-0.404	0.260	0.671	8.106	6	0.230	NA	NA	NA	NA	NA	NA
SGLT2 conventional male	Gleason score	Simple mode	7	-0.038	0.286	-0.598	0.522	0.898	NA	NA	NA	NA	NA	NA	NA	NA	NA
SGLT2 conventional male	Gleason score	Weighted mode	7	0.124	0.188	-0.245	0.493	0.535	NA	NA	NA	NA	NA	NA	NA	NA	NA
SGLT2 conventional male	Gleason score	gIVW	7	-0.052	0.171	-0.387	0.283	0.763	6.146	6	0.407	NA	NA	NA	NA	NA	NA
SGLT2 conventional male	High vs low aggressive prostate cancer	MR Egger	7	0.755	1.660	-2.498	4.008	0.668	7.465	5	0.188	-0.021	0.036	0.592	0.470	0.018	12.158
SGLT2 conventional male	High vs low aggressive prostate cancer	Weighted median	7	0.140	0.640	-1.113	1.393	0.827	NA	NA	NA	NA	NA	NA	0.869	0.248	3.045
SGLT2 conventional male	High vs low aggressive prostate cancer	Inverse variance weighted	7	-0.132	0.556	-1.221	0.957	0.812	7.953	6	0.242	NA	NA	NA	1.141	0.384	3.392
SGLT2 conventional male	High vs low aggressive prostate cancer	Simple mode	7	0.170	1.075	-1.937	2.277	0.880	NA	NA	NA	NA	NA	NA	0.844	0.103	6.936
SGLT2 conventional male	High vs low aggressive prostate cancer	Weighted mode	7	0.339	0.721	-1.075	1.753	0.655	NA	NA	NA	NA	NA	NA	0.713	0.173	2.931
SGLT2 conventional male	High vs low aggressive prostate cancer	gIVW	7	-0.364	0.648	-1.634	0.906	0.574	8.093	6	0.231	NA	NA	NA	1.439	0.404	5.125
SGLT2 conventional male	High vs low and intermediate aggressive prostate cancer	MR Egger	7	1.644	0.883	-0.088	3.375	0.122	3.432	5	0.634	-0.029	0.019	0.183	0.193	0.034	1.092
SGLT2 conventional male	High vs low and intermediate aggressive prostate cancer	Weighted median	7	0.627	0.411	-0.178	1.432	0.127	NA	NA	NA	NA	NA	NA	0.534	0.239	1.195
SGLT2 conventional male	High vs low and intermediate aggressive prostate cancer	Inverse variance weighted	7	0.368	0.314	-0.247	0.983	0.240	5.818	6	0.444	NA	NA	NA	0.692	0.374	1.280
SGLT2 conventional male	High vs low and intermediate aggressive prostate cancer	Simple mode	7	0.490	0.705	-0.891	1.871	0.513	NA	NA	NA	NA	NA	NA	0.613	0.154	2.439
SGLT2 conventional male	High vs low and intermediate aggressive prostate cancer	Weighted mode	7	0.647	0.475	-0.283	1.577	0.222	NA	NA	NA	NA	NA	NA	0.524	0.207	1.328
SGLT2 conventional male	High vs low and intermediate aggressive prostate cancer	gIVW	7	0.303	0.363	-0.409	1.015	0.404	5.333	6	0.502	NA	NA	NA	0.739	0.362	1.505
SGLT2 conventional male	Total prostate cancer	MR Egger	7	0.532	0.545	-0.537	1.601	0.374	3.069	5	0.689	0.001	0.012	0.923	0.587	0.202	1.710
SGLT2 conventional male	Total prostate cancer	Weighted median	7	0.592	0.247	0.109	1.076	0.016	NA	NA	NA	NA	NA	NA	0.553	0.341	0.897
SGLT2 conventional male	Total prostate cancer	Inverse variance weighted	7	0.584	0.194	0.204	0.964	0.003	3.079	6	0.799	NA	NA	NA	0.557	0.381	0.815
SGLT2 conventional male	Total prostate cancer	Simple mode	7	0.435	0.324	-0.199	1.070	0.227	NA	NA	NA	NA	NA	NA	0.647	0.343	1.221
SGLT2 conventional male	Total prostate cancer	Weighted mode	7	0.542	0.256	0.040	1.044	0.079	NA	NA	NA	NA	NA	NA	0.581	0.352	0.961
SGLT2 conventional male	Total prostate cancer	gIVW	7	0.571	0.225	0.130	1.011	0.011	2.618	6	0.855	NA	NA	NA	0.565	0.364	0.878
SGLT2 conventional male	PSA levels	MR Egger	7	-0.540	0.235	-1.000	-0.080	0.070	1.912	5	0.861	0.010	0.005	0.124	NA	NA	NA
SGLT2 conventional male	PSA levels	Weighted median	7	-0.125	0.111	-0.342	0.093	0.263	NA	NA	NA	NA	NA	NA	NA	NA	NA
SGLT2 conventional male	PSA levels	Inverse variance weighted	7	-0.137	0.085	-0.304	0.030	0.107	5.313	6	0.504	NA	NA	NA	NA	NA	NA
SGLT2 conventional male	PSA levels	Simple mode	7	-0.009	0.167	-0.336	0.318	0.959	NA	NA	NA	NA	NA	NA	NA	NA	NA
SGLT2 conventional male	PSA levels	Weighted mode	7	-0.126	0.111	-0.343	0.092	0.300	NA	NA	NA	NA	NA	NA	NA	NA	NA
SGLT2 conventional male	PSA levels	gIVW	7	-0.132	0.102	-0.331	0.067	0.195	6.387	6	0.381	NA	NA	NA	NA	NA	NA
SGLT2 conventional male	Type 2 diabetes	Inverse variance weighted	6	0.422	0.150	0.128	0.715	0.005	3.087	5	0.687	NA	NA	NA	0.656	0.489	0.880
SGLT2 conventional male	Type 2 diabetes	MR Egger	6	-0.150	0.420	-0.974	0.674	0.739	0.967	4	0.915	0.013	0.009	0.219	1.162	0.510	2.649
SGLT2 conventional male	Type 2 diabetes	Weighted median	6	0.244	0.195	-0.138	0.627	0.211	NA	NA	NA	NA	NA	NA	0.783	0.534	1.148
SGLT2 conventional male	Type 2 diabetes	Simple mode	6	0.263	0.306	-0.336	0.862	0.429	NA	NA	NA	NA	NA	NA	0.769	0.422	1.399
SGLT2 conventional male	Type 2 diabetes	Weighted mode	6	0.256	0.202	-0.141	0.652	0.262	NA	NA	NA	NA	NA	NA	0.774	0.521	1.151
SGLT2 conventional male	Type 2 diabetes	gIVW	6	0.271	0.245	-0.208	0.751	0.268	7.673	4	0.175	NA	NA	NA	0.762	0.472	1.232

Notes: the conventional instrument selection process refers to seven genetic variants robustly associated with HbA1c (P<5e-8) in the *SLC5A2* region. nsnp means the number of instruments been used in the MR analysis. Method is the MR method been used in the analysis. Beta, se, pval, ci.lb

Supplementary Table 7. Mendelian randomization estimates of SGLT2 on prostate cancer based on the instruments selected either using a stringent selection process or from MAGIC data. Related to STAR Methods

exposure	outcome	method	nsnp	b	se	lci	uci	pval	Q	Q_df	Q_pval	egger_interc	se	pval	OR	LCI	UCI
SGLT2 ukbb male	Advanced prostate cancer	IVW	2	1.375	0.702	0.000	2.750	0.050	0.047	1	0.829	NA	NA	NA	0.253	0.064	1.000
SGLT2 ukbb male	Advanced prostate cancer	gIVW	2	1.401	0.785	-0.138	2.940	0.074	0.064	1	0.800	NA	NA	NA	0.246	0.053	1.147
SGLT2 ukbb male	Early-onset prostate cancer	IVW	2	0.591	1.757	-2.853	4.034	0.737	2.947	1	0.086	NA	NA	NA	0.554	0.018	17.334
SGLT2 ukbb male	Early-onset prostate cancer	gIVW	2	0.287	1.146	-1.958	2.532	0.802	4.063	1	0.044	NA	NA	NA	0.750	0.079	7.087
SGLT2 ukbb male	Advaced vs non-advanced prostate cancer	IVW	2	-0.085	0.709	-1.475	1.306	0.905	0.182	1	0.669	NA	NA	NA	1.088	0.271	4.371
SGLT2 ukbb male	Advaced vs non-advanced prostate cancer	gIVW	2	-0.033	0.794	-1.590	1.524	0.967	0.252	1	0.616	NA	NA	NA	1.033	0.218	4.902
SGLT2 ukbb male	Gleason score	IVW	2	0.132	0.310	-0.476	0.740	0.670	0.853	1	0.356	NA	NA	NA	0.876	0.477	1.609
SGLT2 ukbb male	Gleason score	gIVW	2	0.179	0.348	-0.503	0.861	0.607	1.182	1	0.277	NA	NA	NA	0.836	0.423	1.654
SGLT2 ukbb male	High vs low aggressive prostate cancer	IVW	2	0.830	1.061	-1.250	2.911	0.434	1.070	1	0.301	NA	NA	NA	0.436	0.054	3.489
SGLT2 ukbb male	High vs low aggressive prostate cancer	gIVW	2	0.654	1.151	-1.601	2.910	0.570	1.480	1	0.224	NA	NA	NA	0.520	0.055	4.958
SGLT2 ukbb male	High vs low and intermediate aggressive prostate cancer	IVW	2	-0.037	0.665	-1.341	1.266	0.955	0.031	1	0.861	NA	NA	NA	1.038	0.282	3.821
SGLT2 ukbb male	High vs low and intermediate aggressive prostate cancer	gIVW	2	-0.018	0.745	-1.479	1.443	0.981	0.042	1	0.837	NA	NA	NA	1.018	0.236	4.387
SGLT2 ukbb male	Total prostate cancer	IVW	2	1.240	0.410	0.436	2.044	0.002	0.077	1	0.782	NA	NA	NA	0.289	0.129	0.646
SGLT2 ukbb male	Total prostate cancer	gIVW	2	1.221	0.459	0.320	2.122	0.008	0.106	1	0.745	NA	NA	NA	0.295	0.120	0.726
SGLT2 ukbb male	PSA levels	IVW	2	-0.150	0.195	-0.532	0.232	0.442	0.411	1	0.521	NA	NA	NA	1.162	0.793	1.702
SGLT2 ukbb male	PSA levels	gIVW	2	-0.164	0.221	-0.597	0.268	0.456	0.580	1	0.446	NA	NA	NA	1.179	0.765	1.816
SGLT2 MAGIC	Advanced prostate cancer	Wald ratio	1	0.898	0.351	0.209	1.586	0.011	NA	NA	NA	NA	NA	NA	0.408	0.205	0.811
SGLT2_MAGIC	Early-onset prostate cancer	Wald ratio	1	0.372	0.516	-0.640	1.384	0.471	NA	NA	NA	NA	NA	NA	0.689	0.251	1.896
SGLT2 MAGIC	Advaced vs non-advanced prostate cancer	Wald ratio	1	0.712	0.356	0.014	1.409	0.046	NA	NA	NA	NA	NA	NA	0.491	0.244	0.986
SGLT2 MAGIC	Gleason score	Wald ratio	1	0.130	0.156	-0.175	0.436	0.403	NA	NA	NA	NA	NA	NA	0.878	0.647	1.191
SGLT2_MAGIC	High vs low aggressive prostate cancer	Wald ratio	1	0.065	0.512	-0.938	1.068	0.899	NA	NA	NA	NA	NA	NA	0.937	0.344	2.554
SGLT2 MAGIC	High vs low and intermediate aggressive prostate cancer	Wald ratio	1	0.433	0.335	-0.224	1.089	0.196	NA	NA	NA	NA	NA	NA	0.649	0.337	1.251
SGLT2_MAGIC	Total prostate cancer	Wald ratio	1	0.416	0.207	0.011	0.822	0.044	NA	NA	NA	NA	NA	NA	0.659	0.440	0.989
SGLT2 MAGIC	PSA levels	Wald ratio	1	-0.114	0.086	-0.283	0.055	0.185	NA	NA	NA	NA	NA	NA	1.121	0.947	1.327

Notes: the conventional instrument selection process refers to seven genetic variants robustly associated with HbA1c (P<5e-8) in the *SLC5A2* region. nsnp means the number of instruments been used in the MR analysis. Method is the MR method been used in the analysis. Beta, se, pval, ci.lb and ci.ub are the MR effect estimate, standard error, P value, lower and upper confidence intervals of the exposure on the outcome. Q, Q_df and Q_pval are the statistics that measuring heterogeneity across studies. Egger_intercept, egger_se and egger_pval are statistics to estimating levels of pleiotropy.OR, LCI and UCI are the odds ratio, lower and uppper confidence intervals of MR effect scaled from log odds ratio to odds ratio.

Supplementary Table 8. Genetic colocalization estimates of SGLT2 (proxied by HbA1c) on prostate cancer in the SCL5A2 region. Related to STAR Methods

Trait1	Trait2	nsnp	PP.H4/(PP.H3+PP.H4)
SGLT2 (proxied by its HbA1c lowering effect)	Total prostate cancer	2291	71.91%
SGLT2 (proxied by expression levels of *SLC5A2*)	Total prostate cancer	256	90.75%

Notes: we are under the assumption that PP.H0, PP.H1 and PP.H2 were unlikely to be true given strong MR evidence to support a genetic signal in both exposure (trait1) and outcome (trait2). We therefore estimated the probability of PP.H4/(PP.H3+PP.H4) as the evidence source for colocalization. nsnp means the number of genetic variants been used in the colocalization analysis.

Supplementary Table 9. Phenome-wide association (PheWAS) results of SLGT2 instruments. The PheWAS association with P value < 1e-5 was listed in this table. Related to STAR Methods

Phenotype	id.phenotype	Variant ID	chr	position	Effect_allele	Other_allele	Effect_allele_f	beta	se	p	n
Red cell distribution width	ebi-a-GCST90002404	rs8050500	16	31404571	C	T	0.446	-0.026	0.002	4.00E-35	408112
High light scatter reticulocyte percentage of red cells	ebi-a-GCST90002386	rs8050500	16	31404571	C	T	0.446	0.019	0.002	1.80E-19	408112
Sum basophil neutrophil counts	ebi-a-GCST004621	rs8050500	16	31404571	C	T	0.446	-0.032	0.004	5.22E-19	171529
Hip circumference	ukb-b-15590	rs55766044	16	31117698	T	C	0.280	0.018	0.002	1.70E-15	462117
Weight	ukb-b-11842	rs55766044	16	31117698	T	C	0.280	0.015	0.002	1.30E-14	461632
Red cell distribution width	ebi-a-GCST006804	rs8050500	16	31404571	C	T	0.446	-0.030	0.004	1.40E-14	116666
Waist circumference	ukb-b-9405	rs55766044	16	31117698	T	C	0.280	0.015	0.002	5.70E-14	462166
Trunk fat mass	ukb-b-20044	rs55766044	16	31117698	T	C	0.280	0.017	0.002	7.00E-14	454588
Weight	ukb-b-12039	rs55766044	16	31117698	T	C	0.280	0.014	0.002	1.00E-13	454893
Arm fat mass (right)	ukb-b-6704	rs55766044	16	31117698	T	C	0.280	0.016	0.002	1.30E-13	454757
Arm fat mass (left)	ukb-b-8338	rs55766044	16	31117698	T	C	0.280	0.016	0.002	2.20E-13	454684
Whole body fat mass	ukb-b-19393	rs55766044	16	31117698	T	C	0.280	0.016	0.002	6.50E-13	454137
Trunk fat percentage	ukb-b-16407	rs55766044	16	31117698	T	C	0.280	0.014	0.002	3.30E-12	454613
Leg fat mass (right)	ukb-b-18096	rs55766044	16	31117698	T	C	0.280	0.012	0.002	6.50E-12	454846
Arm fat percentage (right)	ukb-b-12854	rs55766044	16	31117698	T	C	0.280	0.012	0.002	7.80E-12	454789
Body fat percentage	ukb-b-8909	rs55766044	16	31117698	T	C	0.280	0.012	0.002	7.80E-12	454633
Low density lipoprotein cholesterol levels	ebi-a-GCST90002412	rs55766044	16	31117698	T	C	0.280	0.015	0.002	3.90E-13	431167
Leg fat mass (left)	ukb-b-7212	rs55766044	16	31117698	T	C	0.280	0.012	0.002	8.80E-12	454823
Arm fat percentage (left)	ukb-b-20188	rs55766044	16	31117698	T	C	0.280	0.012	0.002	9.10E-12	454724
diastolic blood pressure	ieu-b-39	rs55766044	16	31117698	T	C	0.279	0.130	0.020	5.04E-11	721678
Red cell distribution width	ebi-a-GCST90002404	rs28692853	16	31573030	A	C	0.506	-0.013	0.002	4.60E-10	408112
Basal metabolic rate	ukb-b-16446	rs55766044	16	31117698	T	C	0.280	0.009	0.001	2.60E-09	454874
Leg fat percentage (left)	ukb-b-18377	rs55766044	16	31117698	T	C	0.280	0.008	0.001	3.30E-09	454826
Leg fat percentage (right)	ukb-b-20531	rs55766044	16	31117698	T	C	0.280	0.008	0.001	4.20E-09	454854
Body mass index (BMI)	ukb-b-19953	rs55766044	16	31117698	T	C	0.280	0.013	0.002	4.30E-09	461460
Alzheimer's disease or family history of Alzheimer's disease	ebi-a-GCST90012877	rs55766044	16	31117698	T	C	0.280	-0.062	0.011	7.32E-09	472868
Body mass index (BMI)	ukb-b-2303	rs55766044	16	31117698	T	C	0.280	0.013	0.002	7.40E-09	454884
Arm predicted mass (left)	ukb-b-9093	rs55766044	16	31117698	T	C	0.280	0.008	0.001	1.30E-08	454655
Snoring	ukb-b-17400	rs55766044	16	31117698	T	C	0.280	-0.006	0.001	1.60E-08	430438
Diastolic blood pressure, automated reading	ukb-b-7992	rs55766044	16	31117698	T	C	0.280	0.013	0.002	1.80E-08	436424
Red cell distribution width	ebi-a-GCST90002404	rs28675289	16	31463252	T	C	0.045	-0.029	0.005	2.10E-08	408112
Worry	ebi-a-GCST006478	rs55766044	16	31117698	T	C	NA	-0.015	0.003	3.30E-08	348219
Snoring	ebi-a-GCST009760	rs55766044	16	31117698	T	C	NA	0.006	0.001	4.30E-08	408317
Comparative body size at age 10	ukb-b-4650	rs55766044	16	31117698	T	C	0.280	0.009	0.002	4.40E-08	454718

Notes: Phenotype is the exposure that been proxied by the genetic instruments. id.phenotype is the IEU OpenGWAS database ID of the outcome. Variant ID, CHR and Position are the ID, chromosome and position of the genetic variant. Effect allele, other allele, effect allele freq, beta, SE, N and P are the genetic association information of the genetic variant on the exposure.

Supplementary Table 10. Genetic instruments used for the multivariable Mendelian randomization model and the multivariable Mendelian randomization results. Related to STAR Methods

Supplementary Table 10A. Genetic instruments for SGLT2 and red blood cell distribution been used in the multivariable Mendelian randomization model. Related to STAR Methods

Phenotype	SNP	Effect_allele	Other_allele	Effect_allele_freq	Beta	Se	P
SGLT2 primary	rs1232538	T	G	0.273	0.011	0.004	4.20E-03
SGLT2 primary	rs28675289	T	C	0.044	-0.040	0.008	2.66E-06
SGLT2 primary	rs28692853	A	C	0.507	-0.018	0.003	3.16E-07
SGLT2 primary	rs45625038	T	C	0.030	0.031	0.010	2.45E-03
SGLT2 primary	rs55766044	T	C	0.280	0.017	0.004	1.54E-05
SGLT2 primary	rs557720784	T	C	0.054	0.029	0.008	2.16E-04
SGLT2 primary	rs8050500	C	T	0.446	-0.029	0.003	2.03E-17
Red blood cell (erythrocyte) count	rs1232538	T	G	0.273	-0.003	0.002	2.03E-01
Red blood cell (erythrocyte) count	rs28675289	T	C	0.044	-0.003	0.005	5.97E-01
Red blood cell (erythrocyte) count	rs28692853	A	C	0.508	-0.002	0.002	2.68E-01
Red blood cell (erythrocyte) count	rs45625038	T	C	0.030	-0.009	0.006	1.29E-01
Red blood cell (erythrocyte) count	rs55766044	T	C	0.280	-0.015	0.002	1.11E-10
Red blood cell (erythrocyte) count	rs557720784	T	C	0.053	0.000	0.005	9.24E-01
Red blood cell (erythrocyte) count	rs8050500	C	T	0.446	-0.009	0.002	8.67E-06

Phenotype	SNP	Effect_allele	Other_allele	Effect_allele_freq	Beta	Se	P
SGLT2 primary	rs1232538	T	G	0.273	0.011	0.004	4.20E-03
SGLT2 primary	rs28675289	T	C	0.044	-0.040	0.008	2.66E-06
SGLT2 primary	rs28692853	A	C	0.507	-0.018	0.003	3.16E-07
SGLT2 primary	rs45625038	T	C	0.030	0.031	0.010	2.45E-03
SGLT2 primary	rs55766044	T	C	0.280	0.017	0.004	1.54E-05
SGLT2 primary	rs8050500	C	T	0.446	-0.029	0.003	2.03E-17
Body mass index	rs1232538	T	G	0.602	0.003	0.003	2.70E-01
Body mass index	rs28675289	T	C	0.678	-0.008	0.006	2.30E-01
Body mass index	rs28692853	A	C	0.491	0.002	0.003	4.40E-01
Body mass index	rs45625038	T	C	0.693	0.002	0.011	8.80E-01
Body mass index	rs55766044	T	C	0.588	0.014	0.003	1.20E-05
Body mass index	rs8050500	C	T	0.523	0.005	0.003	5.90E-02

Phenotype	SNP	Effect_allele	Other_allele	Effect_allele_freq	Beta	Se	P
SGLT2 primary	rs1232538	T	G	0.273	0.011	0.004	4.20E-03
SGLT2 primary	rs28675289	T	C	0.044	-0.040	0.008	2.66E-06
SGLT2 primary	rs28692853	A	C	0.507	-0.018	0.003	3.16E-07
SGLT2 primary	rs45625038	T	C	0.030	0.031	0.010	2.45E-03
SGLT2 primary	rs55766044	T	C	0.280	0.017	0.004	1.54E-05
SGLT2 primary	rs557720784	T	C	0.054	0.029	0.008	2.16E-04
SGLT2 primary	rs8050500	C	T	0.446	-0.029	0.003	2.03E-17
Low density lipoprotein cholesterol levels	rs1232538	T	G	0.274	0.004	0.002	9.70E-02
Low density lipoprotein cholesterol levels	rs28675289	T	C	0.045	-0.010	0.005	4.00E-02
Low density lipoprotein cholesterol levels	rs28692853	A	C	0.506	0.000	0.002	9.00E-01
Low density lipoprotein cholesterol levels	rs45625038	T	C	0.029	0.008	0.006	1.50E-01
Low density lipoprotein cholesterol levels	rs55766044	T	C	0.280	0.015	0.002	3.90E-13
Low density lipoprotein cholesterol levels	rs557720784	T	C	0.052	-0.008	0.004	8.40E-02
Low density lipoprotein cholesterol levels	rs8050500	C	T	0.445	0.001	0.002	6.80E-01

Phenotype	SNP	Effect_allele	Other_allele	Effect_allele_freq	Beta	Se	P
SGLT2 primary	rs1232538	T	G	0.273	0.011	0.004	4.20E-03
SGLT2 primary	rs28675289	T	C	0.044	-0.040	0.008	2.66E-06
SGLT2 primary	rs28692853	A	C	0.507	-0.018	0.003	3.16E-07
SGLT2 primary	rs45625038	T	C	0.030	0.031	0.010	2.45E-03
SGLT2 primary	rs55766044	T	C	0.280	0.017	0.004	1.54E-05
SGLT2 primary	rs8050500	C	T	0.446	-0.029	0.003	2.03E-17
diastolic blood pressure	rs1232538	T	G	0.280	-0.003	0.019	8.91E-01
diastolic blood pressure	rs28675289	T	C	0.047	-0.006	0.044	8.86E-01
diastolic blood pressure	rs28692853	A	C	0.499	0.012	0.017	4.86E-01
diastolic blood pressure	rs45625038	T	C	0.028	-0.018	0.058	7.64E-01
diastolic blood pressure	rs55766044	T	C	0.279	0.130	0.020	5.04E-11
diastolic blood pressure	rs8050500	C	T	0.445	-0.009	0.018	6.11E-01

Phenotype	SNP	Effect_allele	Other_allele	Effect_allele_freq	Beta	Se	P
SGLT2 primary	rs1232538	T	G	0.273	0.011	0.004	4.20E-03
SGLT2 primary	rs28675289	T	C	0.044	-0.040	0.008	2.66E-06
SGLT2 primary	rs28692853	A	C	0.507	-0.018	0.003	3.16E-07
SGLT2 primary	rs45625038	T	C	0.030	0.031	0.010	2.45E-03
SGLT2 primary	rs55766044	T	C	0.280	0.017	0.004	1.54E-05
SGLT2 primary	rs8050500	C	T	0.446	-0.029	0.003	2.03E-17
Type 2 diabetes	rs1232538	T	G	0.290	0.0051	0.0042	0.227
Type 2 diabetes	rs28675289	T	C	0.055	-0.0059	0.0084	0.482
Type 2 diabetes	rs28692853	A	C	0.489	-0.0089	0.0037	0.016
Type 2 diabetes	rs45625038	T	C	0.023	-0.0032	0.0131	0.807
Type 2 diabetes	rs55766044	T	C	0.291	0.0114	0.0041	0.005
Type 2 diabetes	rs8050500	C	T	0.442	-0.0074	0.0038	0.048

Notes: Phenotype is the exposure that been proxied by the genetic instruments. Variant ID, CHR and Position are the ID, chromosome and position of the genetic variant. Effect allele, other allele, effect allele freq, beta, SE, N and P are the genetic association information of the genetic variant on the exposure.

Supplementary Table 10B. Multivariable Mendelian randomization estimate of SGLT2 on prostate cancer adjusted for red blood cell distribution. Related to STAR Methods

exposure	outcome	nsnp	beta	se	pval	ci.lb	ci.ub	OR	LCI	UCI
SGLT2 primary	Prostate cancer	7	0.580	0.143	4.96E-05	0.300	0.861	0.560	0.423	0.741
Red blood cell (erythrocyte) count	Prostate cancer	7	0.299	0.367	0.416	-0.421	1.019	0.741	0.361	1.524

exposure	outcome	nsnp	beta	se	pval	ci.lb	ci.ub	OR	LCI	UCI
SGLT2 primary	Prostate cancer	6	0.538	0.079	8.64E-12	0.383	0.692	0.584	0.500	0.681
Body mass index	Prostate cancer	6	-0.387	0.223	0.082	-0.824	0.049	1.473	0.952	2.280

exposure	outcome	nsnp	beta	se	pval	ci.lb	ci.ub	OR	LCI	UCI
SGLT2 primary	Prostate cancer	7	0.690	0.129	9.32E-08	0.436	0.943	0.502	0.390	0.646
Low density lipoprotein cholesterol levels	Prostate cancer	7	-0.663	0.352	0.060	-1.354	0.028	1.941	0.973	3.872

exposure	outcome	nsnp	beta	se	pval	ci.lb	ci.ub	OR	LCI	UCI
SGLT2 primary	Prostate cancer	6	0.561	0.090	3.91E-10	0.386	0.737	0.570	0.478	0.680
diastolic blood pressure	Prostate cancer	6	-0.044	0.030	0.148	-0.104	0.016	1.045	0.985	1.110

exposure	outcome	nsnp	beta	se	pval	ci.lb	ci.ub	OR	LCI	UCI
SGLT2 primary	Prostate cancer	6	0.494	0.212	1.97E-02	0.079	0.909	0.610	0.403	0.924
Type 2 diabetes	Prostate cancer	6	0.054	0.536	0.920	-0.996	1.104	0.947	0.332	2.707

Notes: nsnp means the number of instruments been used in the MR analysis. Beta, se, pval, ci.lb and ci.ub are the MR effect estimate, standard error, P value, lower and upper confidence intervals of the exposure on the outcome. OR, LCI and UCI are the odds ratio, lower and uppper confidence intervals of MR effect scaled from log odds ratio to odds ratio.

Supplementary Table 11. Genetic associations of the two SGLT2 instruments on expression level of other genes in nearby gene regions. Genetic variants with genetic association P<1e-4 was listed in this table. Related to STAR Methods

Gene ID	Gene	Variant ID	CHR	Position	Effect_allele	Other_allele	Effect_allele_freq	Beta	SE	N	P	Known glycemic gene?	Drug	Drug function
ENSG00000131797	CLUHP3	rs35445454	16	31699326	T	C	0.336	-0.549	0.012	17997	3.27E-300	NO	NA	NA
ENSG00000156886	ITGAD	rs9930811	16	31400360	G	A	0.351	-0.290	0.012	31346	2.67E-124	NO	NA	NA
ENSG00000261731	CTD-2358C21.4	rs35445454	16	31699326	T	C	0.336	-0.292	0.012	3831	2.70E-123	NO	NA	NA
ENSG00000140678	ITGAX	rs9930811	16	31400360	G	A	0.351	-0.287	0.012	26057	1.19E-121	NO	NA	NA
ENSG00000131797	CLUHP3	rs9930811	16	31400360	G	A	0.351	-0.180	0.012	22545	3.91E-48	NO	NA	NA
ENSG00000140678	ITGAX	rs35445454	16	31699326	T	C	0.336	-0.181	0.013	21723	3.30E-47	NO	NA	NA
ENSG00000140691	ARMC5	rs9930811	16	31400360	G	A	0.351	0.165	0.012	30597	1.56E-40	NO	NA	NA
ENSG00000156886	ITGAD	rs35445454	16	31699326	T	C	0.336	-0.139	0.013	26798	1.77E-28	NO	NA	NA
ENSG00000103507	BCKDK	rs9930811	16	31400360	G	A	0.351	-0.119	0.012	31346	7.59E-22	NO	NA	NA
ENSG00000140682	TGFB1I1	rs9930811	16	31400360	G	A	0.351	0.119	0.012	31132	8.86E-22	NO	NA	NA
ENSG00000261245	RP11-120K18.3	rs9930811	16	31400360	G	A	0.351	-0.105	0.012	4656	4.16E-17	NO	NA	NA
ENSG00000260911	RP11-196G11.2	rs9930811	16	31400360	G	A	0.351	0.097	0.012	5164	7.38E-15	NO	NA	NA
ENSG00000103496	STX4	rs9930811	16	31400360	G	A	0.351	0.094	0.012	31132	3.41E-14	NO	PHENPROCOUMON	Anticoagulant
ENSG00000197302	ZNF720	rs35445454	16	31699326	T	C	0.336	-0.093	0.013	26758	1.07E-13	NO	NA	NA
ENSG00000167394	ZNF668	rs9930811	16	31400360	G	A	0.351	-0.077	0.012	31346	6.76E-10	NO	NA	NA
ENSG00000103549	RNF40	rs9930811	16	31400360	G	A	0.351	-0.075	0.012	31346	1.45E-09	NO	NA	NA
ENSG00000261731	CTD-2358C21.4	rs9930811	16	31400360	G	A	0.351	-0.070	0.012	4656	1.80E-08	NO	NA	NA
ENSG00000169877	AHSP	rs9930811	16	31400360	G	A	0.351	0.069	0.012	31346	2.67E-08	NO	NA	NA
ENSG00000140688	C16orf58	rs9930811	16	31400360	G	A	0.351	0.064	0.012	31346	2.46E-07	NO	NA	NA
ENSG00000099377	HSD3B7	rs9930811	16	31400360	G	A	0.351	-0.063	0.012	31346	3.74E-07	NO	NA	NA
ENSG00000140691	ARMC5	rs35445454	16	31699326	T	C	0.336	0.064	0.013	26049	3.80E-07	NO	NA	NA
ENSG00000103507	BCKDK	rs35445454	16	31699326	T	C	0.336	-0.061	0.013	26798	1.39E-06	NO	NA	NA
ENSG00000169877	AHSP	rs35445454	16	31699326	T	C	0.336	0.060	0.013	26798	1.59E-06	NO	NA	NA
ENSG00000169896	ITGAM	rs9930811	16	31400360	G	A	0.351	-0.056	0.012	31346	5.98E-06	NO	ROVELIZUMAB	Treat paroxysmal nocturnal hemoglobinuria
ENSG00000140675	SLC5A2	rs9930811	16	31400360	G	A	0.351	0.052	0.012	31306	3.05E-05	NO	SGLT2 inhibitor	Treat diabetes

Notes: The expression data of gene were obtained from whole blood. Gene ID and Gene refers to each gene in the nearby genomic region. Variant ID, CHR and Position are the ID, chromosome and position of the genetic variant. Effect allele, other allele, effect allele freq, beta, SE, N and P are the genetic association information of the genetic variant on the expression of the related gene. Known glycemic gene, drug and function are the annoations of related gene, which refers to whether the gene is a reported gene for any glycemic traits, whether the gene has any interaction with any drug as well as the function of the related drug.

Supplementary Table 12. Association between use of SGLT2i compared with DPP4i and risk of prostate cancer. Related to STAR Methods

	Original cohort							PS-matched cohort						
	SGLT2i			DPP4i			Hazard ratio (95% CI)	SGLT2i			DPP4i			Hazard ratio (95% CI)
	Events	Person years	Incidence rate	Events	Person years	Incidence rate		Events	Person years	Incidence rate	Events	Person years	Incidence rate	
Main analysis	114	25171.09	452.90	574	102208.72	561.60	0.76 (0.61-0.94)	106	22678.6	467.4	224	45458.9	492.75	0.77 (0.61-0.99)
Lag period														
1-month	85	25119.20	338.39	451	102153.20	441.49	0.86 (0.67-1.10)	80	22662.70	353	182	44455.1	409.40	0.81 (0.61-1.07)
2-month	74	24952.08	296.57	423	101978.74	414.79	0.83 (0.64-1.09)	69	22585	305.51	172	43573.6	394.73	0.75 (0.56-1.00)
3-month	67	24733.13	270.89	408	101759.12	400.95	0.81 (0.62-1.07)	63	22425.8	280.93	164	42548.9	385.44	0.78 (0.57-1.06)
6-month	49	23449.53	208.96	339	100444.48	337.50	0.90 (0.66-1.25)	46	21423.9	214.71	143	38982.9	366.83	0.75 (0.53-1.08)

Notes: prostate cancer refers to individuals with incident prostate cancer plus those with total prostate specific antigen [PSA] level>10 ng/mL during the follow-up period SGLT2i. PS-matched cohort refers to 1:1 propensity-score matching cohort of 48,310 patients, sodium-glucose cotransporter-2 inhibitor; CI, confidence interval. The unit of the incidence rate was 100,000 person years.

Supplementary Table 13. Association between HBA1c levels and type 2 diabetes with total prostate cancer. Related to STAR Methods

Supplementary Table 13A. Mendelian randomization estimates of HBA1c levels on total prostate cancer. Related to STAR Methods

exposure	outcome	method	nsnp	b	se	pval	Q	Q_df	Q_pval	lci	uci	OR (inhibitio	OR_UCI	OR_LCL
Glycated haemoglobin (UK Biobank)	Prostate cancer (PRACTICAL)	Inverse variance weighted	287	0.016	0.032	0.629	992.021	286	1.15E-78	-0.048	0.079	0.984	1.049	0.924
Glycated haemoglobin (UK Biobank)	Prostate cancer (PRACTICAL)	Weighted median	287	-0.029	0.034	0.394	NR	NR	NR	-0.096	0.038	1.029	1.100	0.963
Glycated haemoglobin (UK Biobank)	Prostate cancer (PRACTICAL)	Simple mode	287	-0.009	0.073	0.899	NR	NR	NR	-0.151	0.133	1.009	1.163	0.876
Glycated haemoglobin (UK Biobank)	Prostate cancer (PRACTICAL)	Weighted mode	287	-0.009	0.031	0.768	NR	NR	NR	-0.071	0.052	1.009	1.073	0.949
Glycated haemoglobin (MAGIC)	Prostate cancer (PRACTICAL)	Inverse variance weighted	91	0.004	0.019	0.816	272.549	90	2.98E-20	-0.033	0.042	0.996	1.034	0.959
Glycated haemoglobin (MAGIC)	Prostate cancer (PRACTICAL)	Weighted median	91	0.003	0.020	0.879	NR	NR	NR	-0.036	0.042	0.997	1.037	0.958
Glycated haemoglobin (MAGIC)	Prostate cancer (PRACTICAL)	Simple mode	91	0.034	0.038	0.384	NR	NR	NR	-0.042	0.109	0.967	1.043	0.897
Glycated haemoglobin (MAGIC)	Prostate cancer (PRACTICAL)	Weighted mode	91	0.006	0.022	0.791	NR	NR	NR	-0.038	0.050	0.994	1.039	0.951
Glycated haemoglobin without SGLT2 variant (UK Biobank)	Prostate cancer (PRACTICAL)	Inverse variance weighted	268	-0.007	0.024	0.778	813.157	267	1.58E-56	-0.054	0.041	1.007	1.056	0.960
Glycated haemoglobin without SGLT2 variant (UK Biobank)	Prostate cancer (PRACTICAL)	Weighted median	268	-0.021	0.027	0.446	NR	NR	NR	-0.074	0.033	1.021	1.077	0.968
Glycated haemoglobin without SGLT2 variant (UK Biobank)	Prostate cancer (PRACTICAL)	Simple mode	268	-0.023	0.060	0.700	NR	NR	NR	-0.141	0.094	1.023	1.151	0.910
Glycated haemoglobin without SGLT2 variant (UK Biobank)	Prostate cancer (PRACTICAL)	Weighted mode	268	-0.013	0.023	0.581	NR	NR	NR	-0.057	0.032	1.013	1.059	0.969

Supplementary Table 13B. Association of observed HbA1c (mmol/mol) with prostate cancer incidence in 165,430 men with European ancestry from UK Biobank. Related to STAR Methods

Cox model	Considering competing risk in the model	N cases/N control	HR (95% CI) per 1 increase in HbA1c
Crude	No	7986/157,444	1.02 (1.01, 1.02)
Crude	Yes (13,192 men dead due to other reasons before 2022-02-01)	7986/157,444	1.02 (1.01, 1.03)
Confounder-adjusted	No	7789/153,633	0.99 (0.99, 1.00)
Confounder-adjusted	Yes (12,498 men dead due to other reasons before 2022-02-01)	7789/153,633	0.99 (0.99, 1.00)

Note: age, BMI, smoking status, alcohol consumption, physical activity and diet score were adjusted as covariates in the Cox model.

Supplementary Table 13C. Association of baseline type 2 diabetes with prostate cancer incidence in men with East Asian ancestry from 4C. Related to STAR Methods

	NGR	IGR	T2DM
Events	36	103	74
Incidence rate (per 1000 person-year)	0.32 (0.23-0.45)	0.36 (0.30-0.44)	0.47 (0.37-0.60)
Multivariable adjusted HR (95%CI)	1.0 (reference)	0.93 (0.61-1.42)	1.13 (0.72-1.75)
Multivariable adjusted HR (95%CI)*	1.0 (reference)	0.92 (0.61-1.40)	1.09 (0.70-1.70)

Note: NGR, normal glucose regulation; IGR, impaired glucose regulation; T2DM, Type 2 diabetes mellitus. Adjusted for age, gender, body-mass index, family history of diabetes, smoking, drinking, high school or above education, moderate or vigorous physical activity, diet score, systolic blood pressure, LDL-cholesterol. *Excluding prostate cancers occur during the first year.

Supplementary Table 14. MELODI Preso results to identify potential intermediate traits that linking SGLT2 inhibitors with prostate cancer. Related to STAR Methods

X Subject	X Pval	X Predicate	Overlap	Y Predicate	Y Object	Y Pval	Y
Sodium-Glucose Transporter 2 Inhibitors	0.00E+00	TREATS	Obesity	AUGMENTS	Urinary Incontinence	#######	prostate_cancer
dapagliflozin	0.00E+00	TREATS	Obesity	AUGMENTS	Urinary Incontinence	#######	prostate_cancer
dapagliflozin	6.80E-09	STIMULATES	FRAP1 protein, human|MTOR	COEXISTS_WITH	FLVCR1	#######	prostate_cancer
canagliflozin	6.80E-09	STIMULATES	Heme Oxygenase-1	COEXISTS_WITH	FLVCR1	#######	prostate_cancer
canagliflozin	6.80E-09	STIMULATES	Heme Oxygenase-1	INTERACTS_WIT	FLVCR1	#######	prostate_cancer
canagliflozin	6.80E-09	STIMULATES	Heme Oxygenase-1	STIMULATES	VEGF protein, human|VEGFA	#######	prostate_cancer
Licogliflozin	6.80E-09	TREATS	Obesity	AUGMENTS	Urinary Incontinence	#######	prostate_cancer
ertugliflozin	6.80E-09	TREATS	Obesity	AUGMENTS	Urinary Incontinence	#######	prostate_cancer
empagliflozin	2.92E-06	INTERACTS_WIT	cytokine	STIMULATES	Androgen Receptor|AR	#######	prostate_cancer
empagliflozin	2.92E-06	STIMULATES	Heme Oxygenase-1	COEXISTS_WITH	FLVCR1	#######	prostate_cancer
empagliflozin	2.92E-06	STIMULATES	Heme Oxygenase-1	INTERACTS_WIT	FLVCR1	#######	prostate_cancer
dapagliflozin	2.92E-06	TREATS	Coronary Arteriosclerosis	CAUSES	Congestive heart failure	#######	prostate_cancer
Sodium-Glucose Transporter 2 Inhibitors	2.92E-06	COEXISTS_WITH	Insulin	STIMULATES	Mitogen Activated Protein Kinase 1	#######	prostate_cancer
Sodium-Glucose Transporter 2 Inhibitors	2.92E-06	INHIBITS	Insulin	STIMULATES	Mitogen Activated Protein Kinase 1	#######	prostate_cancer
dapagliflozin	2.92E-06	COEXISTS_WITH	Insulin	STIMULATES	Mitogen Activated Protein Kinase 1	#######	prostate_cancer
empagliflozin	2.92E-06	STIMULATES	Heme Oxygenase-1	STIMULATES	VEGF protein, human|VEGFA	#######	prostate_cancer
canagliflozin	2.92E-06	TREATS	Obesity	AUGMENTS	Urinary Incontinence	#######	prostate_cancer
empagliflozin	2.92E-06	INTERACTS_WIT	cytokine	COEXISTS_WITH	FLVCR1	#######	prostate_cancer
